# Socceromics: A Systematic Review of Omics Technologies to Optimize Performance and Health in Soccer

**DOI:** 10.3390/ijms27020749

**Published:** 2026-01-12

**Authors:** Adam Owen, Halil İbrahim Ceylan, Piotr Zmijewski, Carlo Biz, Giovanni Sciarretta, Alessandro Rossin, Pietro Ruggieri, Andrea De Giorgio, Carlo Trompetto, Nicola Luigi Bragazzi, Luca Puce

**Affiliations:** 1 Centre de Recherche et d’Innovation sur le Sport, Université Claude Bernard Lyon.1, 69100 Lyon, France; aowen@rovers.co.uk; 2 Blackburn Rovers, Blackburn BB2 4JF, UK; 3 Physical Education and Sports Teaching Department, Faculty of Kazim Karabekir Education, Atatürk University, 25030 Erzurum, Turkey; halil.ceylan@atauni.edu.tr; 4 Jozef Pilsudski University of Physical Education in Warsaw, 00-968 Warsaw, Poland; piotr.zmijewski@insp.pl; 5 Orthopedics and Orthopedic Oncology, Department of Surgery, Oncology and Gastroenterology (DiSCOG), University of Padua, 35122 Padua, Italy; giovanni.sciarretta@gmail.com (G.S.); alessandro.rossin.mc@gmail.com (A.R.); pietro.ruggieri@unipd.it (P.R.); 6 Department of Psychology, eCampus University, 22060 Novedrate, Italy; andrea.degiorgio@uniecampus.it; 7 Department of Neuroscience, Rehabilitation, Ophthalmology, Genetics, Maternal and Child Health, University of Genoa, 16126 Genoa, Italy; ctrompetto@neurologia.unige.it (C.T.); luca1puce@gmail.com (L.P.); 8 Human Nutrition Unit (HNU), Department of Food and Drugs, University of Parma, 43121 Parma, Italy

**Keywords:** soccer, omics technologies, genomics, proteomics, metabolomics, microbiomics, integrative omics, sportomics, athlomics, socceromics

## Abstract

The integration of omics technologies, including genomics, proteomics, metabolomics, and microbiomics, has transformed sports science, particularly soccer, by providing new opportunities to optimize player performance, reduce injury risk, and enhance recovery. This systematic literature review was conducted in accordance with PRISMA 2020 guidelines and structured using the PICOS/PECOS framework. Comprehensive searches were performed in PubMed, Scopus, and Web of Science up to August 2025. Eligible studies were peer-reviewed original research involving professional or elite soccer players that applied at least one omics approach to outcomes related to performance, health, recovery, or injury prevention. Reviews, conference abstracts, editorials, and studies not involving soccer or omics technologies were excluded. A total of 139 studies met the inclusion criteria. Across the included studies, a total of 19,449 participants were analyzed. Genomic investigations identified numerous single-nucleotide polymorphisms (SNPs) spanning key biological pathways. Cardiovascular and vascular genes (e.g., *ACE*, *AGT*, *NOS3*, *VEGF*, *ADRA2A*, *ADRB1–3*) were associated with endurance, cardiovascular regulation, and recovery. Genes related to muscle structure, metabolism, and hypertrophy (e.g., *ACTN3*, *CKM*, *MLCK*, *TRIM63*, *TTN-AS1*, *HIF1A*, *MSTN*, *MCT1*, *AMPD1*) were linked to sprint performance, metabolic efficiency, and muscle injury susceptibility. Neurotransmission-related genes (*BDNF*, *COMT*, *DRD1–3*, *DBH*, *SLC6A4*, *HTR2A*, *APOE*) influenced motivation, fatigue, cognitive performance, and brain injury recovery. Connective tissue and extracellular matrix genes (*COL1A1*, *COL1A2*, *COL2A1*, *COL5A1*, *COL12A1*, *COL22A1*, *ELN*, *EMILIN1*, *TNC*, *MMP3*, *GEFT*, *LIF*, *HGF*) were implicated in ligament, tendon, and muscle injury risk. Energy metabolism and mitochondrial function genes (*PPARA*, *PPARG*, *PPARD*, *PPARGC1A*, *UCP1–3*, *FTO*, *TFAM*) shaped endurance capacity, substrate utilization, and body composition. Oxidative stress and detoxification pathways (*GSTM1*, *GSTP1*, *GSTT1*, *NRF2*) influenced recovery and resilience, while bone-related variants (*VDR*, *P2RX7*, *RANK/RANKL/OPG)* were associated with bone density and remodeling. Beyond genomics, proteomics identified markers of muscle damage and repair, metabolomics characterized fatigue- and energy-related signatures, and microbiomics revealed links between gut microbial diversity, recovery, and physiological resilience. Evidence from omics research in soccer supports the potential for individualized approaches to training, nutrition, recovery, and injury prevention. By integrating genomics, proteomics, metabolomics, and microbiomics data, clubs and sports practitioners may design precision strategies tailored to each player’s biological profile. Future research should expand on multi-omics integration, explore gene–environment interactions, and improve representation across sexes, age groups, and competitive levels to advance precision sports medicine in soccer.

## 1. Introduction

Soccer is one of the most physically demanding sports, requiring peak physical fitness alongside advanced mental and tactical skills [1,2]. During a typical 90-min match, elite players cover approximately 10 km [1]. This level of cardiovascular exertion demonstrates the balance players must maintain between endurance and high-intensity bursts of energy throughout the game. Soccer incorporates a variety of physical activities, including sprinting, tackling, and jumping, each of which demands energy in distinct ways. Sprinting calls for explosive power and speed, while tackling and jumping rely on muscular strength, coordination, and agility [3,4]. These high-intensity efforts are typically interspersed with lower-intensity movements like jogging or walking, allowing players brief recovery periods before resuming peak activity.

In addition to physical endurance and strength, players must maintain sharp mental focus and tactical awareness. Decision-making, anticipation, and positioning are critical as teams constantly transition between offensive and defensive phases. This mental agility enables players to execute strategies effectively and react to opponents’ actions under physical fatigue. The dual demands on aerobic and anaerobic energy systems in soccer underscore the complexity of training regimens, which must develop endurance, strength, speed, and mental resilience. Players also undergo tactical training that enhances their ability to make quick, effective decisions while maintaining physical performance, creating a game that tests an athlete’s holistic physical and cognitive capacities [5].

With advancements in sports science, omics technologies—including genomics, metabolomics, proteomics, and microbiomics—have attracted considerable interest in soccer due to their potential to provide a comprehensive understanding of an athlete’s physiological state. These technologies offer insights into how genetic, molecular, and biochemical/physiological profiles impact performance, injury risk, recovery, and adaptation to training [6,7,8,9,10].

However, soccer science still faces challenges in addressing individual variations in player performance, recovery needs, and injury susceptibility. Omics technologies hold promise in addressing these limitations. For instance, genomics could improve injury prevention by identifying players genetically predisposed to specific injuries, enabling customized training and recovery plans. Proteomics could offer real-time insights into muscle recovery by monitoring biomarkers associated with tissue repair and inflammation, while metabolomics may optimize endurance and energy strategies by analyzing a player’s metabolic profile. Additionally, microbiomics could inform gut health and nutrition strategies, essential for maintaining peak physical condition and enhancing recovery.

The concept of “socceromics” refers to the integration of these omics technologies in soccer to enhance both performance and health. This shift toward personalized, data-driven interventions provides clubs and sports practitioners with an unprecedented opportunity to tailor strategies to individual players’ physiological profiles, potentially improving outcomes both on and off the pitch. By harnessing each omics field’s unique contributions, this approach can help build individualized, data-driven strategies to optimize performance and health for each athlete.

## 2. Materials and Methods

### 2.1. Study Design

This systematic review was conducted in accordance with the Preferred Reporting Items for Systematic Reviews and Meta-Analyses (PRISMA) 2020 guidelines [11], and structured using the Population, Intervention/Exposure, Comparator, Outcomes, and Study design (PICOS/PECOS) framework. Given the heterogeneity of omics methodologies and study designs, a quantitative meta-analysis was not feasible; therefore, a narrative critical synthesis was performed. All steps of the review (search strategy, study selection, data extraction, and reporting) were pre-defined in a protocol registered on Open Science Framework (OSF identifier: DOI 10.17605/OSF.IO/E6TQ3). The completed PRISMA checklist is provided in Table S1 of the Supplementary Materials.

### 2.2. Research Questions

Our research question explores how a multidisciplinary approach—integrating genomics, proteomics, metabolomics, and microbiomics data—can advance personalized strategies to optimize performance, prevent injuries, and accelerate recovery in elite soccer players. To address this question, we assembled a uniquely skilled team, bringing together experts from sports science, clinical disciplines, such as orthopedics, rehabilitation, and physiatry, as well as genetics, molecular biology, nutrition, neuroscience, and psychology, spanning diverse institutions worldwide. This international and interdisciplinary collaboration allows for a thorough comprehension of omics data, with each team member contributing specialized knowledge. Our shared objective is to understand how to design and implement targeted interventions tailored to each athlete’s genetic, post-genetic, and biochemical/physiological profile, transforming scientific insights into actionable strategies that support player health and maximize performance in the demanding physical and mental environment of elite soccer.

### 2.3. Search Strategy

A comprehensive search was conducted in MEDLINE/PubMed, Scopus, and Web of Science (WoS) for articles published up to 25 August 2025. No filters regarding language, publication date, document type, competition level, or other player characteristics, were applied during the primary search to maximize sensitivity and ensure broad capture of potentially relevant literature. Searches were performed using “UNO per tutto,” the Artificial Intelligence (AI)-enhanced discovery platform of the University of Genoa. Search terms combined Medical Subject Headings (MeSH) and free-text keywords related to soccer and omics technologies, including: soccer, football, genomics, polymorphisms, Single Nucleotide Polymorphisms (SNPs), genetic variants, Copy Number Variants (CNVs), Single Nucleotide Variants (SNVs), allele, metabolome, metabolomics, microbiome, microbiomics, microbiota, proteome, proteomics, methylome, methylomics, epigenome, epigenetics, epigenomics, sportomics, and athlomics. The detailed search strings for each database are provided in Supplementary Materials S2. The search strategy was developed by a research methodologist with expertise in systematic reviews and meta-analyses (N.L.B.), following an initial review of the relevant literature and with the assistance of a librarian.

### 2.4. Eligibility Criteria

Study eligibility was defined according to the PICOS/PECOS framework. During the initial database search, no filters (language, date, document type, competition level, or other player characteristics) were applied to ensure maximal sensitivity. All eligibility criteria were subsequently applied during the screening phase.

**Inclusion criteria**

Studies were included if they met the following conditions:

- Population: Human participants who were professional, elite, or academy-level soccer players.
- Intervention/Exposure: Application of at least one omics technology, including genomics, epigenomics, proteomics, metabolomics, lipidomics, or microbiomics.
- Comparator: Any comparator or none; no restrictions were imposed.
- Outcomes: Findings related to performance, biochemical/physiological adaptation, injury risk, recovery, or health-related biomarkers.
- Study design: Original peer-reviewed research articles (observational, interventional, cross-sectional, longitudinal, or multi-omics).

**Exclusion criteria**

Studies were excluded if they met any of the following conditions:

- Non-human or in vitro studies.
- Studies not involving soccer players.
- Articles lacking an omics component (traditional biochemistry/physiology only).
- Non-original/non-peer-reviewed works (reviews, systematic reviews, editorials, commentaries, conference abstracts, theses/dissertations).
- Non-English full texts or articles without an accessible full text.
- Case reports or anecdotal evidence.

These criteria ensured that the review focused exclusively on human soccer research involving omics-based approaches, in alignment with the research objectives.

### 2.5. Study Selection

All records retrieved from the databases were first imported into EndNote X9 for initial duplicate identification and removal. The cleaned library was then exported into the Covidence systematic review software (Veritas Health Innovation, Melbourne, Australia; web-based application; available at https://www.covidence.org; accessed on 26 August 2025), where Covidence’s automated duplicate detection tool performed an additional check to ensure complete deduplication.

The remaining unique records underwent an independent screening process conducted by two reviewers (A.O. and L.P.) according to the predefined eligibility criteria. Screening occurred in two stages: (i) title and abstract screening and (ii) full-text assessment. Any disagreements between the reviewers were resolved through discussion, and when necessary, a third reviewer (N.L.B.) served as the adjudicator to reach consensus.

### 2.6. Data Extraction

Data extraction focused on capturing key information related to study design, participant characteristics, sample collection methods, and omics technologies used. Extracted data included: (i) sample sizes and populations (e.g., professional soccer players, elite athletes), (ii) type of omics analysis (e.g., genomics, proteomics, metabolomics, microbiomics), (iii) specific markers or genes studied (e.g., *ACTN3*, *COL1A1*, metabolic biomarkers), (iv) results, including associations between omics data and performance, injury risk, or recovery times, and (v) methodological limitations noted in the studies.

Data extraction was independently conducted by two researchers (A.O. and L.P.), with any potential disagreements resolved by a third researcher (N.L.B.), who acted as the final referee.

### 2.7. Data Synthesis

Due to heterogeneity across study designs, populations, and outcomes, a qualitative narrative synthesis was conducted. Studies were grouped by omics technology (genomics, proteomics, metabolomics, microbiomics). Evidence was critically appraised in terms of methodological strengths, limitations, and translational potential for soccer-specific applications.

### 2.8. Risk of Bias and Study Quality Assessment

Given the substantial methodological heterogeneity across omics domains, we assessed study quality using a custom modified version of the Quality Assessment of Diagnostic Accuracy Studies using OMICS-based technologies (QUADOMICS) tool, an approach previously adopted in omics-based systematic reviews [12,13]. The modified checklist (Supplementary Table S3) included key items relevant to omics research, such as clarity of selection criteria, description of sample type, timing and procedures of sample collection, pre-analytical handling, replicability of the analytical workflow, use of reference standards, reporting of uninterpretable results, and avoidance of overfitting. Each study was independently rated by two reviewers (A.O. and L.P.) and classified as having low, moderate, or high methodological quality.

## 3. Results

### 3.1. Search Results

The systematic search across MEDLINE/PubMed (*n* = 277), WoS (*n* = 329), and Scopus (*n* = 362) initially identified 968 records. After removing 420 duplicates, 548 unique records remained for screening. Following title and abstract screening, 391 records were excluded for not meeting the eligibility criteria, leaving 157 full-text articles for detailed assessment. Of these, 18 reports were excluded with reason—six due to the wrong study design (e.g., meeting or conference abstracts), four due to the wrong intervention/exposure, four because no full English text was available, and four due to the wrong population. Ultimately, 139 studies were included in the systematic review.

**Figure 1:**
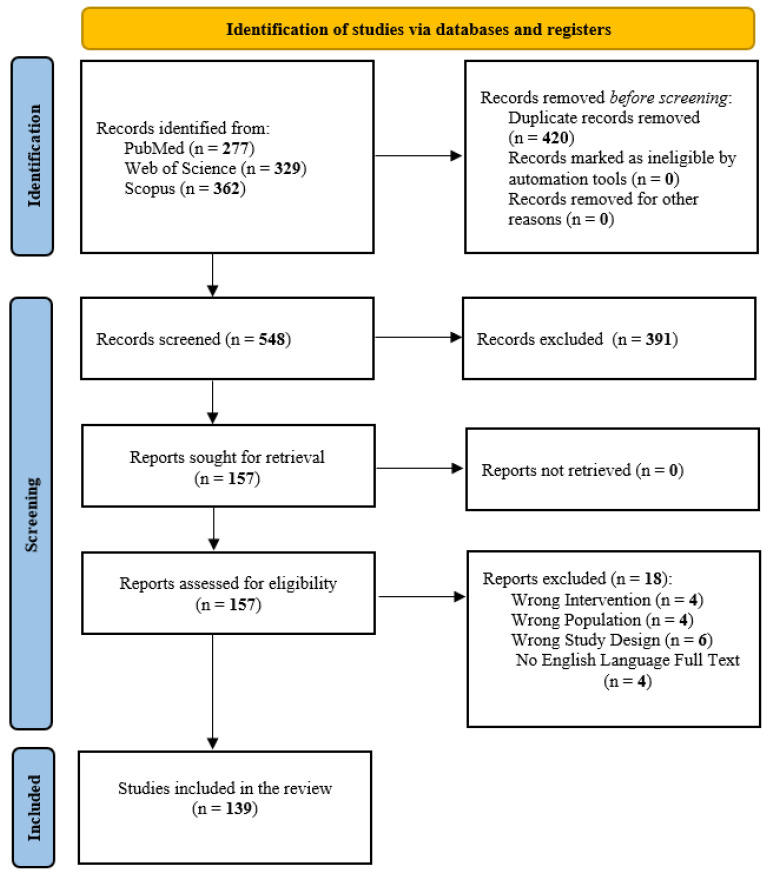
PRISMA 2020 flow diagram of the study selection process.

The study selection process is illustrated in the PRISMA 2020 flow diagram (Figure 1).

### 3.2. Study Characteristics

**Figure 2:**
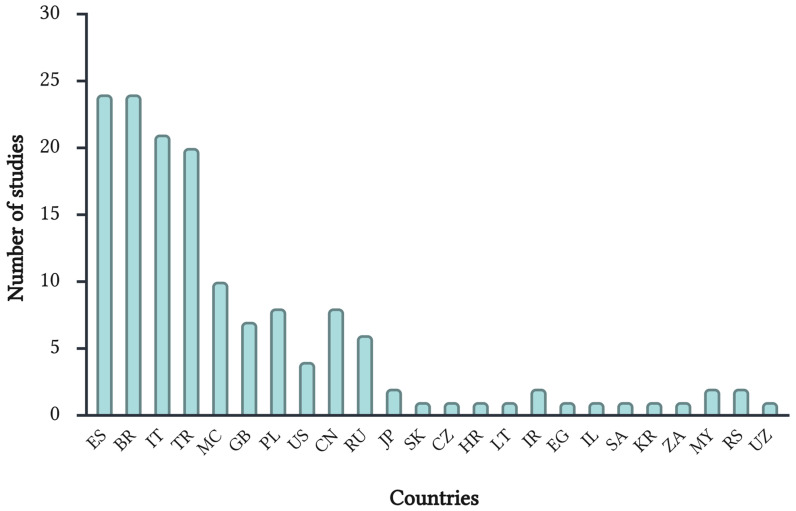
Geographic distribution of the included omics studies in soccer. MC: Multi-Country.

A total of 139 studies met the inclusion criteria and were included in this review [14,15,16,17,18,19,20,21,22,23,24,25,26,27,28,29,30,31,32,33,34,35,36,37,38,39,40,41,42,43,44,45,46,47,48,49,50,51,52,53,54,55,56,57,58,59,60,61,62,63,64,65,66,67,68,69,70,71,72,73,74,75,76,77,78,79,80,81,82,83,84,85,86,87,88,89,90,91,92,93,94,95,96,97,98,99,100,101,102,103,104,105,106,107,108,109,110,111,112,113,114,115,116,117,118,119,120,121,122,123,124,125,126,127,128,129,130,131,132,133,134,135,136,137,138,139,140,141,142,143,144,145,146,147,148,149,150,151,152]. The publication years ranged from 2008 to 2025, reflecting the progressive expansion of omics technologies in soccer research over the past two decades, with a clear increase in output in the most recent years. Across the 139 included studies, a total of 19,449 participants were analyzed, with sample sizes ranging from 10 to 710 athletes, encompassing both youth and adult male and female players. Although study designs were heterogeneous, comprising cross-sectional analyses, randomized controlled trials, longitudinal cohort investigations, and pilot interventional studies, the field was dominated by cross-sectional genetic association studies, which represented the primary methodological approach used to explore genotype–phenotype relationships in soccer. There was a clear overrepresentation of studies conducted in male players, with men comprising the vast majority of cohorts, whereas only a small fraction of studies included female footballers. The geographical distribution of the included studies was highly uneven, with a strong concentration of omics research originating from European and South American countries, particularly Spain, Brazil, Italy, and Turkey, while contributions from other regions were comparatively scarce (Figure 2).

In terms of omics domains, genomics accounted for the largest proportion of studies, primarily investigating SNPs in candidate genes such as *ACE*, *ACTN3*, *COL5A1*, *AMPD1*, and *VDR*. Among these, *ACTN3* has emerged as the most extensively studied gene, consistently linked to strength, power, endurance, and injury susceptibility in soccer players [22,54,56,63,76,81,83,88,91,93,95,97,106,108,116,119,129,133,134,141,145,152].

Metabolomics studies frequently employed nuclear magnetic resonance spectroscopy (NMR) or mass spectrometry (MS) to profile energy metabolism, amino acid turnover, and oxidative stress markers, often in relation to match play or training load. Proteomics analyses were less frequent but provided insights into inflammation, muscle repair, and recovery biomarkers. Microbiomics studies investigated gut microbial composition through 16S rRNA sequencing, mainly in response to dietary interventions (e.g., probiotics, kefir) or exercise training. A smaller number of studies applied epigenomics, particularly DNA methylation clocks, to monitor biological aging and recovery dynamics. 

Gene names, aliases, and associated polymorphisms were standardized according to the HUGO Gene Nomenclature Committee (HGNC) database [153], and both official symbols and commonly used aliases are reported in Table 1.

Comprehensive details on study characteristics, participant demographics, omics technologies used, tissue or sample types, and analytical platforms or pipelines are provided in Table S4 of the Supplementary Materials.

### 3.3. Genomics in Soccer

Genomic profiling is rapidly becoming an essential tool in sports science, helping to predict and enhance performance while minimizing injury risk [102,132,154,155,156,157,158]. The study of genetic polymorphisms has demonstrated that certain genetic variants can influence key athletic traits as well as predispose individuals to injuries (Table 1).

### 3.4. Cardiovascular System-Related Polymorphisms in Soccer

Cardiovascular and vascular-response genes have been among the most extensively investigated polymorphisms in soccer, given their direct role in regulating blood pressure, vascular tone, and oxygen delivery during high-intensity intermittent exercise. The *ACE* gene, which encodes the angiotensin-converting enzyme (ACE), plays a crucial role in regulating blood pressure and maintaining fluid balance through the renin-angiotensin system (RAS) [81,159]. Its polymorphisms have been widely studied in soccer players, with consistent associations to athletic performance in both men [22,26,33,44,93,138] and women [63,70,160] as well as to supplementation responses such as creatine intake [109]. Specifically, the polymorphic allele *I* correlates with improved resistance and endurance, the allele *D* with enhanced strength and speed, and the heterozygous genotype with athletic power [79,134,161,162]. A systematic review and meta-analysis indicated a higher prevalence of the *ACE* D allele among youth footballers with an odds-ratio, OR, of 1.18 (95% confidence interval, CI, 1.01–1.38) and the *ACE* DD genotype showing the strongest association (OR 1.29, 95% CI 1.02–1.63) [163]. Additionally, the *ACE* I/I genotype was more prevalent among older players in the U17 category, particularly defenders [105]. *ACE* polymorphisms were linked with improved squat, vertical jump, and countermovement jump performance [22,27]. In some studies [117,164,165,166,167], the *ACE* genotype was associated with cardiac morphology. Moreover, soccer players with the *ACE* II genotype were reported to be more prone to muscle injuries, likely due to genetic influences on muscle recovery and performance [88]. While associations were reproduced in Turkish and Japanese players [60,129], they were absent in Korean [168], Brazilian [39] male footballers, nor with muscle power in Chinese youth male players [91,95], highlighting possible ethnic variability.

Closely related to ACE in the RAS, the angiotensinogen (*AGT*) gene contributes to blood pressure regulation, electrolyte balance, and vascular tone [169,170]—all of which are essential for physical performance, particularly in sports like soccer that demand both endurance and explosive power. The *AGT* gene, particularly the rs699 polymorphism, has been studied for its potential influence on athletic performance. However, contrasting findings are reported in the literature. While a few studies could find associations with performance, cardiac, and hemodynamic parameters [64], these results have not been replicated among some specific soccer player populations, such as Brazilian ones [52].

The *NOS3* gene encodes the nitric oxide synthase 3 (NOS3), which produces nitric oxide (NO), influencing vascular tone and endurance performance [76,115,145,171,172]. The *NOS3* -786 T/C polymorphism (rs2070744) has been identified as a potential genetic marker influencing individual variations in sports-related phenotypes. In a study [29], significant differences in genotype frequencies were observed between the soccer players and all other groups (controls, endurance athletes, and power athletes), with all comparisons yielding statistically significant results. Additionally, allele frequency analysis further confirmed these differences. These findings could be replicated in an Italian cohort [173]. Of note, the *NOS3* Glu298Asp (rs1799983) polymorphism was associated with noncontact hamstring muscle injury risk in a sample of elite male outfield players [57], while, in another study [90], the *NOS3* Glu/Glu allele was found to predispose players to the attacker and defender positions in elite soccer, as these roles are associated with greater strength and power metrics compared to midfielders.

Finally, the *VEGF* gene encodes the vascular endothelial growth factor (VEGF), which stimulates blood vessel formation, linked to oxygen transport and endurance. As such, it plays a crucial role in athletic performance by promoting improved vascularization, which supports endurance by increasing capillary density and aiding adaptation to aerobic training. *VEGF* also accelerates muscle repair and recovery from injuries by promoting tissue regeneration. Additionally, the gene responds to low oxygen conditions, such as altitude training, enhancing performance by boosting oxygen-carrying capacity. The rs2010963 polymorphism has been found to enrich the genetic fingerprinting of professional athletes [171] and, along with other SNPs, it has been associated with performance outcomes after training interventions [32]. In addition to these canonical cardiovascular genes, several adrenergic receptor variants—central to cardiorespiratory regulation—have been examined in soccer. *ADRA2A*, *ADRB1* (rs1801253), *ADRB2* (rs1042713, rs1042714), and *ADRB3* (rs4994) influence sympathetic drive, bronchodilation, cardiac output, and lipid metabolism, thereby shaping aerobic efficiency and match-running capacity. These polymorphisms have been included in multi-SNP performance models in elite cohorts, with evidence suggesting their contribution to interindividual variability in endurance profiles and stress-response regulation (Table 1).

### 3.5. Muscle Structure and Function-Related Polymorphisms in Soccer

*ACTN3*, also known as the “speed gene”, has been linked to sprinting and explosive power, as well as to performance metrics, even if to a varying extent [63,88,97,119,133,172]. Players with the RR genotype tend to have greater strength and speed, whereas those with the XX genotype (α-actinin-3 deficiency) may excel in endurance activities but are more susceptible to injuries. In a study [65], players with the *ACTN3* XX genotype had 2.66 times higher odds of injury than those with the RR genotype, while RX and RR players had similar injury incidences. Additionally, XX players had 2.13 times higher odds of severe injuries than RR players, and RX individuals had 1.63 times higher odds of severe injuries than RR players. Further research [108] provides further compelling evidence that players with the *ACTN3* XX genotype are more prone to muscle injuries and have reduced running performance. On the other hand, in some studies [56,69], which reviewed non-contact musculoskeletal soft-tissue injuries in professional football players, while a significant association was found between injury rate and the *ACTN3* genotype, no statistically significant differences were observed in injury severity or recovery time linked to the *ACTN3* SNP. Furthermore, the *ACTN3* polymorphism can influence processes such as muscle recovery in professional soccer players [106], with a significant positive correlation found between the number of sprints and creatine kinase levels. This correlation could be noted in players with the *ACTN3* RR genotype, likely due to the presence of more type II muscle fibers, while no such relationship was observed in X allele carriers.

Finally, interactions between *ACE* and *ACTN3* genotypes have been reported, although their combined effect explains only a modest proportion of performance variance [28].

The *CKM* gene, or muscle-specific creatine kinase (CK) gene, plays a vital role in energy metabolism within muscle tissue, particularly in skeletal muscles and the heart. The enzyme it encodes, muscle-specific CK, is essential for the phosphocreatine system, which facilitates the rapid regeneration of adenosine triphosphate (ATP) during muscle contraction. This system becomes especially important during short bursts of high-intensity activities, such as sprinting or weightlifting, where immediate energy is crucial. Muscle-specific CK converts creatine and ATP into phosphocreatine and adenosine diphosphate (ADP), providing an energy reserve that can be quickly accessed during muscle contractions. This process supports not only explosive muscle movements but also recovery, helping maintain ATP levels and reducing fatigue during intense exercise. In a study [80], the rs8111989 polymorphism in the *CKM* gene was found to have an influence on injury incidence among high-performance football players. Despite genotyping players into GG, GA, and AA groups, overall injury incidence did not differ significantly across these genotypes during training or match exposure. However, certain injury patterns were associated with specific genotypes. GG players experienced a higher frequency of slight-severity injuries and muscle contractures, while GA players were more prone to severe injuries and muscle tears. Additionally, G allele carriers had fewer gradual-onset and recurrent injuries compared to AA players, while A allele carriers had a higher frequency of severe injuries than GG players.

Finally, the *MLCK* gene encodes the myosin light chain kinase, which regulates muscle contraction. It has been found to be associated with an athlete’s professional status [76], while the *MYLK* (rs28497577) gene encoding the myosin light chain kinase and linked to muscle contraction and force generation has been found to be associated with muscle injuries in soccer players.

The *TRIM63* (*MuRF-1*) gene, encoding a muscle-specific E3 ubiquitin ligase, regulates muscle protein turnover and mass. In Brazilian professionals, the rs2275950 (A/G) polymorphism was tested for associations with muscle injuries, but no significant links were observed, suggesting limited biomarker value [111].

Similarly, the *TTN-AS1* gene, which influences sarcomere integrity, was investigated in U20 Brazilian soccer players. The rs1001238 CC genotype was associated with elevated inflammatory markers (neutrophils, C-reactive protein (CRP), and tumor necrosis factor alpha (TNFα)) and greater muscle damage (CK levels) compared to TT and TC genotypes, indicating a higher susceptibility to eccentric-exercise-induced injury [107]. Several additional genes involved in muscle metabolism, hypertrophy, and damage were also identified across soccer studies, but had been underrepresented in earlier summaries and genetic panels. These include *HIF1A*, which regulates hypoxia adaptation and has been associated with advantageous power-related phenotypes; *MSTN* (myostatin), where variants such as K153R, E164K, P198A, and I225T influence muscle hypertrophy potential and appear in polygenic performance models; *MCT1* (rs1049434), a key lactate transporter whose AA genotype has been linked to the highest incidence of muscle injuries in elite players; and *AMPD1* (rs17602729), which modulates energy turnover during high-intensity exercise and influences creatine responsiveness and lactate accumulation, with CT carriers demonstrating more favorable metabolic adaptations (Table 1).

### 3.6. Neurotransmission-Related Polymorphisms in Soccer

The *ADRA2A* and *ADRB2* genes, which are both associated with cardiorespiratory fitness, have been found to play a pivotal role in determining professional athlete status in a cohort of professional football players compared to non-athlete individuals [89]. These genes regulate, indeed, critical physiological functions such as heart rate, blood flow, and oxygen delivery during exercise. Variants in *ADRA2A*, encoding the alpha-2A adrenergic receptor, help modulate vascular tone and blood pressure, optimizing oxygen distribution during physical activity. Similarly, the *ADRB2* gene, encoding the beta-2 adrenergic receptor, influences bronchodilation and cardiovascular response, allowing athletes to achieve superior oxygen transport and utilization. These genetic variants contribute to enhanced endurance and stamina as observed in elite athletes. Additional adrenergic variants such as *ADRB1* (rs1801253) and *ADRB3* (rs4994), which affect cardiac output and lipolysis, respectively, have also been reported in soccer cohorts but were absent from earlier summaries and genetic panels.

The *BDNF* gene encodes the brain-derived neurotrophic factor (BDNF), which is linked to neurogenesis, besides being associated with muscle repair, regeneration, and growth [174]. In a study [40], *BDNF* Val66Met polymorphism was found to mediate the relationship between soccer heading and white matter microstructure in the brain.

Other genes related to neurotransmission that have been implicated in soccer science are the *DRD1* gene, the *DRD2* gene, and the *DBH* gene. The former encodes the dopamine receptor D1, which is involved in motivation, reward, and physical activity levels, while the latter encodes the dopamine beta-hydroxylase (DBH), which influences the conversion of dopamine to norepinephrine, linked to exercise performance and motivation [94].

The serotonin transporter gene (*5HTT*, L/S polymorphism) further modulates nervous system activity. Among young soccer players, the L/S genotype (~60%) was associated with balanced nervous activity and superior performance, whereas the SS genotype (~20%) correlated with aggression and nervous system mobility, and the LL genotype showed intermediate nervous activity with average simple visual-motor reaction (SVMR). Notably, the TT mutant variant predisposed players to faster fatigue and reduced adaptation to training loads. Similarly, the serotonin receptor *5HT2A* (rs6311) was studied in elite soccer players, where CC and CT genotypes (together ~60%) were linked with higher SVMR values, greater attentional stability, and the mobile nervous activity type considered favorable for performance in dynamic sports [135].

Based on the observation that, as previously mentioned, elite professional soccer players have been found to display a preponderance of an allele associated with the NOS enzyme, resulting in lower levels of nitric oxide compared to endurance athletes, power athletes, and sedentary men. Landers and Esch [175] suggested that this may reflect soccer’s nature as an “externally-paced” sport, highly dependent on teamwork and executive function skills. One aspect of executive function is the skill of time interval estimation, which relies heavily on dopamine, a neurotransmitter involved in cognitive functions. Polymorphisms that affect dopamine pathways, such as the *DRD2/ANKK1* Taq1a variant (which lowers D2 receptor density in the *striatum*, leading to increased dopamine synthesis), have been linked to both time interval estimation and executive skills. Genotypes among soccer players may influence cognitive abilities through neurotransmitter regulation, potentially predicting success in externally paced sports like soccer. Interestingly, dopamine levels show an inverted-U relationship with both time interval estimation accuracy and executive skills, suggesting that optimal dopamine levels are crucial for these abilities. Research has also revealed a pathway from dopamine to the production of small quantities of NO through endogenous morphine and mu3 receptors on endothelial cells, with exercise up-regulating dopamine and this pathway. Excessive exercise can lead to negative feedback, where NO downregulates dopamine to maintain optimal levels. In the context of professional soccer players, intense training, frequent public scrutiny, and stress can lead to elevated dopamine levels. However, without regulatory mechanisms, high dopamine could overwhelm the system. This is where the *NOS3* -786T/C polymorphism, carried by many elite players, may play a protective role. The C allele of this variant leads to reduced NO production, preventing excessive negative feedback on dopamine and maintaining the balance necessary for peak cognitive and physical performance.

Additional genes linked to neurocognitive performance include *APOE* (ε2/ε3/ε4), associated with brain resilience, where specific variants may increase vulnerability to neurodegeneration following repetitive heading exposure (Table 1).

These genetic adaptations may have evolutionary roots, with professional soccer players demonstrating a specialized mechanism for maintaining dopamine levels under intense physical and cognitive demands.

### 3.7. Connective Tissue and Tendon-Related Polymorphisms in Soccer

*COL1A1* is a gene that encodes the alpha-1 chain of type I collagen, which is the most abundant collagen in the human body. Type I collagen is a major structural protein found in skin, bone, tendons, ligaments, and other connective tissues, providing strength and support [143]. Ficek and colleagues [31] examined the association between *COL1A1* −1997G/T and +1245G/T polymorphisms and anterior cruciate ligament (ACL) ruptures in professional soccer players. The study compared 91 Polish or Eastern European descent (for at least three generations) male players with ACL ruptures to 143 controls without ligament/tendon injuries, matched for ancestry, team, age, and injury risk exposure. Results showed that the *COL1A1* G-T haplotype was significantly associated with a reduced risk of ACL rupture, while the TT genotype was less frequent in injured players but not significant. Overall, the G-T haplotype appears protective against ACL injury in professional soccer players.

Beyond *COL1A1*, *MMP3* (rs3025058, 5A/6A), which encodes matrix metalloproteinase-3 (MMP3) involved in connective tissue remodeling, has also been investigated in relation to ACL injury. In a study of professional male football players with ≥2 ACL surgeries compared to controls, no significant association was found between *MMP3* genotypes and ACL injury occurrence, suggesting that *MMP3* alone may not serve as a reliable biomarker, and that risk is likely polygenic and influenced by environmental factors [76,103].

The *COL2A1* gene encodes the alpha-1 chain of type II collagen, which is a crucial component of cartilage, the intervertebral discs, and the vitreous humor of the eye. Type II collagen is a fibrillar collagen primarily found in cartilage and is essential for maintaining the structural integrity and proper function of connective tissues, particularly those that are subjected to mechanical stress. Sun et al. [42] tested the hypothesis that two specific SNPs, rs11784270 (A/C) and rs6577958 (C/T), within the *COL22A1* gene are associated with ACL ruptures in 228 Polish soccer players with ACL ruptures (157 men, 71 women) and 202 controls (117 men, 85 women). No significant associations were found between these variants and non-contact ACL rupture risk.

The *COL5A1* gene, which encodes the collagen alpha-1(V) chain, plays a key role in supporting tissues rich in type I collagen, including muscles, tendons, and ligaments. It is involved in regulating the structure of fibers composed of both type I and type V collagen. Given this role, the study by Lulińska-Kuklik [18] investigated the association between the *COL5A1* rs12722 and rs13946 polymorphisms in 134 male soccer players with ACL ruptures and 211 healthy controls matched for team, age, and injury exposure. Significant genotype differences were found for rs13946 in a dominant model, and the CC haplotype (rs12722/rs13946) was underrepresented in injured players, suggesting a protective effect against ACL rupture. From a gender perspective, Rodas et al. [23] explored the sex-specific ACL rupture risk in relation to collagen gene-related SNPs among 46 FC Barcelona players (24 females) from the 2020–2021 season, examining the association between 108 selected SNPs and a history of non-contact ACL injuries, stratified by sex. Results showed that 29% of females and 4% of males had non-contact ACL injuries. A significant association was found between the rs13946 CC genotype and ACL rupture in females but not in males, with a clear SNP–sex interaction. Notably, the C-allele was exclusive to a specific *COL5A1* haplotype, suggesting that collagen gene-related variants, particularly in *COL5A1*, may increase ACL injury susceptibility in female players. These findings highlight the potential of sex-specific genetic profiling to guide injury prevention strategies [176].

Artells et al. [126] examined the association between the elastin (*ELN*) gene-related SNPs and medial collateral ligament (MCL) injuries in a sample of 60 elite football players monitored over seven seasons. Genotype-specific patterns emerged: players with the *ELN* AA genotype sustained 16 MCL injuries, those with *ELN* AG had 3, while the *ELN* GG genotype was linked to no injuries. The study concluded that identifying *ELN* gene-related polymorphisms could help predict injury risk, guide training and rehabilitation plans, and optimize recovery and workload management to prevent MCL injuries.

Additional extracellular matrix (ECM)-regulating genes also appear relevant but were underrepresented in earlier summaries and genetic panels. Variants in *EMILIN1*, which supports elastin-fiber assembly and tissue remodeling, have been identified in elite injury-mapping cohorts, while *TNC* (Tenascin-C), an ECM glycoprotein responsive to mechanical loading, has been linked to chronic tendinopathy and tendon stiffness profiles in athletes. Genes involved in cytoskeletal dynamics and repair, such as *GEFT*, have emerged in multi-gene injury panels from top-level soccer teams. Similarly, *LIF*, a cytokine essential for muscle regeneration and connective-tissue repair, appears in polygenic injury-prediction frameworks. Finally, *HGF*, a key growth factor governing tissue regeneration and satellite-cell activation, shows polymorphisms associated with injury severity and recovery timelines in both referees and professional players. 

Collectively, these findings highlight that connective tissue injury risk in soccer is highly polygenic, involving structural collagen genes, ECM remodelers, and growth/regeneration pathways. This reinforces the importance of integrating multiple genetic markers when assessing injury susceptibility in elite football environments.

### 3.8. Energy Metabolism-Related Polymorphisms in Soccer

The *AMPD1* gene encodes the adenosine monophosphate deaminase 1 (AMPD1), which plays a role in energy metabolism during exercise [177]. Lifanov et al. [124] assessed the impact of short-term creatine supplementation on exercise performance in male athletes, considering their genetic makeup. Athletes demonstrated a significantly higher frequency of the T allele in *AMPD1* 34T compared to controls. During the experimental phase, 21 football players were randomly assigned to either the creatine group (*n* = 11) or the placebo (dextrose) group (*n* = 10). The *AMPD1* CC genotype displayed the strongest response to creatine, while *AMPD1* CT carriers showed greater gains in relative VO_2_ max and reduced blood lactate accumulation compared to *AMPD1* CC carriers.

The *MCT1* gene encodes the monocarboxylate transporter-1 (MCT1), a key protein in lactate transport and metabolism. By facilitating the movement of lactate and other monocarboxylates (e.g., pyruvate) across muscle cell membranes, *MCT1* helps maintain pH balance and prevents acidosis, thereby supporting muscle function, recovery, and reducing fatigue-related injury risk. Massidda et al. [136] examined the *MCT1* rs1049434 polymorphism in 173 elite Italian footballers, monitoring muscle injuries over five seasons (2009–2014). Indirect muscle injuries included structural-mechanical injuries and functional muscle disorders. The results indicated that players with the *MCT1* AA genotype experienced significantly more injuries compared to those with the TT genotype. The study concluded that the *MCT1* rs1049434 polymorphism is linked to a higher incidence of muscle injuries, even though the statistical significance was nominal [66].

The *UCP1*, *UCP2*, and *UCP3* genes, which encode the uncoupling proteins 1, 2, and 3, are involved in energy regulation and metabolism. Bondareva et al. [178] investigated uncoupling protein (*UCP1–3*) gene polymorphisms in football players compared with non-athletes. A higher frequency of the “energy-sparing” *UCP3* allele was observed, along with similar trends in other *UCP* variants, reflecting the need for energy conservation in athletes. Significant associations were reported between the *UCP1* gene and explosive leg strength, *UCP2* and respiratory function, and *UCP3* with energy production during ramp testing and ergometric efficiency. Moreover, *UCP3* variants were linked to greater uncoupling of oxidation and phosphorylation, increasing total energy consumption while enhancing efficiency and performance near the anaerobic threshold.

Similar trends could be reported for the *FTO* (Fat Mass and Obesity-associated) gene, primarily known for its role in regulating body weight and energy homeostasis. This gene encodes a protein that functions as a DNA/RNA demethylase, influencing the metabolism of fat and, consequently, body composition [179].

Additional genes involved in mitochondrial biogenesis and lipid oxidation were also prominent in soccer cohorts. *PPARA* (rs4253778) regulates fatty-acid metabolism and shows G-allele enrichment in endurance-oriented soccer profiles; *PPARG* (rs1801282) and *PPARD* (rs2016520) contribute to glucose and lipid homeostasis and endurance adaptations. *PPARGC1A* (*PGC-1α*) encoding the transcriptional coactivator PGC-1α, is a master regulator of mitochondrial biogenesis and oxidative metabolism, and its polymorphism Gly482Ser (rs8192678) has been repeatedly associated with aerobic capacity and discrimination between elite and sub-elite players. Finally, *TFAM* (rs1937), essential for mitochondrial DNA replication and transcription, emerged as an important contributor to endurance traits in youth academy cohorts (Table 1).

### 3.9. Oxidative Stress and Detoxification-Related Polymorphisms in Soccer

The *GSTM1* gene encoding the glutathione S-transferase Mu 1 (GSTM1), an enzyme involved in oxidative stress response and detoxification, the *GSTP* gene encoding the glutathione S-transferase pi (GSTP), part of the antioxidant defense system, and the *GSTT* gene encoding the glutathione S-transferase theta (GSTT), another key enzyme in oxidative stress and detoxification pathways, have been linked to professional athlete status in football players [32,125]. These SNPs indeed may influence the athletes’ capacity to cope with oxidative stress and recover from the high-intensity physical demands of the sport. Athletes with favorable variants of these genes may have enhanced resistance to oxidative damage and improved detoxification efficiency, potentially giving them an advantage in terms of recovery, endurance, and performance. 

Additional oxidative stress–related genes have also been identified in soccer cohorts. The *NRF2* (*NFE2L2*) gene, a master transcription factor regulating antioxidant pathways, has been linked to positional demands and inter-individual variability in oxidative stress tolerance [127]. Similarly, *CYP2D6*, a gene involved in xenobiotic metabolism and steroid hormone clearance, appears in elite genetic panels, reflecting its potential role in modulating recovery, metabolic stress, and overall physiological adaptation to training [89].

### 3.10. Hormonal Regulation-Related Polymorphisms in Soccer

The *CYP2D6* gene encodes the cytochrome P450 2D6, part of the larger cytochrome P450 family, which is involved in drug metabolism, and steroid hormone and neurotransmitter regulation. CYP2D6 is responsible for the metabolism of about 20–25% of commonly prescribed drugs, influencing how individuals process medications, making it important for personalized medicine. Variations in the *CYP2D6* gene can lead to different metabolizer types: namely, poor, intermediate, extensive, or ultra-rapid metabolizers, which can significantly affect an individual’s response to medication or other exogenous compounds [180]. It has also been linked to professional athlete status in football players [89]. It can be speculated that the enzyme’s role in hormone regulation may influence physical endurance, recovery, and the body’s ability to adapt to strenuous physical activity. Hormonal balance, including the regulation of cortisol and other stress-related hormones, could play a part in maintaining optimal performance under intense physical and psychological stress. Moreover, genetic variations in *CYP2D6* might influence an athlete’s susceptibility to injury or their ability to metabolize substances such as painkillers or anti-inflammatory drugs, which are commonly used in professional sports settings.

The *HSD17B14* gene encodes the hydroxysteroid 17-beta dehydrogenase 14 (HSD17B14), which is involved in steroid hormone metabolism. It has been associated with sprint test performance in elite youth football players [64]. Sprinting, which demands explosive muscle power and rapid energy mobilization, is, indeed, influenced by the balance of anabolic and catabolic hormones. The ability to rapidly metabolize steroid hormones may give certain athletes a genetic advantage in terms of muscle recovery, energy efficiency, and adaptation to high-intensity training, all of which are critical factors in sprinting performance.

Overall, these genetic factors may help explain why some football players excel in short, intense bursts of activity such as sprints, which are crucial in football for chasing the ball, overtaking opponents, or breaking into open spaces.

### 3.11. Growth Factors and Muscle Hypertrophy-Related Polymorphisms in Soccer

*Myostatin (MSTN)*, a member of the TGF-β (transforming growth factor-beta) family, is a key regulator of muscle growth, and its role in sports performance has attracted attention, particularly in soccer. Polymorphisms or genetic variations in the *MSTN* gene, which encodes myostatin, can affect muscle development, strength, and endurance, traits crucial for athletic performance. Specific SNPs such as K153R, E164K, P198A, and I225T have been identified in the *MSTN* gene [26,181,182].

The gene *IGF2* encodes the insulin-like growth factor 2 (IGF2), involved in muscle hypertrophy and growth. SNPs in these genes have been linked to professional athlete status and increased injury risk [30,53].

The gene *HGF* encodes the hepatocyte growth factor (HGF), which is important in muscle regeneration and repair. Liver metabolism plays, indeed, a crucial role in muscle health by regulating energy production, detoxification, protein synthesis, and fat metabolism. Disruptions in liver function can lead to energy deficits, slower muscle recovery, inflammation, and hormonal imbalances, all of which increase the risk of muscle injuries in footballers. Pruna et al. [53] investigated the role of SNPs in the *HGF* gene to understand interindividual differences in injury severity, recovery time, and injury rate in elite soccer players. The aim was to identify genetic biomarkers that could help prevent or minimize non-contact muscle injuries. Genomic DNA from 74 elite soccer players was analyzed using allelic discrimination techniques. The results showed that SNPs in the *HGF* gene were significantly associated with injury incidence, severity, and recovery time.

### 3.12. Cell Signaling and Gene Expression-Related Polymorphisms in Soccer

The *NRF2* gene encoding the nuclear factor erythroid 2-related factor 2 (NRF2), which regulates oxidative stress response and energy metabolism, has been associated with playing position and performance-related outcomes [127].

The *PPARα* gene, which encodes the peroxisome proliferator-activated receptor alpha (PPARα) and regulates genes involved in fatty acid metabolism and energy homeostasis [144], was studied by Proia et al. [131] to determine the prevalence of the G allele of the *PPARα* intron 7 G/C polymorphism (rs4253778) in professional Italian soccer players. The study involved 60 professional soccer players and 30 sedentary volunteers. The results showed that the G allele and GG genotype were significantly more common in soccer players than in sedentary controls. However, no significant correlations were found between lipid profiles and the genotype. The findings suggest that the G allele and GG genotype, previously linked to endurance athletes, are also prevalent in professional soccer players.

Beyond these signaling pathways, mitochondrial regulators such as *PPARGC1A (PGC-1α)* also contribute to performance-related phenotypes in soccer and are discussed in detail in the energy metabolism section (Section 3.8). *PTPRK* (protein tyrosine phosphatase receptor type K), *SOX15* (SRY-box transcription factor 15) [146], and *GEFT* (guanine nucleotide exchange factor T) have likewise been reported in football cohorts, although their functional roles remain less well characterized [183,184].

### 3.13. Inflammation and Immune Response-Related Polymorphisms in Soccer

Several genetic variants related to inflammation and immune responses have been linked to performance outcomes and injury risk in footballers [118,185,186]. These include the *CCL2* gene, which encodes the C-C motif chemokine ligand 2 (CCL2) involved in immune response and muscle inflammation, the *IL1RN* gene encoding the interleukin-1 receptor antagonist (IL1RN) that regulates inflammation in response to muscle damage, the *IL6* gene encoding interleukin 6 (IL-6), a cytokine central to inflammation and muscle repair following exercise, and the *LIF* gene encoding the leukemia inhibitory factor (LIF), which contributes to muscle repair and regeneration after injury. In this context, Bulgay et al. [77] demonstrated that although *IL-6* (rs1800795) genotypes were not associated with jumping or longer sprint performance (30 m), they were significantly related to training-induced improvements in endurance (Yo-Yo IRT 2), short sprint ability (10 m), and upper-body strength (1RM bench press) after six weeks of training, highlighting the role of *IL-6* genetic variation in shaping adaptive responses to intensive football preparation.

Finally, the 2-month consumption of hydrogen-rich water in female juvenile soccer players was found to significantly enhance antioxidant capacity, reduce oxidative stress (lower malondialdehyde (MDA)) and inflammation (reduced interleukin 1 (IL-1), IL-6, and TNF-α), and improve hemoglobin levels. It also increased gut flora diversity and abundance, suggesting a modulatory effect on the microbiota alongside its antioxidant and anti-inflammatory benefits [125].

### 3.14. Bone Health-Related Polymorphisms in Soccer

The vitamin D receptor (*VDR*) gene influences several musculoskeletal traits, with polymorphisms in this gene previously linked to various pathologies and muscle strength in athletes [150]. Diogenes et al. [118] investigated the role of common *VDR* variants (FokI and TaqI) on longitudinal changes in bone mass and calcium-related hormones among 46 adolescent soccer players, aged 11.8 to 14.2 years. Total body bone mineral content (TBMC) and density (TBMD) were assessed at baseline and after 6 months, while levels of insulin-like growth factor-I (IGF-1), testosterone, intact parathyroid hormone, and plasma bone alkaline phosphatase activity were measured at baseline and after 3 months. At baseline, boys with the Ff genotype exhibited significantly higher TBMC, TBMD, and TBMD Z-scores compared to those with the FF genotype. Additionally, after 3 months, the Ff genotype group showed a significantly greater increase in plasma IGF-1 levels. Although the FokI polymorphism did not influence changes in bone mass after 6 months, the differences observed at baseline persisted. No significant differences were found in bone mass, hormone levels, or other outcome variables based on TaqI genotypes. Flore et al. [112] investigated the relationship between the most commonly studied *VDR* gene variants (rs2228570, rs7975232, and rs1544410) and muscle mass gains in elite young soccer players. A cohort of 55 soccer players aged 15–18 from a professional team was selected for this research. All three polymorphisms were found to be in Hardy–Weinberg equilibrium, with allele frequencies consistent with global population variability. A significant association was identified between rs1544410 polymorphism and increased calf muscle mass. Specifically, individuals carrying the A allele had greater calf muscle mass compared to those with the G allele. Furthermore, haplotype analysis of the two SNPs in linkage disequilibrium (rs7975232 and rs1544410) revealed that the AG haplotype was negatively correlated with calf muscle area [112].

Other *VDR* variants have also been implicated in soccer performance. The ApaI polymorphism was more frequent in elite youth players, with the CC genotype associated with superior speed and explosive power. Conversely, elite youth players were less likely than sub-elite players to carry the BsmI A allele, suggesting a potential disadvantage for high-level performance [128].

Beyond *VDR*, bone metabolism in soccer players has been linked to other genetic pathways. The P2X7R polymorphisms rs1718119 and rs3751143 were associated with baseline cortical traits, such as cross-sectional area (CSA), strength-strain index (SSI), and periosteal circumference, in elite academy players. Although no genotype × training interaction was observed, these SNPs influenced initial bone phenotype differences. Similarly, variants in the *RANK/RANKL/OPG* pathway (rs9594738, rs1021188, rs9594759) were associated with periosteal circumference, cortical CSA, and cortical density, again showing significant baseline differences but no training-induced associations [114].

### 3.15. Overlap of Genetic Pathways Across Performance, Injury, and Metabolic Domains

**Figure 3:**
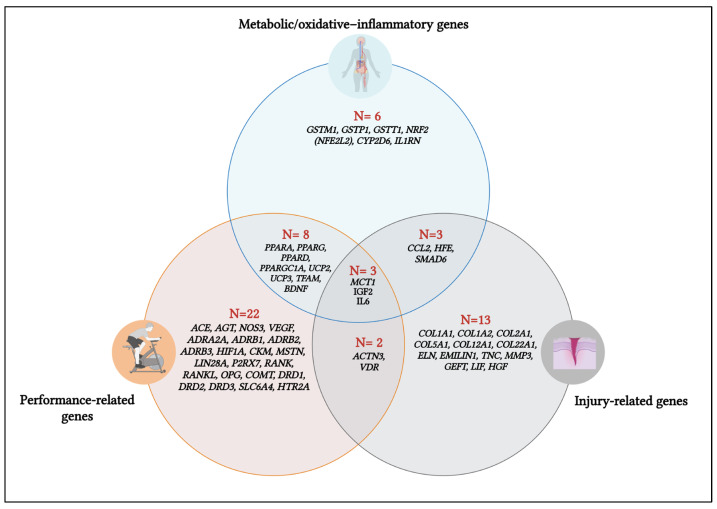
Overlap of performance-related, injury-related, and metabolic/oxidative–inflammatory genes identified in soccer omics research.

The functional classification of genes revealed clear patterns of overlap across performance, injury-related, and metabolic/oxidative–inflammatory domains (Figure 3). A large proportion of variants (*n= 22*) were uniquely associated with performance-related traits, including cardiovascular, neuromuscular, and neurotransmission pathways. Injury-related genes also formed a substantial domain (*n = 13*), primarily involving collagen structure, ECM remodeling, and tissue-repair mechanisms. Metabolic and oxidative–inflammatory genes (*n = 6*) were comparatively fewer and clustered around mitochondrial function, detoxification, and inflammatory regulation. Notably, several genes exhibited cross-domain influence. Eight genes (*PPARA*, *PPARG*, *PPARD*, *PPARGC1A (PGC-1α)*, *UCP2*, *UCP3*, *TFAM*, *BDNF*) overlapped between performance and metabolic functions, indicating the tight coupling between bioenergetics and athletic output. Three genes (*CCL2*, *HFE*, *SMAD6*) intersected metabolic and injury-related pathways, reflecting the shared role of inflammation and remodeling in both energy homeostasis and tissue vulnerability. Two genes (*ACTN3*, *VDR*) overlapped performance and injury domains, highlighting their dual involvement in muscle function and structural integrity. Importantly, only three genes—*MCT1*, *IGF2*, and *IL6*—were present across all three domains, underscoring their central, multi-system contribution to performance, recovery, and metabolic adaptation in soccer players.

### 3.16. Shifting from Single Nucleotide Polymorphisms to Polygenic Approaches

Shifting from SNPs to polygenic approaches, as a recent trend in soccer, reflects a move toward a more holistic understanding of genetic contributions to athletic performance. While SNPs focus on individual genetic variations, polygenic approaches aggregate multiple variants to provide a broader, more accurate prediction of traits like endurance, speed, and injury risk, enabling personalized training and talent identification.

Petr et al. [90] analyzed the impact of several genetic variants on performance in 99 elite male soccer players compared with 107 controls, including positional differences. Players underwent isokinetic quadriceps and hamstring strength testing (60°/s, 180°/s, 300°/s) and jump assessments. The study focused on *ACTN3* (R577X, rs1815739), *ACE* (I/D, rs1799752), *NOS3* (Glu298Asp, rs1799983), *AMPD1* (34C/T, rs17602729), *UCP2* (Ala55Val, rs660339), *BDKRB2* (+9/−9, rs5810761), and *IL1RN* (VNTR 86 bp). Defenders with the *ACTN3* XX genotype had lower quadriceps and hamstring strength at all speeds, while RR carriers showed the highest strength compared to RX heterozygotes. Additional associations linked *AMPD1* CC, *NOS3* Glu/Glu, and IL1RN*2 alleles with greater lower limb strength, particularly in attackers and defenders. A total genetic score explained 26% of the variance in jump and strength performance. Overall, the *ACTN3* R allele, *NOS3* Glu/Glu genotype, and IL1RN*2 allele predisposed attackers and defenders to superior strength and power, whereas midfielders showed lower values independent of these variants.

Egorova et al. [36] investigated the association of common gene polymorphisms with football player status, individually and in combination. A total of 246 Russian football players and 872 controls were genotyped for eight gene polymorphisms known to be associated with athletic status. Four specific alleles (*ACE* D, *ACTN3* Arg577, *PPARA* rs4253778 *C*, and *UCP2* 55Val) were identified as having discrete associations with football player status. Football players had a significantly higher mean total genotype score compared to controls, indicating that individuals with a higher number of “favorable” gene variants have an increased likelihood of becoming football players.

Contrò et al. [127] proposed an innovative genetic framework to assess performance-enhancing polymorphisms (PEPs) in five genes—*PPARα*, *PPARGC1A (PGC-1α)* Gly482Ser (rs8192678), *NRF2*, *ACE*, and *CKMM*—to distinguish elite soccer players from non-athletes. The study included 60 professional players and 60 controls. Significant associations were found for the *NRF2* AG/GG genotype and, more strongly, for the *ACE* polymorphism, particularly the ID and II genotypes, which were closely linked to the soccer player phenotype. While other PEPs showed no direct significance, *PPARα* appeared to amplify the effect of *NRF2* (GG), suggesting potential gene–gene interactions contributing to performance.

To account for these interplays of effects [81], Maestro et al. [120] calculated a total genotype score to quantify the combined influence of six polymorphisms (*AMPD1*, *ACE*, *ACTN3*, *CKM*, and two *MLCK* variants) on injury risk. A total of 122 male professional football players were recruited and analyzed for these polymorphisms using SNP Extension (SNPE). A score of 2 was assigned to “protective” genotypes, 1 to heterozygous genotypes, and 0 to the “worst” genotypes for injuries. Key findings included significant differences in the distribution of allelic frequencies of *AMPD1* and *MLCK* polymorphisms between non-injured and injured players. The average total genotype score for non-injured players was higher than for injured players.

This approach has also been leveraged by Massidda et al. [122], who recruited sixty-four male top-level football players. These were genotyped for four specific gene polymorphisms: *ACE* I/D (rs4341), *ACTN3* c.1729C > T (rs1815739), *COL5A1* C > T (rs2722), and *MCT1* c.1470A > T (rs1049434). Muscle injuries over a ten-year period were analyzed, with the results indicating that the distribution of certain polymorphisms (*ACE* I/D, *ACTN3*, *MCT1*) differed significantly between injured and non-injured players. A higher presence of “protective” gene variants correlated with a lower incidence of muscle injuries. Non-injured players had a higher total genotype score compared to injured players.

Similarly, McAuley et al. [121] explored the relationship between 22 SNPs and key athletic traits such as acceleration, change in direction, jump height, and speed in 149 male academy football players, aged under-12 to under-23, from four English academies. Players completed sprint, countermovement jump, and agility tests. *GALNT13* (rs10196189) G allele was linked to ~4% faster sprints, while the *IL6* (rs1800795) G/G genotype was associated with ~16% higher jump performance. Combined genotype scores correlated strongly with all performance traits, explaining 6–33% of the variance.

While a few studies using the total genotype score have been published [187], there is currently only a single genome-wide association study (GWAS) [115], which was conducted to identify genetic variants linked to sprint performance in elite youth football players. Using microarray data, the researchers analyzed 1206 subjects, identifying 12 SNPs with suggestive significance after replication. The study also validated some of the discovered SNPs in additional cohorts.

### 3.17. Current Limitations of Genomics in Soccer Research and Future Directions

Genomic testing offers a powerful means to tailor training programs to an athlete’s genetic predispositions, optimizing performance while reducing injury risks, as PEPs are subject to selective pressures [115,154]. However, current research in soccer often lacks sufficient ethnic diversity and fails to provide a detailed analysis of players’ field positions. Additionally, the role of psychological traits in football performance remains underexplored. To address these gaps, future studies should adopt more robust research designs, increase sample sizes, and integrate advanced genetic methodologies such as GWAS and polygenic profiling.

In response to these limitations, the Football Gene Project was recently introduced. This initiative seeks to foster individualized athlete development by incorporating genetic, psychological, and positional factors into training protocols [154,155]. A deeper understanding of how polymorphisms influence athletic performance requires examining the complex interactions between genetic variations, which may be crucial to unlocking an individual’s full genetic potential for elite performance.

Traditional genetic association studies in soccer are often limited by inconsistent findings across populations, reflecting differences in genetic background, environment, and physiological traits. These discrepancies underscore the need for replication and validation—particularly because the vast majority of existing associations in soccer derive from candidate-gene studies rather than genome-wide approaches—before any genetic markers can be applied reliably in sports science. For instance, Kanope et al. [96] conducted a groundbreaking study by fully sequencing the genomes of 44 male Brazilian first-division under-20 soccer players (U20_BFDSC) and comparing them to global population databases (1000 Genomes). Using genetic distance and molecular variance analyses, they found these players were more genetically differentiated from African populations than expected, despite prior evidence of 12–24% similarity between Brazilians and Africans. Instead, the U20_BFDSC players clustered closer to professional athletes, suggesting that genetic selection linked to performance may occur before full maturation. This study and a similar one [187] show that performance-related genes are likely influenced by a combination of physical, environmental, cognitive, and sociocultural factors. Cutting-edge molecular variance analysis and Wright’s statistics can offer valuable insights into performance-related genetic differences in soccer science.

## 4. Proteomics in Soccer

Proteomics, the study of the entire set of proteins expressed in a cell or organism, is particularly useful for understanding muscle physiology and recovery. Proteins are central to muscle repair and inflammation, and analyzing their expression can provide insights into how well an athlete is recovering from strenuous exercise or injury [188].

Currently, there exist only a few studies applying proteomics in soccer science. Martín-Sánchez et al. [25] aimed to investigate whether an intensive pre-season training program affects the inflammatory status of professional soccer players and how this may be linked to their physical condition. The researchers compared plasma protein biomarkers, cardiac function, and physiological state between 12 professional and 9 recreational soccer players. Following the training, professional players exhibited reduced cardiac low frequency compared to recreational players, though no significant differences were observed in other cardiac measures (e.g., high frequency, oxygen consumption). Proteomic analysis revealed that certain inflammatory and oxidative stress-related proteins, such as alpha-1-antitrypsin isotype-3 and fibrinogen-gamma, were reduced in professional players, while others, like alpha-1-antitrypsin isotype-6 and alpha-1-antichymotrypsin increased. Spearman’s correlation linked cardiac low frequency positively with fibrinogen-gamma isotype-3 and negatively with alpha-1-antichymotrypsin isotype-4. Findings suggest pre-season training modulates plasma proteins involved in inflammation, oxidative stress, and thrombosis in professionals.

### Current Limitations of Proteomics in Soccer Research and Future Directions

Despite its potential, proteomics in soccer science faces several limitations. One major challenge is the complexity of protein expression, which can vary greatly depending on factors like training load, injury status, diet, and individual physiology. The high cost and technical expertise required for proteomic analysis limit its widespread use in soccer research. Moreover, standardizing sample collection, processing, and analysis is difficult due to the variability in protein expression across different biological matrices (e.g., blood, muscle tissue, or urine). This variability makes it challenging to draw definitive conclusions and apply findings universally. Future directions should focus on expanding the sample sizes in studies, integrating proteomics with other “omics” approaches (like metabolomics and genomics) to gain a more holistic view of an athlete’s health and recovery, and developing cost-effective, accessible technologies for routine monitoring in sports settings. Collaborations between sports scientists, bioinformaticians, and clinicians will be key to translating proteomics research into practical applications for enhancing performance and recovery in soccer.

## 5. Metabolomics in Soccer

Metabolomics, which involves the study of small molecules (metabolites) within cells, tissues, or organisms, offers valuable insights into energy metabolism and recovery processes in athletes. The ability to track metabolite fluctuations provides real-time feedback on an athlete’s physiological state, which can be used to adjust training intensity or nutritional interventions [59,72,85,100,101,189].

Książek et al. [20] investigated novel markers of vitamin D status, including free 25-(OH)D, bioavailable 25-(OH)D, and the vitamin D metabolite ratio (VMR), alongside psychophysical stress markers during different training periods over half a season in professional football players. Twenty athletes were tested at six time points over six months to assess seasonal variations in vitamin D binding protein (VDBP), total and free 25-(OH)D, and other vitamin D metabolites. Significant seasonal rhythms were observed for VDBP, total and bioavailable 25-(OH)D, and several metabolites, but not for free 25-(OH)D or stress markers (ferritin, liver enzymes, CK, testosterone (T), cortisol (C), T/C ratio). No associations emerged between vitamin D status and stress markers. A strong correlation persisted between 25-(OH)D3 and 24,25-(OH)2D3, and training load did not alter resting vitamin D metabolite concentrations. Overall, free 25-(OH)D was not superior to total 25-(OH)D in reflecting vitamin D status relative to stress. Ra and colleagues [38] used metabolomics to identify salivary fatigue markers in soccer players after three consecutive days of games. The study involved 122 male soccer players, and traditional fatigue symptoms such as heart rate, body mass, and mood were measured before and after the program. Out of the participants, 37 players showed signs of fatigue. Saliva samples from these fatigued players were analyzed using capillary electrophoresis and time-of-flight MS, with data processed using principal component analysis. Metabolomics identified 144 metabolites in the saliva of fatigued players, showing significant metabolic changes before and after the three-day game program. All metabolites were increased post-program, with notable metabolites like 3-methylhistidine, glucose 1- and 6-phosphate, taurine, and several amino acids involved in muscle breakdown, glucose metabolism, lipid metabolism, amino acid metabolism, and energy metabolism.

Another example of metabolomics in soccer is the study by Gouveia et al. [113], which used metabolomic profiling to monitor changes in the urinary metabolites of elite female soccer players throughout a championship season. The study identified several metabolites linked to energy and protein metabolism, including glycine, citrate, and urea, that showed significant variation pre- and post-match.

Kim et al. [87] analyzed urinary metabolites in young Korean soccer players after 1, 5, and 10 days of winter training season using NMR spectroscopy and multivariate analysis to recommend optimal recovery times for improving performance. A total of 79 metabolites were identified from urine samples, with 15 metabolites—such as 1-methylnicotinamide, 3-indoxylsulfate, galactarate, glutamate, and lactate—showing significant changes after winter training season. These metabolites are involved in key metabolic processes, including the urea, purine nucleotide, and glucose-alanine cycles. Most metabolites spiked after 1 day of the winter training season and returned to normal levels. However, four metabolites—adenine, 2-hydroxybutyrate, alanine, and lactate—remained elevated for 5 days post-training, suggesting that at least 5 days of recovery are needed based on excess ammonia, adenine, and lactate levels.

Similarly, Pintus et al. [139] conducted a ^1^H-NMR analysis of urine samples from 21 professional soccer players collected at three time points during the preseason preparation for the Italian Serie A Championship. The study revealed that the urinary metabolite profile changed over the observational period. Notably, significant variations were observed in the levels of trimethylamine-*N*-oxide, dimethylamine, hippuric acid, hypoxanthine, guanidoacetic acid, 3-hydroxybutyric acid, citric acid, and creatine. These changes were likely influenced by factors such as diet, training regimen, and gut microbiota. For example, trimethylamine-*N*-oxide and hippuric acid, both of dietary origin, are also linked to microbiota activity, while 3-hydroxybutyric acid is associated with the type of physical exercise.

França et al. [17] investigated the impact of soccer exercise on tyrosine metabolism using an untargeted, sportomics-based analysis of urine samples from 30 male junior professional soccer players. Samples were collected before and after a match and analyzed via liquid chromatography and MS. The results showed significant changes in tyrosine metabolism, including a downregulation of homogentisate metabolites (4-maleylacetoacetate and succinylacetone) by 80% and 84%, respectively, and an upregulation of 4-hydroxyphenylpyruvate by 26%. Hawkinsin and its metabolite 4-hydroxycyclohexyl acetate increased six-fold, while DOPA and dopaquinone levels rose four- to six-fold. Conversely, 3-methoxytyrosine, indole-5,6-quinone, melanin, dopamine, and tyramine decreased by up to 99%. Blood total carbon dioxide (TCO_2_) and urinary glutathione and glutamate decreased, with a two-fold increase in pyroglutamate. The metabolic changes observed bore unexpected similarities to Hawkinsinuria, suggesting a transient condition termed exercise-induced hawkinsinuria, indicating that soccer exercise could serve as a model to explore potential treatments for Hawkinsinuria and other disorders affecting tyrosine metabolism.

Finally, Peña et al. [15] applied lipidomics to assess erythrocyte membrane lipid profiles in 40 professional female footballers from Athletic Club Bilbao over three seasons (2019–2022), generating 160 samples. Compared with the general population, players exhibited lower di-homo-γ-linolenic acid (DGLA) but elevated arachidonic acid (AA), eicosapentaenoic acid (EPA), and saturated fatty acid (SFA)/monounsaturated fatty acid (MUFA) ratios. Seasonal patterns showed an early rise in docosahexaenoic acid (DHA) and total polyunsaturated fatty acid (PUFA), followed by further AA and PUFA accumulation during periods of intense training. Weak negative correlations were noted between selected fatty acids and vitamin D, urea, cortisol, and age. The study concludes that competitive seasons drive marked shifts in erythrocyte fatty acid composition, particularly PUFA increases and DGLA reductions, with potential consequences for immune regulation and anti-inflammatory responses.

### Current Limitations of Metabolomics and Lipidomics in Soccer Research and Future Directions

Overall, these findings suggest that metabolomic data can be instrumental in understanding fatigue, energy expenditure, and recovery needs, while lipidomics could be a valuable tool for developing personalized nutritional strategies for elite football players to address metabolic imbalances during the season. On the other hand, metabolomics studies present some challenges, particularly in the analysis of saliva samples from athletes. One major issue is the variability in data normalization methods, such as whether to use total protein or total metabolite concentrations, which can significantly affect the interpretation of results. Additionally, saliva as a biofluid poses its own limitations—systemic responses to exercise are short-lived in saliva compared to blood, making it difficult to capture lasting metabolic changes. The heterogeneous nature of soccer players’ activities during a match and the timing of sample collection further complicate the ability to draw consistent conclusions about metabolic shifts. These challenges are compounded by the fluctuating water content in saliva, which demands precise normalization to accurately reflect metabolic changes. The variation in physical exertion among players also affects the consistency of measurable metabolic markers. Despite these obstacles, overall findings suggest that metabolomics data can be instrumental in understanding fatigue, energy expenditure, and recovery needs in athletes, provided that the methodological complexities are carefully managed [61].

## 6. Microbiomics in Soccer

The gut microbiome, composed of trillions of microorganisms residing in the digestive tract, plays a critical role in maintaining overall health, immune function, and even athletic performance. Research has increasingly shown that athletes with a healthier, more diverse gut microbiota recover faster and perform better over time [19,190].

However, there exist a few studies that report gut microbiota parameters in elite soccer players and provide insights into how varying physical activity levels affect gut microbiota composition. Petri et al. [19] investigated the relationship between physical activity levels and gut microbiota composition by comparing four groups of healthy young males: 17 elite soccer players, 14 individuals with high physical training, 23 with moderate physical activity, and 37 sedentary men. Key findings included a significantly higher prevalence of nine microbiota populations in elite soccer players and highly active individuals compared to those with moderate or no physical activity. However, there were no differences in the *Firmicutes* to *Bacteroidetes* ratio among the groups.

Urban et al. [14] analyzed 20 professional football players and 12 amateurs. The results showed that oral microbiota diversity was similar between both groups, though increased training intensity led to a reduction in bacterial species. However, the gut microbiota revealed a significant difference between professionals and amateurs, especially during intensive training. *Firmicutes* dominated the microbial population across all groups. Intensive physical activity was associated with an increase in butyrate- and succinate-producing bacteria, which are beneficial for maintaining metabolic homeostasis and supporting immune system function, underscoring the positive impact of exercise on gut microbiota and overall health.

Kenger et al. [140] investigated the relationship between microbiota profiles and the nutritional status of professional football players who perform endurance exercises. Twenty male professional footballers from a Turkish Football Federation Second League club participated in the study. Fecal samples were collected and analyzed through 16s rRNA gene sequencing, and the players’ body composition was measured using a bioelectrical impedance analyzer. The participants’ 3-day food intake was recorded with the assistance of a dietitian. The analysis identified four *phyla*, 10 *genera*, and four *species* in the microbiota with densities exceeding 1%. A negative correlation was observed between body fat percentage and the *species Faecalibacterium prausnitzii*, *Bacteroides vulgatus*, and the *genus Faecalibacterium*. In terms of nutrition, fat intake was positively correlated with *Actinobacteria* and *Blautia coccoides*; energy and fiber intake were correlated with *Prevotella* and *Prevotella copri*. Additionally, carbohydrate intake was negatively correlated with *Faecalibacterium*.

Given the link between microbiota composition and nutritional intake, uncovered in the previous study, microbiomics research can be leveraged to devise personalized nutritional strategies aimed at enhancing and optimizing footballers’ performance. For instance, Mancin et al. [16] investigated the effects of consuming 30 g of dark chocolate daily for 4 weeks on blood lipid profiles and gut microbiota composition in elite male soccer players. The participants were randomly assigned to either a dark chocolate group or a white chocolate control group. Blood, fecal samples, and anthropometric data were collected at baseline and after the intervention. The dark chocolate group showed significant improvements in blood lipid profiles, including reductions in total cholesterol, triglycerides, and low-density lipoprotein, along with an increase in high-density lipoprotein. The ratio of AA to EPA in the blood decreased significantly in the dark chocolate group compared to the white chocolate group, indicating a potential reduction in inflammation. Plasma polyphenol levels increased in the dark chocolate group. Further, gut microbiota in the same group showed slightly higher stability over time, with lower community dissimilarity.

In another study, the same group [21] aimed to assess the influence of a ketogenic Mediterranean diet with phytoextracts (KEMEPHY) on gut microbiome composition in semi-professional soccer players. Sixteen male players were randomly assigned to either a KEMEPHY diet group (*n* = 8) or a Western diet group (*n* = 8). The researchers measured body composition, performance, and gut microbiome composition before and after 30 days using 16S rRNA amplicon sequencing. Alpha diversity measures and PERMANOVA were employed to investigate changes in microbial abundance at various taxonomic levels, and correlations between microbial composition and macronutrient intake were assessed. The results showed no significant pre- and post-intervention differences in microbial diversity. However, a significant time-group effect was found for the *Actinobacteriota phylum*, which increased in the Western diet group and decreased in the KEMEPHY diet group. Linear discriminant analysis identified specific *genera* that distinguished the two diets: *Bifidobacterium*, *Butyricicoccus*, and *Acidaminococcus* were more abundant in the Western diet group, while *Clostridia* UCG-014, *Butyricimonas*, *Odoribacter*, and *Ruminococcus* were more abundant in the KEMEPHY group.

### Current Limitations of Microbiomics in Soccer Research and Future Directions

Microbiomics research in soccer highlights the potential role of gut and oral microbiota in shaping performance, recovery, and health, but significant limitations remain. Current studies are constrained by small cohorts, short durations, and methodological inconsistencies in sequencing and analysis, reducing reproducibility and generalizability. The complex interactions between diet, training load, genetics, and environmental factors are rarely disentangled, and most research has been limited to male athletes, leaving female players underrepresented. To advance the field, future studies should adopt larger, longitudinal, and more diverse designs to track microbiota dynamics across training cycles, competitive seasons, and injury recovery. Integrating multi-omics approaches (including metabolomics and proteomics) could help uncover mechanistic pathways linking microbiota to biochemical/physiological performance. Moreover, personalized nutrition strategies tailored to microbiota profiles show promise for optimizing player health and recovery, though robust clinical validation is required before widespread application in elite soccer.

## 7. Integration of Multi-Omics Data in Soccer

The true strength of Socceromics lies in the integration of multiple omics datasets, which together provide a comprehensive view of an athlete’s biochemical/physiological state. By combining genomics, metabolomics, proteomics, and microbiomics, clubs and sports practitioners can gain insights into how players are likely to perform under different conditions, how they recover from injuries, and what specific interventions are most effective for their health (Table 2). Metabolic and physiological/biochemical responses in footballers are, indeed, multifactorial, driven by a combination of dietary intake, physical training, and microbiome interactions [123].

For instance, González et al. [123] explored how genetic data, when integrated with metabolomic and workload data, could predict injuries in elite female footballers. The study identified specific genetic polymorphisms associated with increased risk of muscle and ligament injuries. This kind of research is laying the groundwork for precision medicine in sports, where interventions can be customized based on an athlete’s genetic and metabolic profile.

Orrù et al. [130] conducted a study comparing nine lifelong football players (average age 67.3 ± 2.8 years) with nine age-matched untrained individuals. Using proteomics and metabolomics analysis of *Vastus lateralis* muscle biopsies, the data were processed through various bioinformatic tools. The study showed that lifelong football training enhances muscle oxidative capacity, favoring fatty acid utilization as an energy source and supporting healthier body composition and metabolic profiles. Lifelong players also exhibited elevated muscle polyamine levels, linked to growth and hypertrophy. These findings suggest that continuous football training benefits proteins and metabolites involved in oxidative metabolism and muscle development, thereby promoting healthier aging.

### Current Limitations of Multi-Omics in Soccer Research and Future Directions: Toward Socceromics

The integration of multi-omics data in soccer offers great potential but faces several barriers. Generating and analyzing large datasets remains costly and complex, with variations in sampling, handling, and analytical platforms limiting reproducibility and comparability. A further challenge is the lack of standardized frameworks for interpreting and translating omics findings into actionable strategies for performance, recovery, and injury prevention. Most studies are conducted in controlled settings, which may not reflect the dynamic realities of soccer, and the focus on male athletes leaves female players underrepresented. Future progress will require standardized protocols, more diverse cohorts, and practical tools for coaches and medical teams to apply omics insights in real-world settings. Longitudinal and real-time monitoring approaches could enhance predictive accuracy and support more personalized interventions, advancing the promise of Socceromics [191].

## 8. Quality Assessment Results

Across the included studies, the overall methodological quality ranged from low to high, with the majority of studies classified as moderate quality. Several recurrent limitations contributed to the moderate or low ratings. First, small sample sizes were a frequent issue, with many studies including fewer than 30 athletes and, in some cases, relying on highly specific subgroups (e.g., professional squads, elite youth players), thereby limiting generalizability. Second, a substantial proportion of studies used cross-sectional designs, which restrict causal inference and increase vulnerability to confounding. Third, lack of control groups or use of convenience controls often reduced the internal validity of genetic and molecular associations. Additionally, heterogeneity in analytical protocols, particularly in proteomics workflows, biochemical assays, and genotyping methods, contributed to variability in quality ratings. Finally, several studies provided limited reporting on blinding, randomization, or adjustment for key covariates such as training load, ethnicity, or injury history, which further weakened methodological robustness. Despite these limitations, a subset of studies demonstrated high methodological rigor, particularly those using well-controlled laboratory procedures, standardized performance tests, and clearly described genotyping pipelines. More detailed information on the quality assessment of each study is provided in Table S2 of the Supplementary Materials.

## 9. Discussion

This study highlights the critical role that omics technologies—genomics, metabolomics, proteomics, and microbiomics—play in enhancing performance, injury prevention, and recovery in elite soccer players. By integrating these approaches, Socceromics aims to provide a comprehensive understanding of how various biochemical/physiological and molecular profiles influence player outcomes. This integrative perspective is particularly relevant in a sport characterized by repeated high-intensity efforts, complex neuromuscular demands, and substantial inter-individual variability in training responsiveness.

The findings of these studies reviewed here support the growing body of evidence that personalized, data-driven interventions can significantly improve player performance, health, and recovery, offering clubs a strategic advantage in tailoring training programs and recovery protocols. The genomic analysis revealed several candidate-gene–based associations—such as *ACE*, *ACTN3*, and *COL1A1*—with key traits including endurance, speed, muscle injury risk, and recovery efficiency [45,60,152]. The association of *ACE* polymorphisms with performance and hypertrophy confirms previous findings in the literature, where the I/D polymorphism has been linked to both endurance and strength traits [79]. Moreover, our results corroborate prior studies that found *ACTN3* genotypes to play a pivotal role in speed and injury susceptibility, emphasizing the utility of genomic testing for injury prevention and athlete management [88]. Additional *loci* identified—such as *MCT1*, *AMPD1*, *IL6*, and *HGF*—reinforce the notion that athletic phenotypes in soccer arise from polygenic interactions rather than single genetic determinants. These findings collectively demonstrate that genomic variability maps onto biologically meaningful pathways that influence muscle architecture, metabolic efficiency, neuromuscular transmission, and tissue remodeling.

Metabolomics analysis provides insights into the real-time biochemical/physiological responses of soccer players during and after intense physical activity [113,125,130,139,148]. The findings of significant changes in metabolites linked to energy metabolism, protein breakdown, and recovery processes align with prior research, which shows that monitoring metabolic fluctuations can optimize training loads and recovery strategies. The identification of fatigue-related metabolites, such as 3-methylhistidine and amino acids, suggests that metabolomics could be a powerful tool for managing player fatigue and preventing overtraining. However, as with many metabolomics studies, variability in sample normalization and the transient nature of certain metabolites may pose challenges for broader application, particularly in a sport as physically variable as soccer [113,125,130,139,148]. Despite these limitations, metabolomics remains uniquely positioned to capture the acute biochemical/physiological consequences of high-intensity intermittent exercise, providing a dynamic complement to static genomic markers.

Proteomics analysis provided further validation of muscle physiology and recovery processes, identifying key proteins involved in inflammation and muscle repair [25,130,147]. These findings emphasize the importance of monitoring protein biomarkers to assess recovery times and identify potential injury risks. Proteomics can thus be a valuable tool in predicting player readiness, enabling clubs to adjust training loads accordingly and avoid muscle strain injuries, a common issue in professional soccer. Importantly, alterations in fibrinogen isoforms, α-1-antitrypsin variants, and markers of oxidative stress reflect not only tissue damage but also the activation of adaptive remodeling processes that contribute to long-term performance optimization. This highlights proteomics as a sensitive tool for detecting subclinical fatigue or maladaptive training responses.

The role of microbiomics in soccer, though a relatively new area of research, offers promising avenues for performance enhancement and injury prevention [86,125,140]. Our review included studies that found significant associations between gut microbiota composition and both physical performance and recovery, underscoring the impact of diet and microbiome health on overall player fitness. These findings are consistent with emerging research that shows a diverse gut microbiota is linked to improved metabolic health and reduced inflammation in athletes. The observed shifts in butyrate-producing and succinate-producing *taxa* during high training loads further suggest that microbial metabolites may modulate immune readiness, metabolic flexibility, and even cognitive resilience—factors increasingly recognized as crucial in elite team sports. Personalized nutrition strategies based on microbiomics profiles could therefore become an essential component of athlete care, optimizing both performance and recovery.

Beyond findings at the level of individual omics layers, the collective evidence reviewed here underscores several converging biological themes that align with systems-biology and precision-training frameworks. Many of the identified genetic variants, proteins, metabolites, and microbiome-derived signals converge on a limited set of core pathways, including mitochondrial biogenesis (e.g., *PPARGC1A*; lactate- and amino-acid–related metabolites), chronic and acute inflammation (e.g., *IL6*; *CRP*; microbial diversity), collagen turnover and connective tissue remodeling (e.g., *COL1A1*, *COL5A1*; *MMP*-related proteins), and neuromuscular signaling (e.g., *ACTN3*; dopaminergic and serotonergic markers). The integration of these pathways provides a mechanistic explanation for inter-individual variability in training responsiveness, recovery kinetics, and injury susceptibility. In this sense, omics datasets do not operate at the level of isolated biomarkers; instead, they represent multilayer molecular networks in which individual biomarkers are embedded as interconnected nodes, within larger regulatory networks that can inform precision training load management, injury-risk stratification, and individualized recovery strategies. To further situate these findings within an integrative conceptual model, our synthesis also aligns with a systems-biology perspective and a precision-training approach. From this standpoint, genomic variants represent stable predispositions, while metabolomics, proteomics, and microbiomics profiles capture dynamic biochemical/physiological states shaped by training load, nutrition, recovery, and environmental stressors. Mapping these multilayer interactions onto fundamental pathways—such as mitochondrial function, inflammatory modulation, tissue remodeling, and neuromuscular coordination—provides a mechanistic rationale for individualized training prescriptions. This systems-level interpretation supports precision-training strategies in which omics-derived fingerprints guide personalized load management, recovery optimization, and injury-prevention protocols adapted to each athlete’s unique biological profile.

Moreover, because many of these pathways interact in nonlinear and multi-layered ways, future research would benefit from adopting network-based modeling approaches—including pathway-enrichment analysis, gene–gene interaction networks, and multilayer omics integration frameworks—to more accurately capture convergence and divergence across datasets. Such approaches would allow researchers to move beyond correlation-based interpretations toward causal and mechanistic modeling, strengthening the translational value of omics in applied sport sciences. Likewise, integrating advanced computational approaches such as machine learning and AI-driven prediction models could dramatically strengthen precision-training applications by identifying complex, emergent patterns across omics layers that cannot be detected through single-modality or linear analytical strategies. The integration of multi-omics data in the studies here reviewed illustrates the complexity of athletic performance and the importance of a holistic approach to training and recovery. By combining genomics, metabolomics, proteomics, and microbiomics data, we can create a more detailed picture of a player’s physiological state, which can be used to tailor interventions for maximal performance and injury prevention. Such integration also opens the possibility of generating individualized “omics fingerprints” capable of predicting training responsiveness, susceptibility to soft-tissue injury, and optimal recovery timelines. The predictive power of these integrated datasets suggests that future advancements in Socceromics will focus on even more precise interventions, customized to the genetic and metabolic profiles of individual players. Ultimately, the convergence of omics technologies with advanced analytics holds the potential to transform football from experience-driven practice into a scientifically optimized discipline grounded in objective, personalized biological insights.

### 9.1. Limitations and Future Prospects

Despite these promising results, this review has several limitations. First, although the integration of genomics, proteomics, metabolomics, and microbiomics provides a broad overview of athlete physiology and biochemistry, the included studies displayed substantial methodological heterogeneity, particularly regarding sample preparation, sequencing technologies, analytical pipelines, and statistical thresholds. Such variability limits the direct comparability of findings across omics domains and may contribute to inconsistent or non-replicable associations. Second, sample sizes were frequently small, especially in genomics and microbiomics studies, reducing statistical power and increasing the risk of false-positive or underpowered results. Larger, multi-center cohorts are necessary to confirm the biological relevance of candidate variants and metabolic signatures. Third, the existing literature is characterized by an overrepresentation of European male professional players, with very limited inclusion of women, youth categories, or non-European populations. This demographic imbalance restricts the generalizability of findings and prevents meaningful exploration of sex- and ethnicity-specific omics profiles. Fourth, although we conducted a structured quality assessment, the overall heterogeneity of study design, including observational vs. interventional approaches, acute vs. chronic assessments, and variability in sample timing, limits the strength of cross-study synthesis. In particular, differences in analytical platforms (e.g., SNP arrays, whole-genome sequencing, MS-based proteomics, NMR vs. MS metabolomics) hinder the ability to draw unified mechanistic conclusions. Fifth, most studies relied on cross-sectional designs, which do not capture the dynamic nature of molecular adaptation to training, fatigue, injury, or recovery. Longitudinal and repeated-measure omics studies across full competitive seasons are needed to understand temporal trajectories and intra-individual variability.

Moreover, as previously stated, there was a marked sex imbalance, with a substantial overrepresentation of studies conducted in male football players. The large majority of genetic, metabolomics, and multi-omics investigations focused exclusively on men, while only a limited number of studies examined female athletes or provided sex-stratified analyses. This disparity reflects a persistent gap in sports genomics research and limits the generalizability of current findings to women. Given the known sex differences in physiology, injury epidemiology, hormone regulation, and training adaptations, the underrepresentation of female cohorts highlights the need for more sex-inclusive omics research in soccer.

Finally, the reviewed studies rarely incorporated psychological or neurocognitive factors, despite growing evidence that neurobiological pathways (e.g., dopamine, serotonin, BDNF) influence tactical performance, motivation, and fatigue perception. Integrating behavioral and cognitive markers with multi-omics data would allow a more complete systems-biology perspective.

Overall, while this review supports the potential of Socceromics to optimize training, recovery, and injury prevention, future research should address these limitations by employing larger, more diverse cohorts, standardized analytical workflows, and longitudinal designs. The integration of machine learning and AI will further enhance the predictive value of multi-omics approaches and accelerate their translation into precision performance strategies.

### 9.2. Future Directions: CRISPR/Cas9 and Gene Editing in Sports Genomics

Looking ahead, emerging gene-editing technologies such as CRISPR/Cas9 present both intriguing opportunities and substantial ethical challenges in the context of sports genomics [192]. From a theoretical standpoint, CRISPR-based approaches could one day enable the targeted correction of genetic variants associated with injury susceptibility (e.g., connective tissue or muscle-repair genes), modulation of recovery-related pathways, or the enhancement of mitochondrial function through regulators such as *PPARGC1A* (*PGC-1α*) or *NRF2.* Such interventions could potentially transform long-term player health management, particularly for athletes with clearly detrimental risk alleles [192].

However, the application of gene editing in sport raises profound ethical, regulatory, and fairness considerations. The World Anti-Doping Agency (WADA) currently prohibits all forms of gene editing for performance enhancement, classifying "gene doping" as a banned category [193]. Beyond regulatory constraints, concerns related to safety, off-target effects, heritability, and the reinforcement of genetic inequalities pose significant barriers to any practical use of CRISPR in athletic contexts [192]. Therefore, while CRISPR/Cas9 offers a compelling lens for envisioning the future of precision sports medicine, its application in elite sport must remain confined to therapeutic, non-enhancement purposes and governed by strict ethical oversight.

As sports genomics continues to evolve, responsible integration of advanced technologies—including AI-driven prediction models, machine learning, and potentially gene-editing frameworks—will be essential to ensure that future applications prioritize athlete safety, fairness, and long-term health.

## 10. Conclusions

Socceromics, the integration of genomics, proteomics, metabolomics, and microbiomics in soccer, stands at the forefront of precision sports science. These approaches enable personalized training, nutrition, and recovery protocols, offering tangible benefits in performance optimization, injury prevention, and rehabilitation. Genomic insights, such as SNPs linked to endurance, speed, or injury risk, can guide individualized programs, while metabolomics and proteomics provide real-time monitoring of energy metabolism and muscle repair. Microbiomics adds a complementary dimension, linking gut health to resilience, recovery, and immune function. Nonetheless, challenges remain, including the high cost of advanced technologies, the complexity of multi-omics data integration, and ethical concerns related to genetic/post-genetic privacy and player selection. Overcoming these barriers will be essential for translating Socceromics from research into daily practice. Ultimately, the integration of multi-omics offers a transformative framework for data-driven player management, laying the groundwork for a new era of precision performance and health in soccer players.

## Figures and Tables

**Table 1:** Overview of Single-Nucleotide Polymorphisms (SNPs) Related to Performance, Injury, Cognition, and Metabolism in Soccer Athletes.

Gene (Alias Symbols)	Polymorphism	rsID	Associated Trait(s)	Study Findings in Soccer Players
**CARDIOVASCULAR, VASCULAR & PERFORMANCE GENES**				
***ACE (ACE1, CD143)***	I/D	rs1799752/rs4646994	Endurance (I), strength/power (D), cardiac hypertrophy	D allele linked with strength and muscle hypertrophy; I allele enriched in endurance athletes. Several soccer studies show associations with left ventricular mass and injury susceptibility
***ACE (ACE1, CD143)***	intron variants	rs4341, rs4343	Cardiovascular efficiency	Used in multi-SNP panels in elite cohorts; may contribute to endurance profile
***AGT***	M235T	rs699	Blood pressure regulation, aerobic capacity	Mixed findings; some soccer studies show genotype-dependent hemodynamic responses
***NOS3 (ECNOS, eNOS)***	−786 T/C	rs2070744	Nitric oxide production, endurance	C allele reduces NO availability; may influence positional specialization (e.g., defenders vs. midfielders)
***NOS3 (ECNOS, eNOS)***	Glu298Asp	rs1799983	Vascular function	Linked to altered endothelial function; occasionally studied in soccer injury cohorts
***VEGF (VEGF-A, VPF)***	−634C/G or G > C	rs2010963	Angiogenesis, muscular recovery	Higher VEGF expression improves microvascularization and recovery; genotype patterns differ by player level
***ADRA2A (ADRAR) ***	various	—	Cardiovascular control, stress response	Helps modulate vascular tone; suggested to influence aerobic output in soccer cohorts
***ADRB1 ***	Arg389Gly	rs1801253	Cardiac output, VO_2_ response	Less commonly included in soccer-specific genetic panels; influences β_1_-adrenergic drive and exercise VO_2_ responses, with potential relevance for match-running capacity
***ADRB2 (ADRBR, BAR, B2AR, ARB2)***	Gln27Glu, Arg16Gly	rs1042714/rs1042713	Bronchodilation, cardiovascular response	Modulates oxygen delivery; variants used in youth academy performance analyses
***ADRB3 ***	Trp64Arg	rs4994	Lipolysis, thermogenesis	Appears in elite soccer genotype panels; influences body composition
**MUSCLE POWER, DAMAGE & HYPERTROPHY GENES**				
***ACTN3 ***	R577X	rs1815739	Sprint ability, power, injury risk	RR genotype overrepresented in elite soccer players; XX linked to more muscle injuries and reduced top speed
***TRIM63 (MuRF-1) ***	A/G	rs2275950	Muscle damage susceptibility	In Brazilian professional players: no significant association with muscle injury incidence
***TTN-AS1 ***	C/T	rs1001238	Inflammation, eccentric damage	CC genotype → higher CK, TNF- α, neutrophils in U20 players after eccentric training
***HIF1A (MOP1, HIF-1alpha, PASD8, HIF1*,** ***bHLHe78)***	Pro582Ser, other	Various	Hypoxia adaptation, power output	Some studies show power advantages in carriers
***CKM (CK-MM) ***	A/G	rs8111989	Muscle metabolism, injury risk	GG genotype linked to higher rates of muscle contracture in professional players/elite youth
***MLCK (smMLCK*,** ***MYLK1, MLCK1, KRP, MLCK108, MLCK210, MYLK-L)***	variants	rs2700352, rs28497577	Muscle stiffness, injury	Associated with contractile mechanics; linked to recurrent strains
***MSTN (GDF8, myostatin)***	K153R, E164K, P198A, I225T	Various	Muscle growth, hypertrophy	Performance-related effects; used in polygenic performance models
***MCT1 (MT, MCT, fabD, FASN2C, NET62, MCT1)***	A1470T	rs1049434	Lactate transport	AA genotype → more muscle injuries and slower lactate clearance in elite players
***AMPD1 (MAD, MADA)***	C34T	rs17602729	Energy turnover	CT genotype shows better creatine response and reduced lactate accumulation
**NEUROTRANSMISSION, COGNITION & PSYCHOLOGY GENES**				
***BDNF ***	Val66Met	rs6265	Neuroplasticity, motor learning	Val66Met interacts with cumulative soccer heading exposure and white-matter microstructure
***COMT ***	Val158Met	rs4680	Cognitive control, stress resilience	Affects decision-making and reaction under fatigue
***DBH (DBM) ***	promoter variants	rs1611115	Dopamine → norepinephrine conversion	Associated with motivation and arousal in sports contexts
***DRD1 (D1R) ***	variants	rs4532	Motivation, motor learning	Modulates reward sensitivity in training load
***DRD2 (D2R) ***	variants	rs1076560	Executive function	Affects reaction time and tactical decision-making
***DRD3 (D3R) ***	Ser9Gly	rs6280	Cognitive-emotional regulation	Relevant for performance stability
***SLC6A4 (5-HTT) ***	L/S	5-HTTLPR	Stress response, fatigue, aggression	L/S genotype most advantageous; SS associated with aggression, LL with low adaptability
***HTR2A (5-HT2A) ***	C/T	—	Cognitive processing	CC genotype > stability, reaction performance in academy players
***APOE (AD2) ***	ε2/ε3/ε4	rs429358, rs7412	Brain health	ε4 allele is a risk factor for worse memory performance associated with higher heading exposure
**CONNECTIVE TISSUE & INJURY GENES**				
***COL1A1 (OI4) ***	−1997G/T, +1245G/T	—	Ligament injury	Protective haplotypes reduce anterior cruciate ligament tear risk
***COL1A1 (OI4) ***	Sp1	rs1800012	Tendon/ligament stability	Included in several soccer injury cohorts
***COL1A2 ***	variants	—	Tendon/ligament remodeling	Seen in injury panels
***COL2A1 (STL1) ***	variants	—	Cartilage integrity	Included in anterior cruciate ligament studies
***COL5A1 ***	C/T	rs12722, rs13946	Tendon stiffness, anterior cruciate ligament injury	CC haplotype protective; widely used in soccer injury research
***COL12A1 (COL12A1L) ***	various	rs240736	Soft tissue injury	Associated with sports hernia in elite players
***COL22A1 ***	variants	rs11784270, rs6577958	Anterior cruciate ligament injury	Significant risk associations found in professional players
***ELN (WBS, WS, SVAS)***	variants	—	Medial collateral ligament and tendon stability	Certain variants protective
***EMILIN1 (DKFZp586M121, gp115, EMILIN)***	variants	—	Elastin fiber integrity	Appears in elite injury-mapping studies
***TNC (Tenascin-C) ***	variants	—	Tendon injury risk	Important extracellular matrix-related gene; linked to chronic tendinopathy
***MMP3 (STMY1, STMY)***	5A/6A	rs3025058	Anterior cruciate ligament injury	No association in Turkish professional cohort with repeated anterior cruciate ligament surgeries
***GEFT (ARHGEF25, p63RhoGEF)***	variants	—	Muscle repair	Seen in FC Barcelona injury panels
***LIF (CDF, DIA, HILDA)***	variants	—	Muscle recovery	Appears in elite injury multi-gene models
***HGF (SF, F-TCF, HGFB, HPTA)***	variants	—	Muscle regeneration	Included in referee injury-genetic panels
**ENERGY METABOLISM, MITOCHONDRIAL FUNCTION & BODY COMPOSITION**				
***PPARA (hPPAR, NR1C1)***	G/C	rs4253778	Fat metabolism, endurance	G allele enriched in endurance athletes; shown in soccer power vs. endurance studies
***PPARG (PPARG1, PPARG2, NR1C3, PPARgamma)***	Pro12Ala	rs1801282	Glucose sensitivity, power	Ala allele → improved insulin sensitivity, used in performance panels
***PPARD (NUC1, NUCII, FAAR, NR1C2, PPARB)***	T/C	rs2016520	Fat oxidation, endurance	Appears in elite endurance × soccer comparative genetics
***PPARGC1A (PGC-1α) ***	Gly482Ser	rs8192678	Aerobic capacity, mitochondrial biogenesis	Ser allele associated with lower VO_2_ max; used in elite-vs-subelite discrimination in soccer cohorts
***UCP1 (SLC25A7) ***	variants	—	Thermogenesis	Used in metabolism-related genetic work
***UCP2 (SLC25A8) ***	Ala55Val	rs660339	Efficiency of mitochondrial coupling	Included in performance prediction models
***UCP3 (SLC25A9) ***	−55C/T	—	Fat oxidation	Used in elite performance models
***FTO (KIAA1752, MGC5149, ALKBH9, IFEX9)***	common obesity SNPs	Various	Body composition	Sometimes used in academy-level profiling
***TFAM ***	variants	rs1937	Mitochondrial biogenesis	Important endurance marker; included in youth academy Genome-Wide Association Study (GWAS) follow-ups
**OXIDATIVE STRESS & DETOXIFICATION**				
***GSTM1 (MU, H-B)***	presence/absence	—	Detoxification	Affects response to oxidative load in match play
***GSTP1 (GSTP) ***	Ile105Val	—	Antioxidant defense	Modulates training-induced oxidative stress
***GSTT1 ***	null genotype	—	Oxidative metabolism	Associated with recovery capacity
***NRF2 (NFE2L2) ***	variants	—	Antioxidant control	Influences soccer positional demands
***CYP2D6 (CPD6, P450-DB1, CYP2D, P450C2D)***	various	—	Hormone/drug metabolism	Included in elite genetic panels
**HORMONE, GROWTH & DEVELOPMENT GENE**				
***LIN28A (LIN-28, FLJ12457, ZCCHC1, CSDD1)***	variants	rs6598964	Growth and biological maturation rate	Predicts biological maturation rate in youth players
***IGF2 (FLJ44734, IGF-II)***	variants	—	Muscle hypertrophy	Associated with professional athlete status
***SMAD6 (HsT17432) ***	variants	—	Tissue remodeling	Enriched in injury-prone players
**BONE ADAPTATION & REMODELING GENES**				
***VDR (NR1I1, PPP1R163)***	FokI	rs2228570	Bone strength, muscle power	Elite youth with CC (FokI) show higher bone mass and IGF-1
***VDR (NR1I1, PPP1R163)***	ApaI	rs7975232	Speed, explosive power	CC genotype enriched in elite youths
***VDR (NR1I1, PPP1R163)***	BsmI	rs1544410	Athlete status	Elite players less likely to carry A allele
***VDR (NR1I1, PPP1R163)***	TaqI	—	Bone integrity	Classic marker in soccer physiology
***P2RX7 (P2X7, MGC20089)***	Gln460Arg, others	rs1718119, rs3751143	Bone strength	Associated with cortical cross-sectional area and density in academy players
***RANK (TNFRSF11A, CD265, FEO, ODFR, TRANCE-R)***	variants	rs9594738	Bone remodeling	Baseline cortical differences observed in elite youths
***RANKL (TNFSF11, TRANCE, OPGL, ODF, CD254)***	variants	rs1021188	Bone turnover	Baseline differences but no training interaction
***OPG (TNFRSF11B, OCIF, TR1***)	variants	rs9594759	Bone density	Significant genotype–structure effects

*ACE = Angiotensin-Converting Enzyme; AGT = Angiotensinogen; NOS3 = Nitric Oxide (NO) Synthase 3; VEGF = Vascular Endothelial Growth Factor; ADRA2A = Alpha-2A Adrenergic Receptor; ADRB1 = Beta-1 Adrenergic Receptor; ADRB2 = Beta-2 Adrenergic Receptor; ADRB3 = Beta-3 Adrenergic Receptor; VO_2_ = Oxygen Uptake; ACTN3 = Alpha-Actinin-3; TRIM63 = Tripartite Motif-Containing Protein 63 (MuRF-1); TTN-AS1 = Titin Antisense RNA 1; HIF1A = Hypoxia-Inducible Factor 1-Alpha; CKM = Creatine Kinase, Muscle-type; MLCK = Myosin Light Chain Kinase; MSTN = Myostatin (GDF8); MCT1 = Monocarboxylate Transporter 1; AMPD1 = Adenosine Monophosphate Deaminase 1; BDNF = Brain-Derived Neurotrophic Factor; COMT = Catechol-O-Methyltransferase; DBH = Dopamine Beta-Hydroxylase; DRD1 = Dopamine Receptor D1; DRD2 = Dopamine Receptor D2; DRD3 = Dopamine Receptor D3; SLC6A4 (5-HTT) = Serotonin Transporter; HTR2A = Serotonin Receptor 2A; APOE = Apolipoprotein E; COL1A1 = Collagen Type I Alpha 1 Chain; COL1A2 = Collagen Type I Alpha 2 Chain; COL2A1 = Collagen Type II Alpha 1 Chain; COL5A1 = Collagen Type V Alpha 1 Chain; COL12A1 = Collagen Type XII Alpha 1 Chain; COL22A1 = Collagen Type XXII Alpha 1 Chain; ELN = Elastin; EMILIN1 = Elastin Microfibril Interfacer 1; TNC = Tenascin-C; MMP3 = Matrix Metalloproteinase-3; GEFT = Guanine Nucleotide Exchange Factor-T; LIF = Leukemia Inhibitory Factor; HGF = Hepatocyte Growth Factor; PPARA = Peroxisome Proliferator-Activated Receptor Alpha; PPARG = Peroxisome Proliferator-Activated Receptor Gamma; PPARD = Peroxisome Proliferator-Activated Receptor Delta; PPARGC1A = PPAR Gamma Coactivator 1-Alpha; UCP1 = Uncoupling Protein 1; UCP2 = Uncoupling Protein 2; UCP3 = Uncoupling Protein 3; FTO = Fat Mass and Obesity-Associated Gene; TFAM = Mitochondrial Transcription Factor A; GSTM1 = Glutathione S-Transferase Mu 1; GSTP1 = Glutathione S-Transferase Pi 1; GSTT1 = Glutathione S-Transferase Theta 1; NRF2 (NFE2L2) = Nuclear Factor Erythroid 2-Related Factor 2; CYP2D6 = Cytochrome P450 Family 2 Subfamily D Member 6; LIN28A = Lin-28 Homolog A; IGF2 = Insulin-Like Growth Factor 2; SMAD6 = Mothers Against Decapentaplegic Homolog 6; VDR = Vitamin D Receptor; P2RX7 = P2X Purinergic Receptor 7; RANK = Receptor Activator of NF-κB; RANKL = RANK Ligand; OPG = Osteoprotegerin*.

**Table 2:** Overview of the proteomics, metabolomics, microbiomics, and integrative omics studies related to soccer.

Omics Type	Study Focus	Key Findings	Research Examples
**Proteomics**	Muscle physiology, recovery	Post-match alterations in proteins linked to inflammation and oxidative stress, highlighting recovery demands	Martín-Sánchez et al. [25] found changes in α-1-antitrypsin and fibrinogen isoforms in professional players
**Metabolomics**	Energy metabolism, recovery	Metabolites such as 3-methylhistidine, taurine, and amino acids reflect muscle breakdown, energy turnover, and fatigue	Książek et al. [20] reported seasonal variation in vitamin D metabolites; Ra et al. [38] identified salivary fatigue markers in elite players
**Microbiomics**	Gut microbiota and performance	Greater abundance of butyrate- and succinate-producing bacteria in professionals, linked to recovery, immune function, and energy metabolism	Petri et al. [19] observed higher microbiota diversity in elite players; Urban et al. [14] linked training intensity to shifts in *Firmicutes*
**Integrative Omics**	Combined impact of multiple omics	Combining genomics, metabolomics, and proteomics data enhances the prediction of injury risk and recovery optimization	González et al. [123] showed integrated profiles predicted muscle and ligament injury risk; Orrù et al. [130] reported that lifelong football training improves muscle oxidative metabolism

## Data Availability

No new data were created or analyzed in this study. Data sharing is not applicable to this article.

## Supplementary Materials

The following supporting information can be downloaded at https://www.mdpi.com/article/10.3390/ijms27020749/s1.
