# Supplementary material for: Socceromics: A Systematic Review of Omics Technologies to Optimize Performance and Health in Soccer
Source: Int J Mol Sci. 2026 Jan 12;27(2):749. doi: 10.3390/ijms27020749 (PMC12841393; doi:10.3390/ijms27020749)
Supplement: Supplementary file 1 [file ijms-27-00749-s001.zip › Table S2.pdf]

Table S2. Database-specific search strategies and results for identifying omics-related studies in soccer

Database	Field scope/syntax	Search string	Search date	Results
**PubMed**	MeSH + Title/Abstract	(("Soccer"[Mesh] OR "association football"[tiab] OR football[tiab]) AND (Genomics[Mesh] OR "Polymorphism, Single Nucleotide"[Mesh] OR "single nucleotide polymorphism*"[tiab] OR SNP[tiab] OR SNPs[tiab] OR "genetic variant*"[tiab] OR "Copy Number Variations"[Mesh] OR "copy number variant*"[tiab] OR CNV[tiab] OR CNVs[tiab] OR "single nucleotide variant*"[tiab] OR SNV[tiab] OR SNVs[tiab] OR allele*[tiab] OR Metabolome[Mesh] OR metabolom*[tiab] OR Microbiota[Mesh] OR microbiom*[tiab] OR microbiot*[tiab] OR microbiomic*[tiab] OR Proteome[Mesh] OR proteom*[tiab] OR methylome[tiab] OR methylomic*[tiab] OR epigenom*[tiab] OR epigenetic*[tiab] OR sportomics[tiab] OR athlomics[tiab]))	August 25, 2025	277
**Web of Science (WoS)**	Topic (TS= title/abstract/keywords)	TS=(("soccer" OR "association football" OR "football") AND ("genomics" OR "polymorphism, single nucleotide" OR "polymorphisms" OR "SNP" OR "SNPs" OR "genetic variant*" OR "copy number variant*" OR "CNV" OR "CNVs" OR "single nucleotide variant*" OR "SNV" OR "SNVs" OR "allele*" OR "metabolome" OR "metabolomics" OR "microbiota" OR "microbiome" OR "microbiomics" OR "proteome" OR "proteomics" OR "methylome" OR "methylomics" OR "epigenome" OR "epigenetics" OR "epigenomics" OR "sportomics" OR "athlomics"))	August 25, 2025	329
**Scopus**	Title/Abstract/Keywords (TITLE-ABS-KEY)	TITLE-ABS-KEY(("soccer" OR "association football" OR "football") AND ("genomics" OR	August 25, 2025	362

		"polymorphism, single nucleotide" OR "polymorphisms" OR "SNP" OR "SNPs" OR "genetic variant*" OR "copy number variant*" OR "CNV" OR "CNVs" OR "single nucleotide variant*" OR "SNV" OR "SNVs" OR "allele*" OR "metabolome" OR "metabolomics" OR "microbiota" OR "microbiome" OR "microbiomics" OR "proteome" OR "proteomics" OR "methylome" OR "methylomics" OR "epigenome" OR "epigenetics" OR "epigenomics" OR "sportomics" OR "athlomics"))		
