# Supplementary material for: Socceromics: A Systematic Review of Omics Technologies to Optimize Performance and Health in Soccer
Source: Int J Mol Sci. 2026 Jan 12;27(2):749. doi: 10.3390/ijms27020749 (PMC12841393; doi:10.3390/ijms27020749)
Supplement: Supplementary file 1 [file ijms-27-00749-s001.zip › Table S4.pdf]

Reference	Design	Participants characteristics				Omics Technology	Tissue / Sample Type	Analytical Platform / Pipeline	Quality assessment
		Sample Size	Age	Sex	Level of play (elite, professional, academy)				
**De Almeida et al., 2023 [1]**	Observational cohort (one-season follow-up)	59 athletes (31 male, 28 female)	NR	Male + Female	Professional (Brazilian First Division)	Only genomics (ACTN3 R577X SNP).	Saliva	Genotyping assay (PCR-based analysis; ACTN3 R577X detection)	Moderate
**Tacal Aslan et al., 2024[2]**	Case–control genetic association study	20 football players + 64 sedentary controls	17–33 years	Male	Professional (Turkish club – Maltepespor)	Genomics (MCM6 rs4988235 polymorphism)	Peripheral blood	Real-Time PCR (TaqMan genotyping assay; Applied Biosystems StepOne Plus)	Moderate
**Jówko et al., 2023[3]**	Cross-sectional observational genetic association study	n = 181 (TR n = 94; CON n = 87)	20–23 years (peak bone mass; ≈21 y)	Male	Trained group: competitive athletes (soccer 4th league, handball 1st league, national-level wrestling, collegiate MMA); Control: physically active but non-training students	Genomics (VDR ApaI/BsmI/FokI; CALCR; COLIA1; SOD1; SOD2; GPx polymorphisms)	Peripheral venous blood	DNA isolation with QIAamp DNA Blood Mini Kit; SNP genotyping with TaqMan assays (Applied Biosystems) on real-time PCR (Rotor-Gene, Qiagen)	Moderate
**Kayumov et al., 2022 [4]**	Cross-sectional genetic association study	n = 91	NR	Male	Youth football academy teams (Tashkent)	Genomics (ACE I/D, PPARGC1A, AT2R1 polymorphisms)	Buccal epithelial cells	PCR-based genotyping (polymerase chain reaction)	Low
**Nunes et al., 2021[5]**	Analytical profiling / exploratory metabolomic screening	n = 10	22–26 years	Male	University-level football players (recreational/competitive amateur)	Metabolomics	Sweat (eccrine sweat, forehead/face)	LC–MS/MS (UHPLC + triple quadrupole MS; ESI; MRM/SRM workflow)	Moderate
**Kawata et al., 2020 [6]**	Cross-sectional pilot association study	n = 17 (complete datasets)	Mean ≈ 15–17 years	Male	High-school / youth competitive (scholastic) football players	Proteomics (blood biomarkers)	Serum (venous blood)	Simoa™ ultrasensitive single-molecule ELISA platform (Quanterix) for tau, NfL, GFAP	Moderate
**Subak & Muniroglu, 2020[7]**	Case–control genetic	n = 60 (30 referees, 30 controls)	Referees: 23.33 ± 2.52 yr;	Male	Competitive soccer referees (performance-	Genomics (SNP genotyping)	Buccal epithelial cells	Buccal swab DNA extraction (MagPurix), PCR	Moderate

	association study		Controls: 22.47 ± 1.92 yr		tested) vs. sedentary controls			amplification, agarose gel electrophoresis for ACE I/D, and Next-Generation Sequencing (Illumina MiSeq; Miseq Reporter + IGV) for SOX15 and HGF	
**Hassan & Shady, 2020[8]**	Descriptive cross-sectional genetic association study	n = 68	18.86 ± 2.35 yr	Male	First division of the Saudi Youth League (competitive/elite youth)	Genomics (candidate gene SNP genotyping of ACTN3 R577X)	DNA sample for PCR-based genotyping (biological source not specified)	PCR-based ACTN3 polymorphism detection in a private laboratory, with subsequent statistical analysis (ANOVA, LSD post hoc tests, Pearson correlations) using SPSS v25	Moderate
**Ük et al., 2020 [9]**	Case–control genetic association study	22 players; 68 controls	18–23 yr	Male	Professional soccer players	Genomics (candidate gene SNP genotyping of PPARA rs4253778)	Buccal cells (DNA extraction)	Real-time PCR using TaqMan SNP Genotyping Assay (Applied Biosystems)	Moderate
**Ioffe et al., 2020 [10]**	Case–control genetic association study	95 football players (41 with sports hernia; 54 controls)	17–33 yr	Male	High-profile football players	Genomics (candidate gene SNP genotyping of COL1A1 rs1800012, MCT1 rs1049434, COL12A1 rs240736)	Buccal epithelium (DNA extraction)	-Real-time PCR using TaqMan Pre-designed SNP Genotyping Assays; Random Forest & MDR for association modeling	Low
**Cocci et al., 2018 [11]**	Comparative genetic association study across sports	113 athletes (55 soccer; 37 combat; 21 motorcycle)	22–26 yr	Male	Elite / professional national-level athletes	Genomics (ACE I/D, ACTN3 R577X, PPARA rs4253778, CK-MM rs8111989)	Buccal cells (DNA extraction)	PCR amplification + RFLP; agarose gel electrophoresis	Moderate

**Honarpour et al., 2017[12]**	Case–control genetic association study	90 elite players; 200 controls	NR	Male	Elite / Top-level professional Iranian soccer player	Genomics (candidate gene SNP genotyping of ACTN3 R577X)	Whole blood (EDTA tubes)	PCR + RFLP using DdeI restriction enzyme; sequencing confirmation	Moderate
**Kenger et al., 2023[13]**	Cross-sectional observational study	20 players		Male	Professional football players (Turkish Second League)	Metagenomics (16S rRNA gene sequencing of gut microbiota)	Fecal samples	16S rRNA sequencing; bioelectrical impedance analysis; 3-day dietary intake assessment	Moderate
**Pintus et al., 2020[14]**	Longitudinal observational metabolomics study	21 players		Male	Professional soccer players (Serie A, Italy)	Metabolomics (1H-NMR)	Urine	1H-NMR spectroscopy of low-molecular-weight metabolites (<1.5 kDa)	Moderate
**Cięszczyk et al., 2016[15]**	Case–control genetic association study	106 players; 115 controls	NR	Male	Professional football players (top Polish clubs)	Genomics (candidate SNP genotyping of ACE I/D)	Buccal cells	PCR amplification of ACE I/D region, agarose gel electrophoresis	Moderate
**Wessner et al., 2019 [16]**	Case–control genetic association study	143 team sport athletes (82 soccer, 61 handball) + 56 power athletes + 86 endurance athletes + 216 controls	18–83 yrs (team sport athletes ~12 yrs younger than others)	Mixed (male & female)	Elite national/international level	Genomics (candidate gene SNP genotyping for ACTN3 R577X, ADRB1, ADRB2, ADRB3 variants)	Saliva (genomic DNA extraction)	Real-time PCR using TaqMan SNP Genotyping Assays (Applied Biosystems)	Moderate
**Massidda et al., 2015 [17]**	Case–control genetic association study across 5 season	173 elite male football players	19.4 ± 5.2 yrs	Male	Elite (Serie A, Primavera, Allievi, Giovanissimi)	Genomics (MCT1 rs1049434 SNP genotyping)	Buccal swab DNA	PCR amplification + restriction enzyme digestion (BccI); agarose gel electrophoresis	Moderate
**Marinich et al., 2013[18]**	Cross-sectional genetic association study	N= 125	Youth athletes	Male	Youth academy (sports school football players)	Genomics (5HTT L/S and 5HT2A C/T polymorphisms)	Buccal epithelial smear	PCR + RFLP (MspI digestion for 5HTT)	Low

**Massidda et al., 2012[19]**	Case–control genetic association study	59 athletes + 31 controls	NR	Male	Elite (National & International level)	Genomics (ACE I/D & ACTN3 R577X polymorphisms)	Buccal swab (DNA extraction)	PCR amplification; ACE genotyping via agarose gel electrophoresis; ACTN3 genotyping via PCR + DdeI digestion + polyacrylamide gel electrophoresis	Moderate
**Pimenta et al., 2012 [20]**	Experimental study assessing genotype-based responses to eccentric training RCT	37 professional soccer players	NR	Male	Professional (Brazilian First Division)	Genomics (ACTN3 R577X genotyping)	Peripheral blood (DNA extraction)	PCR amplification + DdeI restriction digest + polyacrylamide gel electrophoresis	Moderate
**Kambouris et al., 2014 [21]**	Pilot genomic profiling study	7 players	NR	Male	Elite professional (Fulham FC, English Premier League)	Genomics (multi-SNP panel)	Buccal swabs	Sequenom MassARRAY SNP genotyping platform (PCR → iPLEX primer extension → MALDI-TOF MS)	Low
**Proia et al., 2014 [22]**	Case–control genetic association study	60 players; 30 controls	22.5 ± 2.2 yr	Male	Professional (Italian Serie B–C)	Genomics (PPARα intron 7 G/C genotyping)	Whole blood (genomic DNA)	PCR–RFLP using TaqI enzyme	Moderate
**Orrù et al., 2022[23]**	Cross-sectional molecular comparison (football players vs. controls)	9 lifelong football players; 9 controls	64–71 yr	Male	Veteran lifelong football players (recreational, ≥10 years)	Proteomics + Metabolomics	Vastus lateralis muscle biopsies	LC-MS/MS (Orbitrap), SDS-PAGE in-gel digestion; MS-based targeted metabolomics (AA & acylcarnitines), polyamine fluorometric assays	High
**Ulucan et al., 2015[24]**	Genetic association study	25 players	NR	Male	Professional Turkish soccer players	Genomics (ACE I/D & ACTN3 R577X SNP genotyping)	Peripheral blood (DNA extraction)	PCR and PCR–RFLP; agarose gel electrophoresis (ACE) and polyacrylamide gel electrophoresis (ACTN3)	Moderate

**Yang et al., 2025 [25]**	Genetic association study (elite vs sub-elite vs controls)	142 soccer players; 107 controls	Players: 13–15 y; controls: 13–14 y	Male	Elite and sub-elite youth soccer players (Chinese academies/pro club)	Genomics (VDR SNP genotyping: ApaI rs7975232, BsmI rs1544410, FokI rs2228570)	Buccal mucosa (oral swabs)	PCR-based SNP genotyping using specific primers; products checked by agarose gel electrophoresis	Moderate
**Contrò et al., 2018 [26]**	Case–control genetic association study using multivariate logistic regression	60 professional soccer players; 60 sedentary controls	Players: 22.5 ± 2.2 y; controls: 21.2 ± 2.3 y	Male	Professional Italian soccer players	Genomics (PEPs: PPARα, PPARGC1A, NRF2, ACE, CKMM — PCR-RFLP genotyping)	Venous blood (whole blood DNA)	PCR-RFLP with specific restriction enzymes for each polymorphism	Low
**Artells et al., 2016 [27]**	Observational longitudinal cohort (7 seasons) with genetic association	N= 60	25.5 ± 2.5 y (range 19–35)	Male	Top elite (UEFA Champions League, French L1, La Liga, Premier League)	Genomics (SNP genotyping – ELN rs2289360)	Venous blood (DNA extraction)	TaqMan allelic discrimination assay (Real-Time PCR, Illumina ECO system)	Moderate
**Sha et al., 2019 [28]**	Randomized controlled trial (2-month intervention)	38 (28 hydrogen-rich water / 10 control)	~12–14 y (juvenile)	Female	Academy / youth elite (Suzhou Sports School)	Metabolomics (ELISA oxidative/inflammatory biomarkers) + Microbiomics (16S rDNA sequencing)	Blood (serum, whole blood) & stool samples	ELISA for MDA, SOD, T-AOC, IL-1, IL-6, TNF-α; Blood cell analyzer; Automatic biochemical analyzer (HGB, BUN, CK); 16S rDNA sequencing (Novagene pipeline)	Moderate
**Lifanov et al., 2014 [29]**	Randomized placebo-controlled supplementation trial	21 football players (11 creatine / 10 placebo)	NR	Male	Competitive football players	Genomics	Blood (DNA)	Genotyping of AMPD1 C34T and PPARG Pro12Ala (method not clearly specified in abstract)	Low
**González et al., 2024 [30]**	Prospective longitudinal cohort (2 seasons)	24 players	Adult	Female	Elite professional (FC Barcelona, First Team)	Genomics + Metabolomics	Blood (DNA); Urine (metabolites)	Genotyping of 108 SNPs (92 after QC); UPLC–MS/MS for 61 metabolites; GPS	High

								workload using WIMU PRO; Frailty Cox models & DLNM	
**Massidda et al., 2024 [31]**	Longitudinal cohort (10-year injury follow-up)	N= 64	Adult (23.1 ± 5.5 y)	Male	Top-level professional (Serie A, Italy)	Genomics (4 SNP panel)	Buccal swabs (DNA)	PCR genotyping; Total Genotype Score (TGS) computed from ACE I/D, ACTN3, COL5A1, MCT1 polymorphisms	High
**McAuley et al., 2024 [32]**	Cross-sectional genetic association study	N=149	U12–U23 (youth to young adult)	Male	Academy (4 English professional club academies)	Genomics (22 SNPs)	Buccal swabs (DNA)	PCR-based SNP genotyping; single SNP regression; unweighted & weighted Total Genotype Scores (TGS, TWGS); Benjamini–Hochberg FDR correction	Moderate
**Maestro et al., 2022 [33]**	Cross-sectional cohort genetic association study	N= 122	Mean 23.4 ± 5.1 y	Male	Professional (LaLiga Santander & Smartbank, Spain)	Genomics (6 SNPs: AMPD1, ACE, ACTN3, CKM, MLCK ×2)	Buccal swab (DNA)	Single Nucleotide Primer Extension (SNPE) using SNaPshot Multiplex Kit; capillary electrophoresis (ABI3500); GeneMapper 5.0; Total Genotype Score (TGS) computation	High
**Del Coso et al., 2022 [34]**	Cross-sectional genotype–performance–injury association study	N= 191	~23 years	Female	Professional (Women's Spanish First Division – Primera Iberdrola)	Genomics (ACTN3 R577X, rs1815739)	Buccal swabs (DNA)	SNPE (SNaPshot Multiplex Kit) + capillary electrophoresis (ABI3500); TaqMan SNP Genotyping Assay (Applied Biosystems); Real-	High

								Time PCR (7500 Fast System)	
**Diogenes et al., 2010 [35]**	Longitudinal observational (≈6-month follow-up)	N= 46	11.8–14.2 years	Male	Competitive youth academy (U13 & U15 Botafogo Soccer Club, Brazil)	Genomics (candidate gene polymorphisms: VDR FokI and TaqI)	Whole blood (venous)	DNA extraction (GFX Genomic Blood Purification kit), PCR amplification of VDR loci, restriction enzyme digestion (FokI, TaqI) and fragment analysis (PCR-RFLP); bone phenotyping by DXA (Lunar Prodigy Advance) plus hormonal assays (IGF-1, testosterone, PTH, bone ALP)	Moderate
**Falahati & Arazi, 2023[36]**	Quasi-experimental, crossover (pre/post MICE & HIIE)	56 total (29 trained, 27 untrained)	Adult men	Male	Trained group: national league soccer players; Untrained: recreationally active men	Genomics (candidate gene): ACE I/D polymorphism	Whole blood	DNA extraction (salting-out), PCR detection of ACE I/D (agarose gel electrophoresis); cardiac biomarkers quantified via ELISA (NT-proBNP) and hs-cTnI (AccuTnI assay) before/after HIIE & MICE	Moderate
**Meckel et al., 2019 [37]**	Cross-sectional genetic association study	60 soccer players (total athletes = 170; + 51 controls)	Young athletes	Male	National-level youth soccer players	Genomics (candidate genes: PPARD + ACTN3)	Buccal epithelial cells	DNA extraction, TaqMan allelic discrimination assay for SNP genotyping	Moderate
**Monnerat et al., 2018[38]**	Cross-sectional genetic population study	25 elite soccer players	25.5 ± 4.3 yrs	Male	Professional (Brazilian first division)	Genomics (10 SNPs linked to performance)	Buccal epithelial cells	DNA extraction (INVISORB Spin Forensic Kit), TaqMan allelic	Moderate

								discrimination assay (qPCR); comparative PCA + Fst using 1000Genomes data	
**Varley et al., 2018 [39]**	Prospective cohort (pre–post 12-week training)	N=99	≥16 years (adolescent academy players)	Male	Elite academy footballers (UK full-time academies)	Genomics (10 SNPs in P2X7R, RANK/RANKL/OPG, Wnt, NF-κB, IL6)	Saliva	Fluorescence-based competitive allele-specific PCR assays; Hardy–Weinberg checks; repeated-measures ANOVA for genotype × time	Moderate
**Terrell et al., 2008 [40]**	Multicenter cross-sectional genetic association study	N= 195	18–30 yr	Male & Female	US collegiate athletes (NCAA-level programs)	Genomics (APOE, APOE promoter G-219T, Tau exon 6 SNPs)	Blood (WBC) & buccal mouthwash samples	PCR + RFLP genotyping (AflIII / HhaI digestion; PCR-RFLP per Lambert et al., Poorkaj et al.)	High
**Gouveia et al., 2024 [41]**	Longitudinal metabolomics study (pre–post match across 3 games)	N= 14	Youth	Female	Elite professional soccer players	Metabolomics	Urine	NMR spectroscopy; OPLS-DA; VIP scoring	High
**Flore et al., 2024[42]**	Cross-sectional genetic association study	N=55	15–18 y (mean 16.65 ± 1.55)	Male	Elite youth (Italian professional club: Cagliari Calcio, Primavera/Allievi/Giovanissimi)	Genotyping of VDR SNPs (rs2228570, rs7975232, rs1544410)	Buccal swab	PCR–RFLP + agarose/polyacrylamide gel electrophoresis	Moderate
**Fagundes et al., 2024[43]**	Retrospective observational genetic association study	N= 46	Mean 21.3 ± 1.14 y	Male	Brazilian First Division professional club	Genotyping of MuRF-1/TRIM63 SNP (rs2275950)	Blood	qPCR (TaqMan Genotyping Assay)	Low
**González-García & Varillas-Delgado, 2024 [44]**	Longitudinal prospective observational study	N= 37	18-40	Male & Female	Amateur (Spanish 4th division, U18, and women's 3rd division)	Genomics (ACE, ACTN3, AMPD1, CKM, MLCK polymorphisms)	Buccal swab	SNPE (SNaPshot Multiplex) + capillary electrophoresis	Moderate
**Varillas-Delgado, 2024 [45]**	Longitudinal cohort study	N= 161	Adults	Male	Professional (LaLiga + LaLiga Smartbank)	Genomics (ACE, ACTN3, AMPD1, CKM, MLCK polymorphisms)	Buccal swabs	SNPE (SNaPshot	Moderate

								Multiplex) + Capillary electrophoresis (ABI3500)	
**Del Coso et al., 2024[46]**	Prospective observational cohort (season-long)	N=315	Adults	Male	Top-tier professional (Spanish LaLiga, 1st division)	Genomics (single SNP, ACTN3 rs1815739 / R577X)	Buccal swabs (buccal smear)	TaqMan SNP Genotyping Assay (Applied Biosystems) on ABI 7500 Fast Real-Time PCR; duplicate genotyping in a subsample; HWE checked; comparison with 1000 Genomes	High
**Pimenta et al., 2024 [47]**	Prospective intervention with standardized eccentric-focused training; repeated measures (pre, 24h, 48h)	N= 46	18–20 y (U20)	Male	Elite youth (U20 players from Brazilian first division clubs)	Genomics (single SNP, TTN-AS1 rs1001238) + classical biomarkers	Whole blood (EDTA) / serum	DNA by salting-out; TaqMan allelic discrimination for rs1001238 on StepOnePlus Real-Time PCR; inflammatory and damage markers via automated analyzers (CK, hs-CRP, TNF-α, IGF-1)	Moderate
**de Almeida et al., 2024 [48]**	Retrospective observational cross-sectional study	Main cohort: 23 players (115 GPS+CK datasets); Replication cohort: 18 players (90 datasets)	Main: 25 ± 3.9 y; Replication: 29 ± 4.8 y	Male	Professional (Brazilian Série A, first division)	Genomics (ACTN3 rs1815739 genotyping)	Blood (main cohort); Saliva (replication cohort)	Main: DNA extraction via salting-out; ACTN3 genotyping via PCR + DdeI digestion + agarose electrophoresis. Replication: TaqMan SNP assay using Real-Time PCR. CK	Moderate

								assay via Reflotron Analyzer; GPS external load tracking (Catapult)	
**Albuquerque et al., 2024 [49]**	Cross-sectional genetic association study	627 football players across 4 categories (U15=172; U17=166; U20=161; Professionals=128)	U15–U20 youth + adults	Male	Elite: First-division Brazilian clubs (youth & professional squads)	Genomics (candidate-gene SNP analysis: ACTN3 rs1815739, ACE I/D)	Buccal epithelial cells (cheek swabs)	DNA extracted with Invisorb Spin Forensic Kit; ACTN3 genotyped via TaqMan assay on Bio-Rad CFX Opus 96; ACE I/D genotyped via SYBR Green PCR using 3-primer strategy to avoid preferential amplification; fragment visualization on 3% agarose gel; 10% duplicate genotyping with 100% concordance; HWE tested; SNPassoc used for genetic models	Moderate
**Varillas-Delgado et al., 2024 [50]**	Prospective observational cross-sectional genetic association study	N=168	>18 y	Female	Spanish second-division amateur women's clubs	Genomics (candidate-gene SNP panel: ACTN3 rs1815739, ACE rs4646994, AMPD1 rs17602729, CKM rs8111989, MLCK rs2700352 & rs28497577)	Buccal epithelial cells (cheek swabs)	DNA extracted via QIACube automated system; SNP genotyping via Single Nucleotide Primer Extension (SNaPshot Multiplex Kit); capillary electrophoresis on ABI 3500; allele calling with GeneMapper 5.0; HWE tested; χ^2 + standardized residuals used for	Moderate

								genotype–injury associations	
**Bülbül et al., 2024 [51]**	Case–control genetic association study	105 players total (≈41–44 with ≥2 ACL surgeries vs 61 without ACL surgery)	18–35 y	Male	Turkish professional football leagues	Genomics (single candidate SNP: MMP3 rs3025058, 5A/6A promoter polymorphism)	candidate SNP: MMP3 rs3025058, 5A/6A promoter polymorphism) EDTA-treated whole venous blood	Genomic DNA isolated with a commercial kit (GeneAll Exgene Clinic SV). MMP3 rs3025058 genotyped by PCR–RFLP: amplification of 129 bp fragment (Primer3-designed primers), digestion with ThtIII1 restriction enzyme, fragments (129 bp for 6A; 97+32 bp for 5A) separated on 3% NuSieve agarose gel, visualized under UV.	Moderate
**Varillas-Delgado, 2025 [52]**	Longitudinal observational pilot study with embedded pharmacogenetic / nutrigenetic analysis	N= 48	>18 y (adult)	Male	Spanish professional league, Hypermotion division (2nd tier)	Targeted genomics panel (candidate SNPs) combined with biochemical markers: ACE (rs4646994), ACTN3 (rs1815739), AMPD1 (rs17602729), CKM (rs8111989), HFE (rs1799945), MLCK (rs2700352, rs28497577); integrated into a Total Genotype Score (TGS)	Buccal epithelial cells for DNA; venous blood for biochemistry (serum ferritin, Hb, Hct, serum iron)	DNA: automated extraction (QIACube), SNPE genotyping (SNaPshot Multiplex, Thermo Fisher), fragment analysis on ABI3500 with GeneMapper 5.0. SNPs checked for HWE. Biochemistry: 6 standardized blood draws per season (fasted, EDTA + serum tubes) with routine clinical assays for ferritin, Hb, Hct, serum iron. Performance:	Moderate

								WIMU PRO™ GPS (RealtrackSystems) with SPRO™ software	
**Gouveia et al. 2025 [53]**	Cross-sectional observational study with repeated pre-/post-match urine sampling across 6 games	14 players; 102 urine samples (51 pre-, 51 post-match)	Adult	Female	Professional women's team competing in national and regional championships	Untargeted metabolomics (^{1}H-NMR) of urine linked with dietary intake of antioxidant micronutrients (vitamins A, C, E, Se, Zn, Mg)	Urine	NMR: Bruker 600 MHz spectrometer (^{1}H-NMR); sample prep with phosphate buffer (TSP in D_2O). Spectral processing in TopSpin 3.5; data matrix built in R with PepsNMR; metabolite identification using Chenomx and HMDB. 41 metabolites retained. Diet: 11 records analysed in Dietbox® with multiple Brazilian composition tables; adequacy vs DRIs and sport-specific recommendations.	Moderate
**Malefo et al., 2025 [54]**	Within-subject pre–post exercise observational study	N=12	Young adults (18–30 y)	Male	University team (Sefako Makgatho Health Sciences University)	Untargeted metabolomics (GC×GC–TOFMS) of sweat to identify signature metabolites and pathways pre- vs post-exercise	Sweat from forearm (Macro-duct® collectors)	Sample prep: protein-precipitation/extraction with 70:30 methanol–acetonitrile + internal standard (3-phenylbutyric acid), derivatization with methoxyamine in pyridine then BSTFA + 1% TMCS. Platform: LECO Pegasus 4D GC×GC–TOFMS	Moderate

								with cryomodulation. Peak detection & deconvolution in ChromaTOF (S/N 200, ≥3 apex points); identification via NIST libraries (similarity ≥70%) plus level-3 tentative IDs per Schymanski. QC: extraction blanks, system blanks, FAMES for RI and system suitability. 57 signature metabolites identified (carboxylic acids, ketones, alcohols, aldehydes, aromatics, hexoses, hydroxy fatty acids, etc.).	
**Rodas et al., 2025 [55]**	Longitudinal observational metabolomics study (10-month season)	N=41	U18	Male	FC Barcelona academy (elite youth)	Untargeted metabolomics (LC-MS / UPLC-QTOF-MS)	First-morning urine	UPLC-QTOF-MS (Agilent 1290–6550) + BEH C18 column; positive ESI, m/z 70–1000. QC pipeline: pooled QC (10 injections), QC every 6 samples, blanks, DDA-MS/MS library acquisition. Preprocessing: MassHunter → XCMS peak picking → QC-SVR batch correction → PQN	High

								normalization → stringent filtering (QC RSD < 20%, QC/blank >9). Annotation via HMDB, MassBank, MetaboBase, ReSpect, RIKEN (error <20 ppm, RT ±0.2 min).	
**Manchón-Davó et al., 2025 [56]**	Retrospective case–control genetic association study	N= 268	Adult professionals (LaLiga; 18–35+ years)	Male	Top-tier professional (LaLiga, 10 teams)	Candidate gene genotyping (COL5A1 rs12722 C/T)	Buccal epithelial cells (buccal swab)	DNA extraction with organic method + Amicon® Ultra columns; genotyping by TaqMan SNP Genotyping Assay (rs12722, C____370252_20) on Applied Biosystems 7500 Fast Real-Time PCR. QC: internal blank/negative controls, contamination monitoring, re-genotyping of ambiguous calls, duplicate genotyping in 69 random samples (100% concordance).	High
**Bulgay et al., 2023 [57]**	Prospective longitudinal intervention with genetic stratification (pre–post 6-week training)	N= 22	24.79 ± 4.56 years	Male	Professional – North Macedonia Super League	Candidate gene genotyping (ACTN3 rs1815739, PPARA-α rs4253778)	Buccal / oral epithelial cells (cotton swab)	DNA isolated with Canvax kit; genotyping by TaqMan SNP Genotyping Assays for ACTN3 rs1815739 and PPARA-α	Moderate

								rs4253778 on StepOne Plus real-time PCR. Reaction mix detailed (Master Mix + assay + DNA). Alleles identified via VIC/FAM probes; multicomponent plots shown for CC/CT/TT (ACTN3) and CC/CG/GG (PPARA).	
**Kanope et al., 2023 [58]**	Observational cross-sectional, population-genetic replication study	44 U20 players	U20	Male	Elite / first-division under-20 (Brazil)	Whole-genome sequencing with targeted analysis of performance-related SNP panel	Buccal epithelial cells (buccal swabs)	DNA extracted with MagMAX™ DNA Multi-Sample kit; libraries prepared with TruSeq DNA PCR-Free (Illumina); sequenced on NovaSeq6000 (mean depth ~44×). Reads mapped to GRCh38 and variants called with DRAGEN Germline App v3.7.5. Performance-related SNP genotypes retrieved from VCF and manually checked in BAM using IGV. HWE tested in R (“genetics” package). Population	Moderate

								genotypes from 1000 Genomes via Ensembl. Genetic distances and molecular variance (FST) computed with Arlequin v3.5; PCA with Past3; phylogenetic tree with iTOL v6. Ethics approval and informed consent reported.	
**Yang et al., 2023 [59]**	Observational cross-sectional genetic association study (elite vs sub-elite vs non-athletic controls)	N= 249	13–15 years (63 × 13 y; 47 × 14 y; 32 × 15 y)	Male	Elite & sub-elite youth academy / school football (Chinese Han)	Targeted candidate-gene genotyping (ACE I/D; ACTN3 R577X)	Oral mucosa / buccal epithelial cells (oral flocking swabs)	DNA extracted with the TSINGKE silica gel adsorption kit. PCR amplification of ACE and ACTN3 with specific primers; products checked by agarose gel electrophoresis. PCR products gel-purified and Sanger sequenced (BDT reaction; capillary electrophoresis). Genotypes called with GeneMapper 4.1.	Moderate
**McAuley et al., 2023[60]**	Observational cross-sectional genetic association study	N= 53	U13–U18; mean 16.28 ± 1.27 years	Male	Category 3 English academies (youth, outfield players only)	Targeted SNP genotyping on custom array (8 performance/skill-related SNPs: ADRB2 rs1042714, ACE rs4341, BDNF rs6265, COMT rs4680, DBH rs1611115, DRD1 rs4532, DRD2	Saliva / buccal epithelial cells (self-administered buccal swabs)	DNA extracted with Qiagen chemistry on automated Kingfisher FLEX system. Genotyping on a custom Affymetrix / Thermo Fisher Axiom array;	Moderate

						rs1076560, DRD3 rs6280)		hybridisation on GeneTitan, processed with Axiom Analysis Suite. QC: SNP call rate >95%, sample call rate >95%, Fisher's linear discriminant >3.6, MAF >0.05. HWE tested; LD checked via LDlink (1000 Genomes EUR).	
**Ružić et al., 2023 [61]**	Comparative genetic association study	56 total (31 national-team players; 25 elite non-national players)	18–35 yrs	Male	Croatian National Team; First Croatian League elite clubs (Dinamo Zagreb, Hajduk Split)	Targeted SNP genotyping (ACTN3 rs1815739; ACE rs1799752; ADRB3 rs4994	Buccal mucosa cells (buccal swabs)	DNA extraction: Chelex 100. PCR amplification via PyroMark PCR Kit (Qiagen). Genotyping: Pyrosequencing (PyroMark Q24) for ACTN3 & ADRB3; Capillary electrophoresis (Genetic Analyzer 3131, Applied Biosystems) for ACE; fragment analysis via GeneMapper ID-X.	Moderate
**Kurtulus et al., 2023 [62]**	Comparative genetic association study (endurance vs. power/endurance vs. controls)	214 total: 53 wrestlers, 71 football players, 34 long-distance runners, 56 controls	22–26 yrs	Male	National- and international-level Turkish elite wrestlers, football players, and long-distance runners	Targeted SNP genotyping (PPARα intron 7 G/C, rs4253778)	Whole blood	DNA extraction via phenol/chloroform; genotyping via PCR–RFLP using TaqI enzyme; agarose gel electrophoresis for fragment visualization	Moderate

**Yang et al., 2023 [63]**	Observational cross-sectional genetic association study (elite vs sub-elite, by playing position)	N= 142	13-15yrs	Male	Elite (National Youth Football Super League) and sub-elite (regional leagues) Chinese youth football players	Targeted SNP genotyping (ACTN3 R577X, rs1815739)	Oral mucosa / buccal epithelial cells	DNA extraction from oral mucosa using TSINGKE silica gel kit; PCR amplification of ACTN3 exon 16; agarose gel electrophoresis; gel extraction and Sanger sequencing; genotype calling with Sequencing Analysis 5.2	
**Petr et al., 2022 [64]**	Cross-sectional genetic association study	99 elite male soccer players + 107 controls	Adults (mean 25.4 ± 4.5 y)	Male	First & Second Division professional Czech league players	Targeted SNP genotyping of 7 variants (ACTN3, ACE, NOS3, AMPD1, UCP2, BDKRB2, IL1RN)	Buccal epithelial cells	DNA extraction via QIAamp DNA Mini Kit; PCR amplification (Labcycler thermocycler); restriction enzyme digestion; agarose gel electrophoresis; genotype visualization (G:BOX Chemi HR16); statistical modelling including TGS	Moderate
**Soriano et al., 2022 [65]**	Prospective longitudinal cohort (within-season repeated measures) with small concussed subcohort	33 Division I male collegiate American football players (4 with diagnosed concussion, extra sampling)	18–23 years (mean 19.3 ± 1.4)	Male	NCAA Division I collegiate American football	Microbiomics (16S rRNA gene amplicon sequencing) + serum protein biomarkers (GFAP, NF-L, S100β, SAA, Tau, UCH-L1)	Stool, saliva, and blood serum	DNA extraction from stool (QIAamp PowerFecal Pro) and saliva (Norgen kit); full-length 16S rRNA amplicon sequencing on Oxford Nanopore MinION (16S Barcoding Kit SQK-16S024); basecalling with Guppy, adapter trimming with	High

								Porechop; taxonomic classification with Kraken v1.1.1 and visualization via Pavian; diversity analyses with scikit-bio (Shannon/Simpson, Weighted UniFrac, ANOSIM); functional prediction with PICRUSt2; serum biomarkers quantified via SIMOA Neurology 4-plex (HD-X, Quanterix) and ELISAs for S100β and SAA	
**Varillas-Delgado et al., 2022[66]**	Cross-sectional / case–control "transversal prospective" genetic association study	N = 452 total: 160 elite endurance athletes, 132 professional football players, 160 non-athlete controls	Adults	Male	Elite endurance: world-class/professional cyclists (UCI World Tour) and elite long-distance runners (Olympic/World/European level); Professional football: Spanish La Liga & Liga Smartbank players (some UEFA CL/EL, internationals); Controls: non-athletic men, age-matched	Targeted genomics (candidate polymorphism panel, polygenic profiles)	Oral mucosa (buccal swabs) → genomic DNA	DNA extracted on QIACube; multiplex PCR–Single Nucleotide Primer Extension (PCR-SNPE) genotyping (SNaPshot kit, Applied Biosystems) for panel of SNPs in CYP2D6, GSTM1, GSTP, GSTT (liver metabolism); HFE, AMPD1, PGC1A (iron/energy); ACE, NOS3, ADRA2A, ADRB2, BDKRB2 (cardiorespiratory); ACE, ACTN3,	Moderate

								AMPD1, CKM, MLCK (muscle injuries).	
**de Almeida et al., 2022 [67]**	Prospective observational cohort / pilot genetic association study	n = 83 professional male soccer players; 99 non-contact muscle injuries recorded over 2018–2020 seasons	Adults	Male	Professional, 1st and 2nd divisions of the Brazilian Championship (Brasileirão)	Targeted genomics (candidate polymorphisms ACTN3 & ACE)	Peripheral blood (leukocytes)	DNA extracted from 4 mL blood via salting-out; genotyping of ACTN3 R577X (rs1815739) by PCR–RFLP (DdeI digestion, 3% agarose); ACE I/D (rs4646994) by PCR and 1% agarose, with second insertion-specific PCR to avoid D-allele preferential amplification.	Moderate
**Kim et al., 2022[68]**	Longitudinal observational study	N= 14	10–13 years	Male	Youth competitive soccer players, training ≥5 days/week, ≥2 h/day, ≥1 year experience	Metabolomics	Urine	^{1}H NMR spectroscopy (600 MHz Varian Unity Inova) → Chenomx library for metabolite identification	Moderate
**Viciani et al., 2022 [69]**	Longitudinal observational study across 4 season phases	N= 38	18-37	Male	Elite professional soccer players (Serie A–level competitive season)	Metagenomics (16S rRNA gene sequencing)	Feces	16S rRNA sequencing; microbiota composition analysis; comparison across season phases	Moderate
**da Cruz et al., 2022 [70]**	Cross-sectional comparative study	N= 36	Elite: 18 ± 1 yrs; Non-elite: 20 ± 2 yrs	Male	U22 elite (National 1st division) vs non-elite (regional division)	Untargeted metabolomics (LC-MS)	Serum (fasted)	Liquid chromatography–mass spectrometry (Orbitrap Q Exactive Focus); XCMS preprocessing; PCA/PLS-DA; VIP score selection	High

**McAuley et al., 2022 [71]**	Cross-sectional genetic association study	N= 73	14.31 ± 2.16 years (U12–U18)	Male	English academy, Category 3	Candidate gene genotyping (10 psychogenetic SNPs)	Saliva / buccal swabs	DNA extraction (Qiagen on Kingfisher FLEX); custom Affymetrix Axiom microarray; standard QC (call rate, MAF, FLD); SNP–phenotype associations by linear regression; polygenic scores (TGS, TWGS)	Moderate
**Zileli et al., 2023 [72]**	Cross-sectional association study	N= 133	~23.6 ± 4 years	Male	Professional & regional amateur clubs	Candidate gene genotyping (ACTN3 R577X)	Buccal epithelial cells	DNA extraction with PureLink kit; Real-Time PCR using TaqMan genotyping assay (StepOnePlus);	Moderate
**Rodas et al., 2022 [73]**	Prospective observational longitudinal study	N= 51	Women: 25 ± 5 y; Men: 25 ± 5 y	Male & Female	Professional (FC Barcelona first teams)	Targeted metabolomics	Urine	Targeted UPLC-MS/MS analysis of amino acids + tryptophan & phenylalanine pathways; creatinine-normalized; multivariate models (PLS-DA, OPLS, overrepresentation analysis)	High
**Jacob et al., 2022 [74]**	Prospective longitudinal cohort study	N= 46	Adults	Male	Elite professional (AFL)	Genomics (candidate gene association)	Buccal epithelial cells (saliva swabs)	DNA extraction; genotyping performed by Agena Bioscience MassARRAY system; SNPs within ACTN3, CCL2, COL1A1, COL5A1, COL12A1, EMILIN1, IGF2,	Moderate

								NOGGIN, SMAD6	
**Végh et al., 2022[75]**	Cross-sectional experimental study with pre–post performance testing	118 total participants (64 elite male athletes: 41 football players + 23 endurance runners; 54 sedentary controls)	Athletes: 23.65 ± 3.46 y; Controls: 19.91 ± 0.66 y	Male	Elite / professional (football: 1st & 2nd Slovak leagues; endurance runners at national/international level)	Genomics (candidate gene polymorphisms)	Buccal epithelial cells (saliva swab)	PCR amplification + Sanger sequencing (Microsynth) for ACTN3, HIF1A, PPARA; PCR + gel electrophoresis for ACE indel (rs1799752); allele-specific sequencing verification	Moderate
**Varillas-Delgado et al., 2023 [76]**	Prospective cohort study (injury epidemiology across a full season)	N= 109	18-28	Male	High-performance national-league football (4 clubs, Spain)	Genomics (candidate SNP: CKM rs8111989)	Buccal epithelial cells (saliva smear)	DNA extraction via QIAcube; genotyping via single nucleotide primer extension (SNPE) using SNaPshot Multiplex Kit; fragment analysis via ABI3500 capillary electrophoresis	Moderate
**Papa et al., 2022 [77]**	Prospective cohort study	N=52	18-23	Male	Collegiate, NCAA Division I Football Bowl Subdivision	Proteomics (serum biomarker quantification)	Serum	Simoa (Quanterix) Neurology 4-plex assay on HD-X Analyzer (GFAP, UCH-L1, Tau, NF-L); samples run in duplicate; blinded lab; CV < 20%	High
**Coelho et al., 2016 [78]**	Cross-sectional genetic association study	N= 212	Adults	Male	Brazilian First Division (professional)	Genotyping	Blood leukocytes	PCR + RFLP analysis of ACE I/D polymorphism	Moderate
**Jacob et al., 2022 [79]**	Case–control genetic association study	47 elite AFL players +	Players: 24.0 ± 4.4 y; Controls:	Male	Elite (Australian Football League)	Genotyping	Saliva (buccal swab)	DNA extraction + genotyping performed at AGRF using	Moderate

		59 healthy controls	23.5 ± 3.1 y					standardized SNP assays	
**Kanope et al., 2021[80]**	Cross-sectional genetic association study	227 male players (U15 = 67; U17 = 43; U20 = 79; Professionals = 38)	U15: 15 ± 0.42 y; U17: 16 ± 0.84 y; U20: 19 ± 0.52 y; Pros: 24 ± 2.57 y	Male	Professional Brazilian club (youth + senior squads)	Genotyping	Whole blood (EDTA)	DNA extraction (proteinase K / salting-out) + TaqMan SNP genotyping assay (LIN28A rs6598964) + real-time PCR	Moderate
**Bulgay et al., 2023 [81]**	Within-subject pre–post training intervention	N= 22	18–35 y (24.79 ± 4.56 y)	Male	Professional (North Macedonian Super League)	Genotyping	Oral epithelial cells (buccal swab)	DNA extraction (Canvax kit) + Real-time PCR (StepOne Plus) + TaqMan SNP assays for AGT rs699 & IL-6 rs1800795	Moderate
**Hall et al., 2021[82]**	Prospective cohort genetic association study	402 Caucasian male academy soccer players (pre-PHV n = 101; post-PHV n = 301)	Pre-PHV: 11.5 ± 1.1 y; Post-PHV: 17.5 ± 2.1 y (overall 9–23 y)	Male	Elite academy players (England, Spain, Uruguay, Brazil; top-category professional club academies, U9–U23)	Genotyping	Saliva	Saliva collected into GeneFix tubes; genomic DNA isolated with PureLink Genomic DNA Mini Kit; SNP genotyping of 9 candidate variants via TaqMan assays using real-time PCR on Rotor-Gene Q; duplicate calls with 100% concordance; total genotype score (TGS) model for combined genetic risk	High
**McAuley et al., 2022 [83]**	Cross-sectional genetic comparison	N= 166	13-18 yrs	Male	English Category 1 & Category 3 academy players (Youth Development Phase: U12–U16; Professional	Genotyping (high-throughput SNV array)	Saliva (buccal swab)	DNA extracted via Qiagen chemistry on Kingfisher FLEX robot; DNA QC via PicoGreen & Nanodrop;	High

					Development Phase: U17–U23)			genotyping using custom Axiom SNP array; amplification/fragmentation on Biomek FXP; hybridization 24h at 48°C; array scanning on GeneTitan platform; SNV QC filters (call rate >95%, MAF >0.05)	
**Mohd Fazli et al., 2021 [84]**	Cross-sectional association study	N= 23	18.09 ± 0.42 y	Male	Elite U19 national-level Malaysian players	Genotyping (10 SNPs for power/endurance profiles)	Peripheral blood	DNA extracted by salting-out; SNP detection via allele-specific PCR (AS-PCR); PCR amplification with optimized cycling conditions; genotypes visualized via gel electrophoresis (3.5% agarose, RedSafe staining)	Moderate
**Rodas et al., 2021 [85]**	Prospective observational cohort (	N= 46	23-30 yrs	Male & Female	Top-level professional (LaLiga Santander & Primera Iberdrola)	Genotyping (single SNP: ACTN3 rs1815739)	Whole blood	DNA extraction with Qiagen QIAamp DNA Blood Mini Kit; genotyping via KASP™ allele-specific PCR	Moderate
**Marinho et al., 2021[86]**	Experimental observational study	N= 23	19 ± 1 y	Male	Elite U-20 (Brazilian Série A club)	Metabolomics (untargeted NMR)	Urine	^{1}H-NMR spectroscopy (Bruker 600 MHz AVANCE III) with presaturation; preprocessing in TopSpin® 3.2; metabolite identification via	Moderate

								Chenomx® and HMDB; multivariate analysis in MATLAB	
**Murtagh et al., 2020[87]**	Cross-sectional genotype–phenotype association study	686 total (535 elite soccer players, 151 controls)	ESP: 8–23 y; CON: 9–26 y (pre-/mid-/post-PHV groups; e.g. ESP pre-PHV 10.6 ± 1.4 y, post-PHV 16.8 ± 2.3 y)	Male	Elite academy youth (Category One in England and Category A in Uruguay) vs recreational/non-academy controls	Genomics (candidate SNP genotyping; 10 SNPs	Whole blood or saliva	DNA extracted with QIAamp DNA Blood Mini Kit; genotyping by real-time PCR (Rotor-Gene Q) using TaqMan SNP genotyping assays; duplicate genotyping with 100% concordance; SNPs selected in genes related to muscle structure, tendon, neuromuscular and metabolic function (e.g. ACTN3, AGT, AMPD1, BDNF, COL1A1/2/5A1, NOS3, PPARA, VDR)	High
**Wei, 2021 [88]**	Case–control genetic association study	260 total (60 elite female soccer athletes; 200 female controls)	Athletes: 23.8 ± 1.83 y; Controls: 18–25 y	Female	Elite professional female soccer players (Wuhan Jianghan & Jiangsu Suning clubs, China)	Genomics (candidate polymorphisms: ACE I/D; ACTN3 R577X)	Peripheral blood	Genotyping via PCR amplification; ACE I/D detected by gel electrophoresis (190 bp / 490 bp fragments); ACTN3 digested with DdeI followed by fragment analysis; some samples sequenced (ABI 3730 DNA Analyzer)	Moderate

**Clos et al., 2020[89]**	Cross-sectional genetic association study	N= 43	20-37 yrs	Male	Elite professional (single top-tier club)	Genomics (ACTN3 R577X polymorphism)	Whole blood	DNA extraction (Qiagen QIAamp), real-time PCR allelic discrimination using TaqMan assay (ABI Prism 7500); duplicate runs; standard QC	Low
**Muhan et al., 2023[90]**	Case–control genetic association study	20 football players + 76 sedentary controls	sedentary controls Players: 17–33 y (mean 22.2 ± 3.9)	Male	Amateur regional league (Maltepespor, Turkey)	Genotyping (SNP)	Peripheral blood	DNA extraction (PureLink kit), Real-Time PCR (TaqMan genotyping assay)	Low
**Suraci et al., 2021 [91]**	Mixed-model longitudinal training intervention	N= 30	17.2 ± 0.9 y	Male	Sub-elite U19 players competing nationally (English Football College Association)	Genotyping for multi-SNP TGS	Buccal swabs	DNA extraction (Gentra Puregene kit) + SNP genotyping via DNAFit's Peak Performance Algorithm (15-SNP weighted polygenic score)	Moderate
**Massidda et al., 2021 [92]**	Cross-sectional pilot genetic association study	N= 26	17.7 ± 0.78 yrs	Male	Elite Italian Primavera players (same club, official Italian Championship)	Candidate gene genotyping (MCT1 T1470A, rs1049434)	Buccal swabs for DNA; fingertip capillary blood for lactate (phenotype)	DNA extraction with QIAamp DNA Mini Kit; PCR amplification of MCT1 region; BccI restriction fragment length polymorphism (RFLP) to distinguish A/T alleles; genotype frequencies checked for HWE; association analyses under dominant model	Moderate

**Massidda et al., 2019 [93]**	Case–control + genotype–phenotype association; prospective injury follow-up (5 seasons)	257 total players (genotyping); 169 players followed for injuries; 265 sedentary controls	Players: 21.2 ± 5.3 y (full sample); Injury subgroup: 19.4 ± 5.2 y	Male	Professional (Serie A, Primavera, Allievi, Giovanissimi – Italian national championship)	Candidate gene genotyping (ACTN3 R577X)	Buccal swab DNA	PCR amplification of ACTN3 exon 16; DdeI restriction digest; polyacrylamide gel electrophoresis; genotype–phenotype association with ORs for incidence & severity	High
**Pickering et al., 2019 [94]**	Genome-wide association study (GWAS) + candidate gene analysis + multi-cohort validation	48 elite youth football players (British cohort) for GWAS; replication in 126 Polish women; validation in 176 elite Russian power athletes + 223 endurance athletes + 173 controls; muscle biopsy cohort n=96	British cohort: 12–18 years	Male	Premier League Youth Academy (elite youth)	Genome-wide genotyping (>600K–1.14M SNPs) + candidate SNP genotyping	Saliva DNA (British & Polish); blood DNA (Russian cohorts)	Affymetrix Axiom microarrays (British/Polish); Illumina HumanOmni arrays (Russian); QC filtering (PCA for ethnicity); validation across multiple populations; muscle fiber typing via immunohistochemistry	High
**Petrovic et al., 2022 [95]**	Cross-sectional candidate-gene association study	45 elite female football players (FG) + 60 sedentary female controls (CG)	FG: 17.8 ± 1.2 years; CG: 35.2 ± 7.9 years	Female	Young elite Serbian female footballers	Genomics (candidate gene) – ACE I/D (rs1799752) and ACTN3 R577X (rs1815739)	Buccal swabs (buccal epithelial cells)	DNA extracted with PureLink Genomic DNA kit; high-resolution melting (HRM) PCR on ViiA 7 (MeltDoctor HRM Master Mix); HRM calls	Moderate

								validated against conventional genotyping (gel electrophoresis for ACE I/D; RFLP for ACTN3 R577X) with 100% concordance	
**Hunter et al., 2020 [96]**	Cross-sectional analysis using longitudinal cohort data	N= 352	Median 23 y	Male & Female	Amateur / recreational, ≥5 years playing, ≥6 months/year	Genotyping of APOE ε4 (rs429358, rs7412)	Whole blood	APOE genotyping: Global Screening Array-24 v1.0 + TaqMan SNP genotyping; QC filtering (>95% call rate); neurocognitive assessment via CogState (ISL Delayed Recall)	Moderate
**Pitti et al., 2019[97]**	Observational pre–post study of an official match using untargeted metabolomics	N= 17	NR	Female	Professional club (Italian women's top level; Coppa Italia match)	Untargeted ^{1}H NMR-based metabolomics	Unstimulated whole saliva	Bruker Avance 600 MHz, NOESYPR1D ^{1}H-NMR; deproteinization by ultrafiltration; metabolite ID & quantification with Chenomx NMR Suite; total protein via Bradford assay; computed TPWS & TOMC; multivariate PCA (SIMCA-P), probabilistic quotient normalization and protein normalization, volcano plots for univariate analysis	Moderate

**Muraoka et al., 2019 [98]**	Cross-sectional case–control proteomic study of CSF-derived EVs	15 former NFL players with cognitive/neuropsychiatric symptoms + 16 age-matched male controls without contact sports/TBI (total n = 31)	NFL= 56.33 ± 7.31 , Ctr= 57.06 ± 6.95	Male	Former National Football League (retired pros)	Label-based quantitative proteomics (TMT-10plex) + ultrasensitive tau immunoassays	Cerebrospinal fluid–derived extracellular vesicles (CSF EVs)	EV isolation from CSF via MagCapture Exosome Isolation Kit PS after differential centrifugation and 0.22 µm filtration; characterization by NTA; EV lysis and t-tau/p-tau181 measured with Simoa HD-1 (Quanterix); protein precipitation (TCA), in-gel trypsin digestion; TMT 10-plex labeling; nanoLC-MS/MS on Orbitrap Fusion Lumos; SEQUEST search vs UniProt human, 1% peptide & protein FDR via LDA; reporter ion–based quantification; downstream GO (DAVID) and Ingenuity Pathway Analysis	Moderate
**Massidda et al., 2020 [99]**	Multicenter genetic association study (retrospective cohort / case–control) + meta-analysis across two elite cohorts	N= 710	Italians: 19.9 ± 5.0 years; Japanese: 20.8 ± 1.4 years	Male	Italian Official National Football Championship (elite pro & academy) + Japanese national/international-level elite players	Candidate gene genotyping (ACE I/D, rs1799752 via proxy rs4341 in Japanese cohort)	Buccal epithelial DNA (Italian cohort); saliva DNA (Japanese cohort)	Italian: PCR amplification of ACE I/D with specific primers, agarose gel electrophoresis to distinguish DD/ID/II; Japanese: TaqMan SNP genotyping of rs4341 (perfect LD	Moderate

								with ACE I/D) on LightCycler 480;	
**Jacob et al., 2021[100]**	Candidate gene association study	N= 46	NR	Male	Australian Football League (AFL), elite professional	Targeted genotyping of four candidate SNPs	Saliva buccal swabs	DNA extraction + genotyping performed by Australian Genome Research Facility using Agena Bioscience MassARRAY system; GLMM for repeated-measure association with 2-km performance	Moderate
**Alzharani et al., 2020 [101]**	Pre–post untargeted metabolomics study	N= 26	20.6 ± 1.4 yrs	Male	Young professional football players in Saudi Arabia (mid-season; some U23 national team members)	Untargeted LC–MS–based metabolomics	Plasma, urine, saliva	Accela HPLC + Orbitrap Exactive MS; ZIC-pHILIC column for polar metabolites (all biofluids) and ACE C4 RP column for non-polar plasma metabolites; ESI positive/negative switching; data processed with Xcalibur and m/zMine 2.14; feature filtering by RSD and $p<0.05$; multivariate analysis with SIMCA-P 14.1 (PCA, OPLS-DA); pooled QC samples and internal standard ($^{13}C_2$-glycine)	Moderate
**Cao et al., 2020[102]**	Pre–post high-intensity combined	N= 12	Teenage; exact age	Male	Teenage competitive football players from Shaanxi Sports School	Untargeted GC–MS–based metabolomics	Urine	Agilent 7890 GC–TOF-MS with DB-5MS capillary	Moderate

	aerobic + anaerobic cycling protocol to exercise-induced fatigue with urinary metabolomics		not reported					column; urease treatment, MeOH extraction, methoxyamine + BSTFA derivatization; splitless injection; full scan 50–500 m/z; data processed with Chroma TOF 4.3X (peak picking, deconvolution, alignment, internal-standard normalization); multivariate analysis in SIMCA v14 (PCA, OPLS-DA); metabolite ID via KEGG, pathway analysis via MetPA; differential metabolites selected by VIP>1 & p<0.05	
**Larruskain et al., 2018[103]**	Prospective cohort + genetic association study	N= 107	≥18 yrs	Male	Elite soccer (La Liga First Team, Reserves, U19; Athletic Club, Spain)	Candidate SNP genotyping (37 SNPs)	Saliva (buccal swabs)	DNA extraction (QIAmp), quantification (Qubit), SNP-type assays on Fluidigm Biomark HD; multivariable Cox frailty model; validation using Harrell's C-index	Moderate
**Clos et al., 2019 [104]**	Prospective cohort + genetic association study	N= 43	Adults	Male	Elite professional football (Spanish First Division club)	Candidate SNP genotyping (ACTN3 R577X)	Whole blood	DNA extraction (QIAmp Blood Minikit), quantification (NanoDrop), TaqMan allelic	Moderate

								discrimination assay on ABI Prism 7500; PCR run in duplicate with controls	
**Coelho et al., 2019 [105]**	Experimental study	N= 30	U-16; <16 years old	Male	Elite youth players (Brazilian first-division club academy)	Candidate SNP genotyping (ACTN3 R577X)	Peripheral blood	DNA extraction (proteinase K + salt precipitation), PCR amplification of exon 16, DdeI restriction digestion, polyacrylamide gel electrophoresis; Biochemical assays: CK (enzymatic), α-actin (ELISA), IL-6 (ELISA), cortisol & testosterone (chemiluminescence)	Moderate
**Koku et al., 2019 [106]**	Cross-sectional case–control	N= 201	18-30 yrs	Male	Amateur club players (Ege region, Turkey)	Candidate SNP genotyping (ACTN3 R577X)	Venous blood	DNA isolation (High Pure PCR Template kit, Roche); ACTN3 R577X genotyping by real-time PCR (LightCycler 480); performance: standing long jump, countermovement jumps (with/without arm swing, repeated), VO_2max by treadmill CPX (Masterscreen CPX)	Moderate
**Pruna et al., 2017 [107]**	Cross-sectional genetic	N= 74	Adults	Male	Elite professional	Candidate SNP genotyping (12 SNPs in	Genomic DNA (likely blood, not	Allelic discrimination assays (real-time	Moderate

	association study					HGF, SOX15, GEFT, LIF, etc.)	explicitly specified)	PCR–based SNP genotyping); association tests between genotypes and injury outcomes	
**Dionísio et al., 2017 [108]**	Cross-sectional genetic association study	N= 220	Young athletes	Male	Professional minor league (São Paulo FC Academy)	Candidate SNP genotyping (ACTN3 R577X, AMPD1 C34T, ACE I/D, AGT M235T)	Saliva DNA	TaqMan allelic discrimination assays	Moderate
**Coelho et al., 2018 [109]**	Cross-sectional comparative genetic association study	353 football players + 100 controls	U14 → PRO (14–23+ years)	Male	First-division Brazilian clubs (U14, U15, U17, U20, Professional)	Candidate SNP genotyping (ACTN3 R577X)	Blood DNA	PCR–RFLP with DdeI digestion + electrophoresis	Moderate
**Jacob et al., 2019 [110]**	Prospective observational genetic association pilot study	N= 30	16–18 years (mean 17.0 ± 0.76)	Male	Sub-elite / semi-professional – Western Australian Football League (WAFL) junior squad from a single club	Candidate gene SNP genotyping (9 polymorphisms): ACE (rs4343), ACTN3 (rs1815739), ADRB1 (rs1801253), ADRB2 (rs1042714), ADRB3 (rs4994), BDNF (rs6265), COMT (rs4680), DRD2 (rs1076560), PPARGC1A (rs8192678)	Whole blood	DNA extraction at AGRF; Sequenom MassARRAY custom genotyping panel; Hardy–Weinberg check; performance/specific-skill tests; match stats from	Moderate
**Atabaş et al., 2020 [111]**	Cross-sectional candidate-gene association study	19 male soccer players + 9 sedentary adults (controls)	18-25 yrs	Male	Amateur	Candidate SNP genotyping (ACTN3 R577X, ACE I/D, UCP3 -55C/T)	Blood (venous) DNA	PCR for ACE I/D + agarose gel electrophoresis; PCR + automated DNA sequencing (ABI Prism) for ACTN3 & UCP3 genotypes	Low
**Prado et al., 2017 [112]**	Non-targeted metabolomics observational study	N= 30	18–20 years	Male	Semi-professional (CBF-affiliated team, Brazil)	Non-targeted metabolomics (UPLC-MSE mass spectrometry)	Urine (primary), Blood (subset of 7 players for point-of-care comparison)	UPLC-MSE (Xevo G2-S Q-ToF) + Progenesis QI data processing; HMDB & KEGG identification;	High

								SMPDB pathway classification	
**Domańska-Senderowska et al., 2019 [113]**	Longitudinal pre–post gene expression study	N= 22	17-18 yrs	Male	Competitive youth soccer (Poland)	Gene expression analysis (ACTN3 mRNA)	Peripheral blood lymphocytes	RNA isolation (mirVana kit) → Bioanalyzer QC → cDNA synthesis → qPCR (TaqMan probes on Applied Biosystems 7900HT)	Moderate
**Massidda et al., 2018 [114]**	Multicenter case–control genotype–phenotype association study	N= 694	Adults	Male	Top-level/professional national championships: Italy (Serie A–D), Poland (Ekstraklasa), Lithuania National Team, Ukraine Premier League, Malta (merged with Italian sample)	Candidate SNP genotyping (MCT1 A1470T, rs1049434)	Buccal epithelial cells and peripheral blood leukocytes (genomic DNA)	PCR-based genotyping: buccal/blood DNA extraction → PCR amplification of MCT1 exon region → restriction digest (e.g., BccI) and fragment analysis in some labs; TaqMan allelic discrimination real-time PCR assays (Applied Biosystems) in Lithuanian and Ukrainian cohorts	Moderate
**Coelho et al., 2015 [115]**	Cross-sectional genetic association study	N= 138	U17, U20, Adults	Male	Brazilian first-division clubs; categories: U17, U20, adult professional	Candidate SNP genotyping (ACTN3 R577X)	Candidate SNP genotyping (ACTN3 R577X)	PCR-based ACTN3 genotyping (R577X), method consistent with earlier Coelho/Oliveira publications (PCR + restriction digest or sequencing)	Moderate
**Jeremic et al., 2019 [116]**	Cross-sectional genotype–phenotype association study	N= 27	16-18 yrs	Female	Serbian national U18 team	Candidate SNP genotyping (ACE I/D, ACTN3 R577X)	Buccal cells	HRM-PCR (High-Resolution Melting PCR) using an in-house validated method	Moderate

**Galeandro et al., 2017 [117]**	Case–control genetic association study	43professional soccer players + 128 non-athletic controls	Players: 25 ± 6 y; Controls: 40 ± 15 y	Male (players); mixed in controls	Italian 2nd Division professional team	Candidate SNP genotyping (ACTN3 R577X, ACE I/D) + mtDNA copy-number quantification	Peripheral blood	PCR–RFLP for ACTN3/ACE genotyping; qPCR (RT-PCR) for mitochondrial DNA copy number	Moderate
**Massidda et al., 2015 [118]**	Prospective cohort + genetic association study	N= 54	25.9 ± 4.3 yrs	Male	Elite Italian Professional Championship (4 consecutive seasons)	Candidate SNP genotyping (COL5A1 rs12722)	Buccal swab DNA	PCR + BstUI restriction enzyme digestion	Moderate
**Sun et al., 2023 [119]**	Case–control genetic association study	N= 430	ACLR males 26 ± 4; ACLR females 26 ± 6; Controls: males 26 ± 6, females 29 ± 2	Male + Female	Polish soccer leagues (1st–3rd divisions); physically active controls	Candidate SNP genotyping (COL22A1 rs11784270 and rs6577958)	Buccal swab	TaqMan® SNP genotyping assays (RT-PCR on StepOne system)	High
**Massidda et al., 2016 [120]**	Cross-sectional genetic association study	N= 128	16.3 ± 1.3 years (range 14.3–21.1)	Male	Young elite Italian soccer players, National-level championship	Candidate SNP genotyping (MCT1 A1470T, rs1049434)	Saliva / buccal swab	PCR + BccI restriction digestion; fragment discrimination by electrophoresis	Moderate
**Hunter et al., 2020 [121]**	Longitudinal cohort study with gene–environment interaction analysis	312 amateur soccer players + 110 healthy controls	18–55 years	Male + Female	Adult amateur soccer (NYC leagues, clubs, colleges)	Genome-wide genotyping array (BDNF Val66Met, rs6265)	Whole blood (venipuncture)	Global Screening Array-24 v1.0 (Illumina) + Golden Helix SVS QC pipeline	High
**Jacob et al., 2020 [122]**	Cross-sectional pilot genetic association study	N= 30	Young adults	Male	Sub-elite Australian Football	Candidate gene SNP genotyping (9 polymorphisms)	Buccal swab DNA	PCR-based SNP genotyping (platform not explicitly described; likely HRM-PCR or TaqMan assays common in this lab's work)	Low
**Coelho et al., 2016 [123]**	Cross-sectional genetic frequency study	N= 453	U14–PRO (14–23+ years)	Male	First-division Brazilian clubs	Candidate SNP genotyping (ACE I/D, rs4646994)	Blood DNA	PCR for I/D fragment (319 bp D allele, 597 bp I	Moderate

								allele) + confirmatory I-specific PCR; polyacrylamide gel electrophoresis + silver staining	
**Ra et al., 2014 [124]**	Observational cohort study	N= 122	~20.6 years	Male	Intercollegiate competitive soccer team (Japan)	Metabolomics (targeted & untargeted)	Saliva	Capillary electrophoresis–time-of-flight mass spectrometry (CE-TOFMS); multivariate PCA; internal standards normalization	Moderate
**Dinç et al., 2017 [125]**	Cross-sectional comparative genetic association study	N= 96	18-27 yrs	Male	Turkish Super League & Major League (A2 team level)	Candidate SNP genotyping (MTHFR C677T)	Whole blood	PCR amplification of MTHFR C677T variant; biochemical assays for Hcy, folate, lipids, hemogram	Moderate
**Egorova et al., 2014 [126]**	Case–control genetic association study	246 football players + 872 non-athlete controls	Elite: 23.9 ± 0.6 y; Sub-elite: 23.0 ± 0.7 y; Youth/non-elite: 10.6 ± 0.1 y; Controls: 19.8 ± 0.2 y	Male	Elite: Russian Premier League; Sub-elite: Russian Football National League & Russian Second Division; Non-elite: youth players from Premier League clubs	Candidate SNP genotyping (8 performance-related polymorphisms: ACE I/D, ACTN3 R577X, PPARA rs4253778, PPARG rs1801282, PPARGC1A rs8192678, PPARD rs2016520, TFAM rs1937, UCP2 rs660339)	Buccal epithelial cells / mouth swabs	DNA extraction (alkaline or DNK-sorb kit); PCR on multichannel Tercyk thermocycler + restriction enzyme digestion (PCR–RFLP) for each SNP; genotyping performed blinded; polygenic "Total Genotype Score (TGS)" calculated (0–100) based on carriage of "favourable" alleles	Moderate
**Pruna et al., 2013 [127]**	Prospective observational cohort + genetic association	N= 73	Adults	Male	Elite European football players (FC Barcelona)	Candidate SNP genotyping (8 SNPs: ELN, TTN, SOX15, IGF2, CCL2, COL1A1, COL5A1, TNC)	Venous whole blood	DNA extraction (Qiagen QIAamp); genotyping using TaqMan allelic discrimination	Moderate

								assays (real-time PCR); SNP–injury correlations tested via Chi-square/Fisher	
**Pimenta et al., 2013 [128]**	Cross-sectional genetic association study	N= 200	Adults	Male	Brazilian First Division professional teams	Candidate SNP genotyping (ACTN3 R577X)	Peripheral venous blood	DNA extraction (proteinase K + salt precipitation); PCR amplification of exon 16; restriction fragment length polymorphism (PCR–RFLP) with DdeI digestion; polyacrylamide gel electrophoresis + silver staining	Moderate
**Ginevičienė et al., 2014 [129]**	Case–control genetic association study	199 Lithuanian professional male footballers and 167 sedentary male controls	Footballers: 17–20 yrs; Controls: 18–22 yrs	Male	Lithuanian “sub-elite” professional players (national championships + international matches)	Candidate SNP genotyping (ACE I/D, PPARGC1A Gly482Ser (G/A), PPARA intron 7 G/C	Peripheral blood leukocytes	Genomic DNA extracted by phenol–chloroform; ACE I/D genotyped by PCR (Tiret method) with size discrimination (190 bp D allele, 490 bp I allele); PPARGC1A rs8192678 and PPARA rs4253778 genotyped by PCR + restriction fragment length polymorphism (PCR–RFLP): MspI digestion for PPARGC1A (449 bp G; 274+175 bp A); TaqI digestion for PPARA (266 bp, cleaved to 216+50 bp for C allele);	Moderate

**La Montagna et al., 2019 [130]**	Cross-sectional genomic association study	N= 30	Adults	Male	Italian Serie A, elite professional team	Candidate SNP genotyping (ACTN3 R577X, COL5A1 rs12722, MCT1, VEGF –634C>G, HFE C282Y/H63D/S65C)	Saliva (Oragene OG-500 collection kit)	Combination of techniques : PCR + Sanger sequencing for MCT1 – TaqMan allelic discrimination assays (Real-time PCR) for ACTN3, COL5A1, HFE, VEGF Thermocycling on Roche LightCycler 480II; fragment confirmation on agarose gels	Moderate
**Ficek et al., 2013 [131]**	Case–control genetic association study	91 ACL-ruptured professional soccer players + 143 healthy professional soccer player controls	ACL group: 23 ± 3 y; Controls: 25.2 ± 2.6 y	Male	Polish 1st Division professional players	Candidate SNP genotyping (COL1A1 −1997G/T, +1245G/T)	Buccal epithelial cells (oral swabs)	TaqMan SNP Genotyping Assays (Applied Biosystems) + Real-time PCR on Rotor-Gene platform; allele discrimination using fluorescent probes; haplotype analysis using R haplo.stats	High
**Pruna et al., 2013 [132]**	Observational cohort study with genetic association analysis	73 elite professional soccer players with 242 non-contact soft tissue injuries	Adults	Male	FC Barcelona – First and Second Team	Candidate SNP genotyping (8 genes: ELN, TTN, SOX15, IGF2, CCL2, COL1A1, COL5A1, TNC)	Whole blood	TaqMan allelic discrimination assays on ABI 7500 real-time PCR system; duplicate runs for all samples	Moderate
**Eynon et al., 2012 [133]**	Cross-sectional comparative genetic association study	60 elite soccer players, 100 world-class endurance	Soccer players: 17–32 y; Endurance: 20–39 y;	Male	Professional Spanish soccer team ranked top by FIFA; Olympic-level endurance and power athletes;	Genotyping of candidate SNP (NOS3 −786 T/C, rs2070744)	Blood DNA	Soccer players genotyped via PCR + NgoMI restriction digestion; other	High

		athletes, 53 elite power athletes, 100 sedentary controls	Power: 20–33 y; Controls: 19–32 y		university student controls			groups genotyped via low-density DNA microarray (allele-specific probes) with automated hybridization/scanning	
**Massidda et al., 2012 [134]**	Cross-sectional genetic association study	42 elite Italian soccer players, 106 sedentary healthy Italian controls	Adults	Male	Professional Italian top-level soccer players	Candidate-gene SNP genotyping (ACE I/D, ACTN3 R577X, BDKRB2 +9/−9)	buccal DNA	Standard PCR-based genotyping	Low
**Micheli et al., 2011[135]**	Cross-sectional genetic association study	125 soccer players (young competitive) + sedentary controls from literature	<17 years (U17 category)	Male	Medium–high-level Italian youth soccer players (federation-selected; FIGC Coverciano)	Candidate SNP genotyping (ACE I/D, VDR FokI)	Hair-derived genomic DNA	PCR amplification, restriction enzyme digestion (FokI for VDR; allele-specific PCR for ACE; insertion-specific confirmation PCR for DD), agarose gel electrophoresis	Moderate
**Juffer et al., 2009 [136]**	Cross-sectional comparative genetic association study	54 elite soccer players, 52 elite endurance runners, 123 sedentary controls	Soccer players: 18–32 y; Runners: 19–38 y; Controls: 19–50 y	Male	Top-level European soccer club (Spanish 1st & 2nd division; several Champions League winners; elite runners including Olympians)	Candidate SNP genotyping (ACE I/D, GDF-8 K153R/E164K/P198A/I225T, AMPD1 C34T)	Blood-derived genomic DNA	PCR + gel electrophoresis (ACE), PCR + single base extension + ABI Prism 310 (GDF-8), TaqMan real-time PCR (AMPD1); ACE genotyping externally validated via microarray	High
**Martín-Sánchez et al., 2013 [137]**	Cross-sectional comparative proteomic study	12 professional soccer players + 9	Professionals: 25 ± 4 y;	Male	Spanish Primera División professional team; recreational	Proteomics (2-DE + MALDI-TOF MS)	Plasma	2-DE (IEF + SDS-PAGE) → silver staining → spot excision	Moderate

		recreational players (controls)	Recreational: 26 ± 3 y		adults practicing 3–4 h/week			→ trypsin digestion → MALDI-TOF MS → Mascot database identification. Additional assays: ELISA (IL-6, sICAM-1)	
**Atli, 2013 [138]**	Cross-sectional comparative biochemical study	23 adult football players + 23 sedentary controls	Not precisely reported; adults	Male	University-level adult football players (Yuzuncu Yil University, Turkey)	Biochemical enzymatic assays (PON1 activity, arylesterase, LOOH)	Serum	Commercial enzymatic kits (Relassay) for paraoxonase and arylesterase activities; FOX assay for lipid hydroperoxides; Standard clinical chemistry analyzer for lipids	Low
**Rodas et al., 2023 [139]**	Cohort genetic association study (sex-stratified)	N= 46	Adults	Male & Female	FC Barcelona first team (LaLiga & Primera Iberdrola)	Candidate SNP genotyping (108 SNPs — collagen, muscle, tendon genes)	Blood (EDTA)	KASP genotyping (Kompetitive Allele Specific PCR) + QC (HWE, call rate); haplotype inference (haplo.stats)	High
**Ang et al., 2024 [140]**	Causal-comparative intervention study	N= 20	18.7 ± 0.6 years	Male	Professional club footballers	Candidate SNP genotyping (ACE I/D rs1799752, ACTN3 R577X rs1815739)	Peripheral blood DNA	Conventional PCR with specific primers → agarose gel electrophoresis; ACE DD genotypes re-checked with insertion-specific PCR	Moderate

References

1. de Almeida, K.Y.; Dos Santos Guimarães, R.; Rocha, M.R.; Saito, M.; Kikuchi, N. ACTN3 R577X Polymorphism and Muscle Injury Severity among Professional Brazilian Soccer Players: A Heightened Impact on Female Athletes. *Gazz. Med. Ital. Arch. Sci. Med.* **2025**, *184*, 163–170, doi:10.23736/S0393-3660.24.05572-4.

2. Aslan, B.T.; Polat, T.; Yılmaz, Ö.Ö.; Muhan, A.; Ziya, R.; Ulucan, K. Comparison of MCM6 Rs4988235 Polymorphism Allele and Genotype Frequencies in Professional Football Players and a Sedentary Control Group. *SAUJS* **2024**, *28*, 1040–1046.

3. Jówko, E.; Długołęcka, B.; Cieśliński, I.; Kotowska, J. Polymorphisms in Genes Encoding VDR, CALCR and Antioxidant Enzymes as Predictors of Bone Tissue Condition in Young, Healthy Men. *Int. J. Mol. Sci.* **2023**, *24*, doi:10.3390/ijms24043373.

4. Center for the development of professional qualifications of medical workers; Kayumov, A.M.; Khamrabayeva, F.I.; Center for the development of professional qualifications of medical workers; Yunusova, L.R.; Tashkent State Dental Institute ANALYSIS OF PHYSICAL PERFORMANCE OF FOOTBALL PLAYERS CONSIDERING POLYMORPHISM OF ENDURANCE GENES. *BCCM* **2022**, *15*, 40–43, doi:10.20969/VSKM.2022.15(6).40-43.

5. Nunes, M.J.; Cordas, C.M.; Moura, J.J.G.; Noronha, J.P.; Branco, L.C. Screening of Potential Stress Biomarkers in Sweat Associated with Sports Training. *Sports Medicine - Open* **2021**, *7*, 8, doi:10.1186/s40798-020-00294-3.

6. Kawata, K.; Steinfeldt, J.A.; Huibregtse, M.E.; Nowak, M.K.; Macy, J.T.; Kercher, K.; Rettke, D.J.; Shin, A.; Chen, Z.; Ejima, K.; et al. Association Between Proteomic Blood Biomarkers and DTI/NODDI Metrics in Adolescent Football Players: A Pilot Study. *Front Neurol* **2020**, *11*, 581781, doi:10.3389/fneur.2020.581781.

7. Subak, E.; Muniroglu, S. Sox15 t/g May Be a Candidate Gene Polymorphism for Athlete Performance. *J. Phys. Educ. Sport* **2020**, *20*, 2614–2619, doi:10.7752/jpes.2020.05356.

8. Hassan, E.A.; Shady, A.A. Significance of Actn3 Gene and Its Relation with Physiological and Physical Variables as a Genetic Marker for Soccer Players' Selection at High Altitudes. *J. Phys. Educ. Sport* **2020**, *20*, 1711–1720, doi:10.7752/jpes.2020.04232.

9. Ük, Y.; Kapıcı, S.; Yüksel, İ.; Sercan, C.; Eken, B.F.; Ulucan, K. Peroxisome Proliferator-Activated Receptor Alpha (PPARα) Rs4253778 Polymorphism in a Turkish Soccer Player Cohort. *Pamukkale. J. Sport. Sci.* **2020**, *11*, 1–6.

10. Ioffe, O.Y.; Omelchenko, A.V.; Goncharov, S.V.; Stroy, D.O.; Drozdovska, S.B.; Stetsenko, O.P.; Kryvopustov, M.S.; Dibrova, Y.A.; Galyga, T.M.; Vasilyev, A.V.; et al. Association Analysis of Gene Polymorphisms Col1a, Mct1, Col12a1 with Sports Hernia in Football Players. *Fiziol. Zh.* **2020**, *66*, 33–40, doi:10.15407/fz66.06.033.

11. Cocci, P.; School of Biosciences and Veterinary Medicine, University of Camerino, Via Gentile III Da Varano, I-62032 Camerino (MC), Italy; Pistolesi, L.; School of Biosciences and Veterinary Medicine, University of Camerino, Via Gentile III Da Varano, I-62032 Camerino (MC), Italy; Guercioni, M.; School of Biosciences and Veterinary Medicine, University of Camerino, Via Gentile III Da Varano, I-62032 Camerino (MC), Italy; Belli, L.; School of Biosciences and Veterinary Medicine, University of Camerino, Via Gentile III Da Varano, I-62032 Camerino (MC), Italy; Carli, D.; Athletic Thai Gym, Via Sebastiano Vanzi, 16 47900 Rimini (RN); et al. Genetic Variants and Mixed Sport Disciplines: A Comparison among Soccer, Combat and Motorcycle Athletes. *Ann. Appl. Sport Sci* **2019**, *7*, 1–9, doi:10.29252/aassjournal.7.1.1.

12. Honarpour, A.; Mohseni, M.; Hajiagha, S.G.; Irani, S.; Najmabadi, H. Investigation of the Relationship between a Genetic Polymorphism in ACTN3 and Elite Sport Performance among Iranian Soccer Players. *Iran. Rehabil. J.* **2017**, *15*, 149–154, doi:10.18869/nrip.irj.15.2.149.

13. Kenger, E.B.; Eren, F.; Ozlu, T.; Gunes, F.E. Analysis of Microbiota Profile and Nutritional Status in Male Professional Football Players. *J Sports Med Phys Fitness* **2023**, *63*, 1235–1243, doi:10.23736/S0022-4707.23.15103-6.

14. Pintus, R.; Bongiovanni, T.; Corbu, S.; Francavilla, V.C.; DessÌ, A.; Noto, A.; Corsello, G.; Finco, G.; Fanos, V.; Cesare Marincola, F. Sportomics in Professional Soccer Players: Metabolomics Results during Preseason. *J Sports Med Phys Fitness* **2021**, *61*, 324–330, doi:10.23736/S0022-4707.20.11200-3.

15. Cięszczyk, P.; Leońska-Duniec, A.; Maciejewska-Skrendo, A.; Sawczuk, M.; Leźnickanicka, K.; Contrò, V.; Trybek, G.; Lulińska-Kuklik, E. Variation in the Ace Gene in Elite Polish Football Players. *Hum. Mov.* **2016**, *17*, 237–241, doi:10.1515/humo-2016-0032.

16. Sport & Exercise Medicine SwitzerlandRabbentalstrasse 83 3013 Berne Switzerland Phone: +41 31 333 02 54 E-Mail www. sems.ch Genetic polymorphisms in alpha-actinin 3 and -adrenoceptor beta genes in Austrian elite athletes and healthy controls Available online: https://sems-journal.ch/4565 (accessed on 26 November 2025).

17. Massidda, M.; Eynon, N.; Bachis, V.; Corrias, L.; Culigioni, C.; Piras, F.; Cugia, P.; Scorcu, M.; Calò, C.M. Influence of the MCT1 Rs1049434 on Indirect Muscle Disorders/Injuries in Elite Football Players. *Sports Med Open* **2015**, *1*, 33, doi:10.1186/s40798-015-0033-9.

18. Marinich, V.V.; Guba, V.P.; Mizernitskiy, Y.L. Evaluation of the psychophysiological state of young sportsmen depending on polymorphism of L/S alleles of the 5HTT gene and C/T alleles of the 5HT2A gene. *Vopr. Prakt. Pediatr.* **2013**, *8*, 8–13.

19. Massidda, M.; Corrias, L.; Scorcu, M.; Vona, G.; Caló, M. ACTN-3 and ACE Genotypes in Elite Male Italian Athletes. *Anthropological Review* **2013**, *75*, 55–59, doi:10.2478/v10044-012-0004-4.

20. Pimenta, E.M.; Coelho, D.B.; Cruz, I.R.; Morandi, R.F.; Veneroso, C.E.; De Azambuja Pussieldi, G.; Carvalho, M.R.S.; Silami-Garcia, E.; De Paz Fernández, J.A. The ACTN3 Genotype in Soccer Players in Response to Acute Eccentric Training. *Eur. J. Appl. Physiol.* **2012**, *112*, 1495–1503, doi:10.1007/s00421-011-2109-7.

21. Kambouris, M.; Del Buono, A.; Maffulli, N. Genomics DNA Profiling in Elite Professional Soccer Players: A Pilot Study. *Transl Med UniSa* **2014**, *9*, 18–22.

22. Proia, P.; Bianco, A.; Schiera, G.; Saladino, P.; Contrò, V.; Caramazza, G.; Traina, M.; Grimaldi, K.A.; Palma, A.; Paoli, A. PPARα Gene Variants as Predicted Performance-Enhancing Polymorphisms in Professional Italian Soccer Players. *Open Access J Sports Med* **2014**, *5*, 273–278, doi:10.2147/OAJSM.S68333.

23. Orrù, S.; Imperlini, E.; Vitucci, D.; Caterino, M.; Mandola, A.; Randers, M.B.; Schmidt, J.F.; Hagman, M.; Andersen, T.R.; Krustrup, P.; et al. Insight into the Molecular Signature of Skeletal Muscle Characterizing Lifelong Football Players. *Int J Environ Res Public Health* **2022**, *19*, 15835, doi:10.3390/ijerph192315835.

24. Ulucan, K.; Sercan, C.; Biyikli, T. Distribution of Angiotensin-1 Converting Enzyme Insertion/Deletion and α-Actinin-3 Codon 577 Polymorphisms in Turkish Male Soccer Players. *Genet Epigenet* **2015**, *7*, 1–4, doi:10.4137/GEG.S31479.

25. Yang, S.; Zhang, W.; Jia, M.; Chen, H. Association between Vitamin D Receptor Gene Polymorphisms and Athletic Performance in Chinese Male Youth Soccer Players. *PeerJ* **2025**, *13*, e19696, doi:10.7717/peerj.19696.

26. Contrò, V.; Schiera, G.; Abbruzzo, A.; Bianco, A.; Amato, A.; Sacco, A.; Macchiarella, A.; Palma, A.; Proia, P. An Innovative Way to Highlight the Power of Each Polymorphism on Elite Athletes Phenotype Expression. *Eur J Transl Myol* **2018**, *28*, 7186, doi:10.4081/ejtm.2018.7186.

27. Artells, R.; Pruna, R.; Dellal, A.; Maffulli, N. Elastin: A Possible Genetic Biomarker for More Severe Ligament Injuries in Elite Soccer. A Pilot Study. *Muscles Ligaments Tendons J* **2016**, *6*, 188–192, doi:10.11138/mltj/2016.6.2.188.

28. Sha, J.-B.; Zhang, S.-S.; Lu, Y.-M.; Gong, W.-J.; Jiang, X.-P.; Wang, J.-J.; Qiao, T.-L.; Zhang, H.-H.; Zhao, M.-Q.; Wang, D.-P.; et al. Effects of the Long-Term Consumption of Hydrogen-Rich Water on the Antioxidant Activity and the Gut Flora in Female Juvenile Soccer Players from Suzhou, China. *Med Gas Res* **2018**, *8*, 135–143, doi:10.4103/2045-9912.248263.

29. Lifanov, D.; Khadyeva, M.N.; Rahmatullina, L.S.; Demenev, S.V.; Ibragimov, R.R. Effect of Creatine Supplementation on Physical Performance Are Related to the AMPD1 and PPARG Genes Polymorphisms in Football Players. *Ross Fiziol Zh Im I M Sechenova* **2014**, *100*, 767–776.

30. González, J.R.; Cáceres, A.; Ferrer, E.; Balagué-Dobón, L.; Escribà-Montagut, X.; Sarrat-González, D.; Quintás, G.; Rodas, G. Predicting Injuries in Elite Female Football Players With Global-Positioning-System and Multiomics Data. *Int J Sports Physiol Perform* **2024**, *19*, 661–669, doi:10.1123/ijspp.2023-0184.

31. Massidda, M.; Flore, L.; Cugia, P.; Piras, F.; Scorcu, M.; Kikuchi, N.; Cięszczyk, P.; Maciejewska-Skrendo, A.; Tocco, F.; Calò, C.M. Association Between Total Genotype Score and Muscle Injuries in Top-Level Football Players: A Pilot Study. *Sports Med Open* **2024**, *10*, 22, doi:10.1186/s40798-024-00682-z.

32. McAuley, A.B.T.; Hughes, D.C.; Tsaprouni, L.G.; Varley, I.; Suraci, B.; Bradley, B.; Baker, J.; Herbert, A.J.; Kelly, A.L. Genetic Associations With Acceleration, Change of Direction, Jump Height, and Speed in English Academy Football Players. *J Strength Cond Res* **2024**, *38*, 350–359, doi:10.1519/JSC.0000000000004634.

33. Maestro, A.; Del Coso, J.; Aguilar-Navarro, M.; Gutiérrez-Hellín, J.; Morencos, E.; Revuelta, G.; Ruiz Casares, E.; Perucho, T.; Varillas-Delgado, D. Genetic Profile in Genes Associated with Muscle Injuries and Injury Etiology in Professional Soccer Players. *Front Genet* **2022**, *13*, 1035899, doi:10.3389/fgene.2022.1035899.

34. Del Coso, J.; Rodas, G.; Buil, M.Á.; Sánchez-Sánchez, J.; López, P.; González-Ródenas, J.; Gasulla-Anglés, P.; López-Samanes, Á.; Hernández-Sánchez, S.; Iztueta, A.; et al. Association of the ACTN3 Rs1815739 Polymorphism with Physical Performance and Injury Incidence in Professional Women Football Players. *Genes (Basel)* **2022**, *13*, 1635, doi:10.3390/genes13091635.

35. Diogenes, M.E.L.; Bezerra, F.F.; Cabello, G.M.K.; Cabello, P.H.; Mendonça, L.M.C.; Oliveira Júnior, A.V.; Donangelo, C.M. Vitamin D Receptor Gene FokI Polymorphisms Influence Bone Mass in Adolescent Football (Soccer) Players. *Eur J Appl Physiol* **2010**, *108*, 31–38, doi:10.1007/s00421-009-1191-6.

36. Falahati, A.; Arazi, H. Cardiac Biomarker Responses Following High-Intensity Interval and Continuous Exercise: The Influence of ACE-I/D Gene Polymorphism and Training Status in Men. *Physiol Genomics* **2024**, *56*, 436–444, doi:10.1152/physiolgenomics.00129.2023.

37. Meckel, Y.; Eliakim, A.; Nemet, D.; Levin, N.; Ben-Zaken, S. PPARD CC and ACTN3 RR Genotype Prevalence among Elite Soccer Players. *Science and Medicine in Football* **2020**, *4*, 156–161, doi:10.1080/24733938.2019.1677936.

38. Monnerat, G.; Maior, A.S.; Tannure, M.; Back, L.K.F.C.; Santos, C.G.M. Single-Nucleotide-Polymorphism-Panel Population-Genetics Approach Based on the 1000 Genomes Database and Elite Soccer Players. *Int J Sports Physiol Perform* **2019**, *14*, 711–717, doi:10.1123/ijspp.2018-0715.

39. Varley, I.; Hughes, D.C.; Greeves, J.P.; Fraser, W.D.; Sale, C. SNPs in the Vicinity of P2X7R, RANK/RANKL/OPG and Wnt Signalling Pathways and Their Association with Bone Phenotypes in Academy Footballers. *Bone* **2018**, *108*, 179–185, doi:10.1016/j.bone.2018.01.007.

40. Terrell, T.R.; Bostick, R.M.; Abramson, R.; Xie, D.; Barfield, W.; Cantu, R.; Stanek, M.; Ewing, T. APOE, APOE Promoter, and Tau Genotypes and Risk for Concussion in College Athletes. *Clinical Journal of Sport Medicine* **2008**, *18*, 10, doi:10.1097/JSM.0b013e31815c1d4c.

41. Gouveia, M.M.S.; do Nascimento, M.B.A.; Crispim, A.C.; da Rocha, E.R.; Dos Santos, M.P.P.; Bento, E. de S.; De Aquino, T.M.; Balikian, P.; Rodrigues, N.A.; Ataide-Silva, T.; et al. Metabolomic Profiling of Elite Female Soccer Players: Urinary Biomarkers over a Championship Season. *Metabolomics* **2024**, *20*, 101, doi:10.1007/s11306-024-02164-5.

42. Flore, L.; Robledo, R.; Dettori, L.; Scorcu, M.; Francalacci, P.; Tocco, F.; Massidda, M.; Calò, C.M. Association of VDR Polymorphisms with Muscle Mass Development in Elite Young Soccer Players: A Pilot Study. *Sports (Basel)* **2024**, *12*, 253, doi:10.3390/sports12090253.

43. Fagundes, L.H.S.; Pinheiro, G. de S.; Pimenta, E.M.; Amorim, C.E.N.; Souza, R.P. de; Costa, V.T. da Association of the MuRF-1/TRIM63 Polymorphism with Muscle Injuries in Professional Soccer Players. *Retos* **2024**, *57*, 205–212, doi:10.47197/retos.v57.104261.

44. González-García, J.; Varillas-Delgado, D. The Relationship between Genetic Variability and Seasonal Changes in Vertical Jump Performance in Amateur Soccer Players. *Applied Sciences* **2024**, *14*, 6145, doi:10.3390/app14146145.

45. Varillas-Delgado, D. Association of Genetic Profile with Muscle Mass Gain and Muscle Injury Prevention in Professional Football Players after Creatine Supplementation. *Nutrients* **2024**, *16*, 2511, doi:10.3390/nu16152511.

46. Del Coso, J.; Rodas, G.; Soler-Aguinaga, A.; López-Del Campo, R.; Resta, R.; González-Rodenas, J.; Ferrandis, J.; Moreno-Pérez, V. ACTN3 XX Genotype Negatively Affects Running Performance and Increases Muscle Injury Incidence in LaLiga Football Players. *Genes (Basel)* **2024**, *15*, 386, doi:10.3390/genes15030386.

47. Pimenta, E.M.; Santos, C.G.M. dos; Assis, M.G. de; Veneroso, C.E.; Soalheiro, I.; Serpa, T.K.F. TTN-AS1 Genotype (Rs1001238) and Its Influence on Inflammatory Responses in Muscle Tissues in Soccer Players. *Braz. arch. biol. technol.* **2024**, *67*, e24231067, doi:https://doi.org/10.1590/1678-4324-2024231067.

48. de Almeida, K.Y.; Zempo, H.; Saito, M.; Cetolin, T.; Dos Santos Guimarães, R.; Marrero, A.R.; Aguiar, A.S.; Kikuchi, N. Influence of ACTN3 R577X Polymorphism on Blood Creatine Kinase Levels Relative to Number of Sprints in Brazilian Professional Soccer Players. *Genes (Basel)* **2024**, *15*, 896, doi:10.3390/genes15070896.

49. Albuquerque, M.R.; Cunha, A.E. da S.; Ferreira de Araújo, J.L.; Guimarães, R.D.S.; Rocha, M.R.C.C.; Mesquita, P.H.C.; Pimenta, E.M.; Pedra de Souza, R. Relative Age Effect and ACTN3 R577X and ACE I/D Polymorphisms in Brazilian Football Players: An Association Genetic Study. *Res Q Exerc Sport* **2025**, *96*, 327–337, doi:10.1080/02701367.2024.2407883.

50. Varillas-Delgado, D. Genes Associated with Muscle, Tendon and Ligament Injury Epidemiology in Women's Amateur Football Players. *Applied Sciences* **2024**, *14*, 1980, doi:10.3390/app14051980.

51. Bülbül, A.; Dereceli, Ç.; Eraslan, M.; Bozkurt, N.; Şahin, K.; Eliöz, M.; Taşdemir, D.Ş.; Güler, Y.E. Genetic Susceptibility of MMP3 (Rs3025058) Variant Allele in Male Football Players with Multiple ACL Surgeries: A PCR-RFLP Analysis Available online: https://www.jomh.org/articles/10.22514/jomh.2024.177 (accessed on 29 August 2025).

52. Varillas-Delgado, D. Influence of Genetic Polymorphisms and Biochemical Biomarkers on Response to Nutritional Iron Supplementation and Performance in a Professional Football Team: A Pilot Longitudinal Study. *Nutrients* **2025**, *17*, 1379, doi:10.3390/nu17081379.

53. Gouveia, M.M.S.; Nascimento, M.B.A.; Crispim, A.C.; da Rocha, E.R.; Santos, M.P.P.; Bento, E.S.; Aquino, T.M.; Balikian, P.; Rodrigues, N.A.; Ataide-Silva, T.; et al. Metabolomic Profiles and Antioxidant Intake in Female Soccer Players: A Cross-Sectional Study. *Eur J Appl Physiol* **2025**, doi:10.1007/s00421-025-05808-z.

54. Malefo, N.; Naidoo, C.M.; Mphephu, M.M.; Motshudi, M.C.; Mkolo, N.M. Metabolomics Approach for Sweat Mapping the Performance of Soccer Players in Pretoria, South Africa. *Applied Sciences* **2025**, *15*, 4588, doi:10.3390/app15084588.

55. Rodas, G.; Ferrer, E.; Sanjuan, J.D.; Quintás, G. UPLC-MS and Multivariate Analysis Reveal Metabolic Pathway Adaptations to Training in Professional Football Players. *Talanta* **2025**, *291*, 127893, doi:10.1016/j.talanta.2025.127893.

56. Manchón-Davó, M.; Del Coso, J.; Vera-Garcia, F.J.; González-Rodenas, J.; Miralles-Iborra, A.; Rodas, G.; López-Del Campo, R.; Moreno-Pérez, V. Association Between the COL5A1 Rs12722 Genotype and the Prevalence of Anterior Cruciate Ligament Rupture in Professional Football Players. *Genes* **2025**, *16*, 649, doi:10.3390/genes16060649.

57. Bulgay, C.; Cepicka, L.; Dalip, M.; Yıldırım, S.; Ceylan, H.I.; Yılmaz, Ö.Ö.; Ulucan, K.; Badicu, G.; Cerit, M. The Relationships between ACTN3 Rs1815739 and PPARA-α Rs4253778 Gene Polymorphisms and Athletic Performance Characteristics in Professional Soccer Players. *BMC Sports Sci Med Rehabil* **2023**, *15*, 121, doi:10.1186/s13102-023-00733-0.

58. Kanope, T.; Santos, C.G.M.; Marinho, F.; Monnerat, G.; Campos-Junior, M.; da Fonseca, A.C.P.; Zembrzuski, V.M.; de Assis, M.; Pfaffl, M.W.; Pimenta, E. Replicative Study in Performance-Related Genes of Brazilian Elite Soccer Players Highlights Genetic Differences from African Ancestry and Similarities between Professional and U20 Youth Athletes. *Genes (Basel)* **2023**, *14*, 1446, doi:10.3390/genes14071446.

59. Yang, S.; Lin, W.; Jia, M.; Chen, H. Association between ACE and ACTN3 Genes Polymorphisms and Athletic Performance in Elite and Sub-Elite Chinese Youth Male Football Players. *PeerJ* **2023**, *11*, e14893, doi:10.7717/peerj.14893.

60. McAuley, A.B.; Hughes, D.C.; Tsaprouni, L.G.; Varley, I.; Suraci, B.; Baker, J.; Herbert, A.J.; Kelly, A.L. Genetic Associations with Technical Capabilities in English Academy Football Players: A Preliminary Study. *J Sports Med Phys Fitness* **2023**, *63*, 230–240, doi:10.23736/S0022-4707.22.13945-9.

61. Ruzic, L.; Radman, I.; Matkovic, B.R. Differences in ACTN3, ACE, and ADBR3 Polymorphisms between Croatian National Team and Non-National Team Elite Soccer Players. *Balt J Health Phys Act* **2023**, *15*, Article6, doi:10.29359/BJHPA.15.2.06.

62. Kurtuluş, M.; Keskin, K.; Gunay, M.; Kesici, T.; Gökdemir, K. Genetic Differences in Peroxisome Proliferator-Activated Receptor Alpha Gene in Endurance Athletes (Long Distance Runners) and Power/Endurance Athletes (Wrestlers, Football Players). *JBACHS* **2023**, *7*, 723–730, doi:10.30621/jbachs.1191220.

63. Yang, S.; Lin, W.; Jia, M.; Chen, H. Association between ACTN3 R577x and the Physical Performance of Chinese 13 to 15-Year-Old Elite and Sub-Elite Football Players at Different Positions. *Front Genet* **2023**, *14*, 1038075, doi:10.3389/fgene.2023.1038075.

64. Petr, M.; Thiel, D.; Kateřina, K.; Brož, P.; Malý, T.; Zahálka, F.; Vostatková, P.; Wilk, M.; Chycki, J.; Stastny, P. Speed and Power-Related Gene Polymorphisms Associated with Playing Position in Elite Soccer Players. *Biol Sport* **2022**, *39*, 355–366, doi:10.5114/biolsport.2022.105333.

65. Soriano, S.; Curry, K.; Sadrameli, S.S.; Wang, Q.; Nute, M.; Reeves, E.; Kabir, R.; Wiese, J.; Criswell, A.; Schodrof, S.; et al. Alterations to the Gut Microbiome after Sport-Related Concussion in a Collegiate Football Players Cohort: A Pilot Study. *Brain Behav Immun Health* **2022**, *21*, 100438, doi:10.1016/j.bbih.2022.100438.

66. Varillas-Delgado, D.; Morencos, E.; Gutiérrez-Hellín, J.; Aguilar-Navarro, M.; Muñoz, A.; Mendoza Láiz, N.; Perucho, T.; Maestro, A.; Tellería-Orriols, J.J. Genetic Profiles to Identify Talents in Elite Endurance Athletes and Professional Football Players. *PLoS One* **2022**, *17*, e0274880, doi:10.1371/journal.pone.0274880.

67. de Almeida, K.Y.; Cetolin, T.; Marrero, A.R.; Aguiar Junior, A.S.; Mohr, P.; Kikuchi, N. A Pilot Study on the Prediction of Non-Contact Muscle Injuries Based on ACTN3 R577X and ACE I/D Polymorphisms in Professional Soccer Athletes. *Genes (Basel)* **2022**, *13*, 2009, doi:10.3390/genes13112009.

68. Kim, H.-Y.; Lee, J.-D.; Lee, Y.-H.; Seo, S.-W.; Lee, H.-S.; Kim, S.; Kim, K.-B. Urinary Metabolomics in Young Soccer Players after Winter Training Season. *Metabolites* **2022**, *12*, 1283, doi:10.3390/metabo12121283.

69. Viciani, E.; Barone, M.; Bongiovanni, T.; Quercia, S.; Di Gesu, R.; Pasta, G.; Manetti, P.; Iaia, F.M.; Trecroci, A.; Rampelli, S.; et al. Fecal Microbiota Monitoring in Elite Soccer Players Along the 2019-2020 Competitive Season. *Int J Sports Med* **2022**, *43*, 1137–1147, doi:10.1055/a-1858-1810.

70. da Cruz, J.P.; Dos Santos, F.N.; Rasteiro, F.M.; Marostegan, A.B.; Manchado-Gobatto, F.B.; Gobatto, C.A. A Metabolomic Approach and Traditional Physical Assessments to Compare U22 Soccer Players According to Their Competitive Level. *Biology (Basel)* **2022**, *11*, 1103, doi:10.3390/biology11081103.

71. McAuley, A.B.T.; Hughes, D.C.; Tsaprouni, L.G.; Varley, I.; Suraci, B.; Baker, J.; Herbert, A.J.; Kelly, A.L. Genetic Associations with Personality and Mental Toughness Profiles of English Academy Football Players: An Exploratory Study. *Psychology of Sport and Exercise* **2022**, *61*, 102209, doi:10.1016/j.psychsport.2022.102209.

72. Zileli, R.; Söyler, M.; Diker, G.; Özkamçı, H.; Bayrakdaroglu, S.; Sever, M.O.; Can, I.; Ön, S.; Aslan, B.T.; Ulucan, K.; et al. The Distinguishing Factor in Soccer Players Is Aerobic Performance, Not the ACTN3 Gene. *Int. J. Morphol.* **2023**, *41*, 1564–1569, doi:10.4067/S0717-95022023000501564.

73. Rodas, G.; Ferrer, E.; Reche, X.; Sanjuan-Herráez, J.D.; McCall, A.; Quintás, G. A Targeted Metabolic Analysis of Football Players and Its Association to Player Load: Comparison between Women and Men Profiles. *Front Physiol* **2022**, *13*, 923608, doi:10.3389/fphys.2022.923608.

74. Jacob, Y.; Anderton, R.S.; Cochrane Wilkie, J.L.; Rogalski, B.; Laws, S.M.; Jones, A.; Spiteri, T.; Hince, D.; Hart, N.H. Genetic Variants within NOGGIN, COL1A1, COL5A1, and IGF2 Are Associated with Musculoskeletal Injuries in Elite Male Australian Football League Players: A Preliminary Study. *Sports Med Open* **2022**, *8*, 126, doi:10.1186/s40798-022-00522-y.

75. Végh, D.; Reichwalderová, K.; Slaninová, M.; Vavák, M. The Effect of Selected Polymorphisms of the ACTN3, ACE, HIF1A and PPARA Genes on the Immediate Supercompensation Training Effect of Elite Slovak Endurance Runners and Football Players. *Genes (Basel)* **2022**, *13*, 1525, doi:10.3390/genes13091525.

76. Varillas-Delgado, D.; Morencos, E.; Gutierrez-Hellín, J.; Aguilar-Navarro, M.; Maestro, A.; Perucho, T.; Coso, J.D. Association of the CKM Rs8111989 Polymorphism with Injury Epidemiology in Football Players. *Int J Sports Med* **2023**, *44*, 145–152, doi:10.1055/a-1945-8982.

77. Papa, L.; Walter, A.E.; Wilkes, J.R.; Clonts, H.S.; Johnson, B.; Slobounov, S.M. Effect of Player Position on Serum Biomarkers during Participation in a Season of Collegiate Football. *J Neurotrauma* **2022**, *39*, 1339–1348, doi:10.1089/neu.2022.0083.

78. Coelho, D.B.; Pimenta, E.M.; Rosse, I.C.; de Oliveira, E.C.; Becker, L.K.; Ferreira-Júnior, J.B.; Lopes, L.M.; Carvalho, M.R.; Silami-Garcia, E. Polymorphism of the Angiotensin Converting Enzyme Gene (ACE-I/D) Differentiates the Aerobic and Speed Performance of Football Players. *J Sports Med Phys Fitness* **2022**, *62*, 192–198, doi:10.23736/S0022-4707.21.12060-2.

79. Jacob, Y.; Hart, N.H.; Cochrane, J.L.; Spiteri, T.; Laws, S.M.; Jones, A.; Rogalski, B.; Kenna, J.; Anderton, R.S. ACTN3 (R577X) Genotype Is Associated With Australian Football League Players. *J Strength Cond Res* **2022**, *36*, 573–576, doi:10.1519/JSC.0000000000003458.

80. Kanope, T.; Pimenta, E.M.; Veneroso, C.; Coelho, D.; Oliveira, L.F.; Silami-Garcia, E.; Morandi, R.F.; Carvalho, M.R.S.; Rosse, I.C. Is Lin28a Polymorphism Associated with Endurance Performance in Soccer Players? *Sport Sci Health* **2022**, *18*, 349–355, doi:10.1007/s11332-021-00812-0.

81. Bulgay, C.; Bragazzi, N.L.; Gök, O.; Çakır, V.O.; Tuncer, S.Y.; Polat, T.; Ulucan, K.; Ceylan, H.İ.; Dalip, M.; Kocak, M.S.; et al. Assessment of Male Football Players' Physical Fitness Levels Based on Certain Gene (AGT Rs699 & IL-6 Rs1800795) Polymorphisms Available online: https://www.jomh.org/articles/10.22514/jomh.2025.050 (accessed on 29 August 2025).

82. Hall, E.C.R.; Baumert, P.; Larruskain, J.; Gil, S.M.; Lekue, J.A.; Rienzi, E.; Moreno, S.; Tannure, M.; Murtagh, C.F.; Ade, J.D.; et al. The Genetic Association with Injury Risk in Male Academy Soccer Players Depends on Maturity Status. *Scand J Med Sci Sports* **2022**, *32*, 338–350, doi:10.1111/sms.14077.

83. McAuley, A.B.T.; Hughes, D.C.; Tsaprouni, L.G.; Varley, I.; Suraci, B.; Baker, J.; Herbert, A.J.; Kelly, A.L. Genetic Variations between Youth and Professional Development Phase English Academy Football Players. *Genes* **2022**, *13*, 2001, doi:10.3390/genes13112001.

84. Mohd Fazli, N.E.; Raja Azidin, R.M.F.; Teh, L.K.; Salleh, M.Z. Correlations between Sports-Related Polygenic Profiles, Postural Stability, Power and Strength Performances of Elite Football Players. *Sport Sci Health* **2022**, *18*, 147–154, doi:10.1007/s11332-021-00786-z.

85. Rodas, G.; Moreno-Pérez, V.; Del Coso, J.; Florit, D.; Osaba, L.; Lucia, A. Alpha-Actinin-3 Deficiency Might Affect Recovery from Non-Contact Muscle Injuries: Preliminary Findings in a Top-Level Soccer Team. *Genes (Basel)* **2021**, *12*, 769, doi:10.3390/genes12050769.

86. Marinho, A.H.; Sousa, F.A. de B.; Vilela, R. de A.M.P.; Balikian, P.; de Souza Bento, E.; de Mendonça Aquino, T.; Crispim, A.; Ataide-Silva, T.; de Araujo, G.G. The Rating of Perceived Exertion Is Able to Differentiate the Post-Matches Metabolomic Profile of Elite U-20 Soccer Players. *Eur J Appl Physiol* **2022**, *122*, 371–382, doi:10.1007/s00421-021-04838-7.

87. Murtagh, C.F.; Brownlee, T.E.; Rienzi, E.; Roquero, S.; Moreno, S.; Huertas, G.; Lugioratto, G.; Baumert, P.; Turner, D.C.; Lee, D.; et al. The Genetic Profile of Elite Youth Soccer Players and Its Association with Power and Speed Depends on Maturity Status. *PLoS One* **2020**, *15*, e0234458, doi:10.1371/journal.pone.0234458.

88. Wei, Q. The ACE and ACTN3 Polymorphisms in Female Soccer Athletes. *Genes Environ* **2021**, *43*, 5, doi:10.1186/s41021-021-00177-3.

89. Clos, E.; Pruna, R.; Lundblad, M.; Artells, R.; Maffulli, N. ACTN3's R577X Single Nucleotide Polymorphism Allele Distribution Differs Significantly in Professional Football Players According to Their Field Position. *Med Princ Pract* **2021**, *30*, 92–97, doi:10.1159/000509089.

90. Muhan, A.; Polat, T.; Yılmaz, Ö.Ö.; Aslan, B.T.; Ulucan, K. Futbolcularda ACTN3 rs1815739 Polimorfizmi, Fiziksel Özellikler ve Mevki İlişkisinin Araştırılması: Bir Takım Örneklemi. *Research in Sport Education and Sciences* **2023**, *25*, 14–18, doi:10.5152/JPESS.2023.221840.

91. Suraci, B.R.; Quigley, C.; Thelwell, R.C.; Milligan, G.S. A Comparison of Training Modality and Total Genotype Scores to Enhance Sport-Specific Biomotor Abilities in Under 19 Male Soccer Players. *J Strength Cond Res* **2021**, *35*, 154–161, doi:10.1519/JSC.0000000000003299.

92. Massidda, M.; Flore, L.; Kikuchi, N.; Scorcu, M.; Piras, F.; Cugia, P.; Cięszczyk, P.; Tocco, F.; Calò, C.M. Influence of the MCT1-T1470A Polymorphism (Rs1049434) on Repeated Sprint Ability and Blood Lactate Accumulation in Elite Football Players: A Pilot Study. *Eur J Appl Physiol* **2021**, *121*, 3399–3408, doi:10.1007/s00421-021-04797-z.

93. Massidda, M.; Voisin, S.; Culigioni, C.; Piras, F.; Cugia, P.; Yan, X.; Eynon, N.; Calò, C.M. ACTN3 R577X Polymorphism Is Associated With the Incidence and Severity of Injuries in Professional Football Players. *Clin J Sport Med* **2019**, *29*, 57–61, doi:10.1097/JSM.0000000000000487.

94. Pickering, C.; Suraci, B.; Semenova, E.A.; Boulygina, E.A.; Kostryukova, E.S.; Kulemin, N.A.; Borisov, O.V.; Khabibova, S.A.; Larin, A.K.; Pavlenko, A.V.; et al. A Genome-Wide Association Study of Sprint Performance in Elite Youth Football Players. *J Strength Cond Res* **2019**, *33*, 2344–2351, doi:10.1519/JSC.0000000000003259.

95. Petrovic, T.; Zdravkovic, M.; Djelic, M.; Gavrilovic, T.; Mihailovic, Z.; Atanasijevic, N.; Stojkovic, O. Influence of ACE and ACTN3 Genes Polymorphisms on Cardiovascular Adaptation in Female Football Players. *Genetika* **2022**, *54*, 1035–1047, doi:10.2298/GENSR2203035P.

96. Hunter, L.E.; Freudenberg-Hua, Y.; Davies, P.; Kim, M.; Lipton, R.B.; Stewart, W.F.; Srinivasan, P.; Hu, S.; Lipton, M.L. Associations of Apolipoprotein E E4 Genotype and Ball Heading With Verbal Memory in Amateur Soccer Players. *JAMA Neurol* **2020**, *77*, 419–426, doi:10.1001/jamaneurol.2019.4828.

97. Pitti, E.; Petrella, G.; Di Marino, S.; Summa, V.; Perrone, M.; D'Ottavio, S.; Bernardini, A.; Cicero, D.O. Salivary Metabolome and Soccer Match: Challenges for Understanding Exercise Induced Changes. *Metabolites* **2019**, *9*, 141, doi:10.3390/metabo9070141.

98. Muraoka, S.; Jedrychowski, M.P.; Tatebe, H.; DeLeo, A.M.; Ikezu, S.; Tokuda, T.; Gygi, S.P.; Stern, R.A.; Ikezu, T. Proteomic Profiling of Extracellular Vesicles Isolated From Cerebrospinal Fluid of Former National Football League Players at Risk for Chronic Traumatic Encephalopathy. *Front Neurosci* **2019**, *13*, 1059, doi:10.3389/fnins.2019.01059.

99. Massidda, M.; Miyamoto-Mikami, E.; Kumagai, H.; Ikeda, H.; Shimasaki, Y.; Yoshimura, M.; Cugia, P.; Piras, F.; Scorcu, M.; Kikuchi, N.; et al. Association between the ACE I/D Polymorphism and Muscle Injuries in Italian and Japanese Elite Football Players. *J Sports Sci* **2020**, *38*, 2423–2429, doi:10.1080/02640414.2020.1787683.

100. Jacob, Y.; Anderton, R.S.; Cochrane Wilkie, J.L.; Rogalski, B.; Laws, S.M.; Jones, A.; Spiteri, T.; Hart, N.H. Association of Genetic Variances in ADRB1 and PPARGC1a with Two-Kilometre Running Time-Trial Performance in Australian Football League Players: A Preliminary Study. *Sports* **2021**, *9*, 22, doi:10.3390/sports9020022.

101. Alzharani, M.A.; Alshuwaier, G.O.; Aljaloud, K.S.; Al-Tannak, N.F.; Watson, D.G. Metabolomics Profiling of Plasma, Urine and Saliva after Short Term Training in Young Professional Football Players in Saudi Arabia. *Sci Rep* **2020**, *10*, 19759, doi:10.1038/s41598-020-75755-6.

102. Cao, B.; Liu, S.; Yang, L.; Chi, A. Changes of Differential Urinary Metabolites after High-Intensive Training in Teenage Football Players. *Biomed Res Int* **2020**, *2020*, 2073803, doi:10.1155/2020/2073803.

103. Larruskain, J.; Celorrio, D.; Barrio, I.; Odriozola, A.; Gil, S.M.; Fernandez-Lopez, J.R.; Nozal, R.; Ortuzar, I.; Lekue, J.A.; Aznar, J.M. Genetic Variants and Hamstring Injury in Soccer: An Association and Validation Study. *Med Sci Sports Exerc* **2018**, *50*, 361–368, doi:10.1249/MSS.0000000000001434.

104. Clos, E.; Pruna, R.; Lundblad, M.; Artells, R.; Esquirol Caussa, J. ACTN3 Single Nucleotide Polymorphism Is Associated with Non-Contact Musculoskeletal Soft-Tissue Injury Incidence in Elite Professional Football Players. *Knee Surg Sports Traumatol Arthrosc* **2019**, *27*, 4055–4061, doi:10.1007/s00167-019-05381-x.

105. Coelho, D.B.; Pimenta, E.M.; Rosse, I.C.; Veneroso, C.; Pussieldi, G.D.A.; Becker, L.K.; Oliveira, E.C.; Carvalho, M.R.S.; Silami-Garcia, E. Alpha-Actinin-3 R577X Polymorphism Influences Muscle Damage and Hormonal Responses After a Soccer Game. *J Strength Cond Res* **2019**, *33*, 2655–2664, doi:10.1519/JSC.0000000000002575.

106. Koku, F.E.; Karamızrak, S.O.; Çiftçi, A.S.; Taşlıdere, H.; Durmaz, B.; Çoğulu, Ö. The Relationship between ACTN3 R577X Gene Polymorphism and Physical Performance in Amateur Soccer Players and Sedentary Individuals. *Biol Sport* **2019**, *36*, 9–16, doi:10.5114/biolsport.2018.78900.

107. Pruna, R.; Artells, R.; Lundblad, M.; Maffulli, N. Genetic Biomarkers in Non-Contact Muscle Injuries in Elite Soccer Players. *Knee Surg Sports Traumatol Arthrosc* **2017**, *25*, 3311–3318, doi:10.1007/s00167-016-4081-6.

108. Dionísio, T.J.; Thiengo, C.R.; Brozoski, D.T.; Dionísio, E.J.; Talamoni, G.A.; Silva, R.B.; Garlet, G.P.; Santos, C.F.; Amaral, S.L. The Influence of Genetic Polymorphisms on Performance and Cardiac and Hemodynamic Parameters among Brazilian Soccer Players. *Appl Physiol Nutr Metab* **2017**, *42*, 596–604, doi:10.1139/apnm-2016-0608.

109. Coelho, D.B.; Pimenta, E.M.; Rosse, I.C.; de Castro, B.M.; Becker, L.K.; de Oliveira, E.C.; Carvalho, M.R.S.; Garcia, E.S. Evidence for a Role of ACTN3 R577X Polymorphism in Football Player's Career Progression. *Int J Sports Med* **2018**, *39*, 1088–1093, doi:10.1055/a-0753-4973.

110. Jacob, Y.; Chivers, P.; Anderton, R.S. Genetic Predictors of Match Performance in Sub-Elite Australian Football Players: A Pilot Study. *J Exerc Sci Fit* **2019**, *17*, 41–46, doi:10.1016/j.jesf.2018.10.007.

111. Atabas, E.G.; Öksüzoğlu, A.Y.; Turel, S.; Akca, H. The Relationship of Polymorphism with Explosive Forces in ACTN3, ACE, and UCP3 Genes in Soccer Players. *Progress in Nutrition* **2020**, *22*, e2020048–e2020048, doi:10.23751/pn.v22i3.10728.

112. Prado, E.; Souza, G.H.M.F.; Pegurier, M.; Vieira, C.; Lima-Neto, A.B.M.; Assis, M.; Guedes, M.I.F.; Koblitz, M.G.B.; Ferreira, M.S.L.; Macedo, A.F.; et al. Non-Targeted Sportomics Analyses by Mass Spectrometry to Understand Exercise-Induced Metabolic Stress in Soccer Players. *International Journal of Mass Spectrometry* **2017**, *418*, 1–5, doi:10.1016/j.ijms.2017.02.002.

113. Domańska-Senderowska, D.; Szmigielska, P.; Snochowska, A.; Jastrzębski, Z.; Jegier, A.; Kiszałkiewicz, J.; Jastrzębska, J.; Pastuszak-Lewandoska, D.; Cięszczyk, P.; Suchanecka, A.; et al. Relationships between the Expression of the ACTN3 Gene and Explosive Power of Soccer Players. *J Hum Kinet* **2019**, *69*, 79–87, doi:10.2478/hukin-2019-0020.

114. Massidda, M.; Mendez-Villanueva, A.; Ginevičienė, V.; Proia, P.; Drozdovska, S.B.; Dosenko, V.; Scorcu, M.; Stula, A.; Sawczuk, M.; Cięszczyk, P.; et al. Association of Monocarboxylate Transporter-1 (MCT1) A1470T Polymorphism (Rs1049434) with Forward Football Player Status. *Int J Sports Med* **2018**, *39*, 1028–1034, doi:10.1055/a-0634-6387.

115. Coelho, D.B.; Pimenta, E.; Rosse, I.C.; Veneroso, C.; Becker, L.K.; Carvalho, M.R.; Pussieldi, G.; Silami-Garcia, E. The Alpha-Actinin-3 R577x Polymorphism and Physical Performance in Soccer Players. *J Sports Med Phys Fitness* **2016**, *56*, 241–248.

116. Jeremic, D.; Macuzic, I.Z.; Vulovic, M.; Stevanovic, J.; Radovanovic, D.; Varjacic, V.; Djordjevic, D. ACE/ACTN3 GENETIC POLYMORPHISMS AND ATHLETIC PERFORMANCE OF FEMALE SOCCER PLAYERS`. *Rev Bras Med Esporte* **2019**, *25*, 35–39, doi:https://doi.org/10.1590/1517-869220192501187684.

117. Galeandro, V.; Notarnicola, A.; Bianco, A.; Tafuri, S.; Russo, L.; Pesce, V.; Moretti, B.; Petruzzella, V. ACTN3/ACE Genotypes and Mitochondrial Genome in Professional Soccer Players' Performance. *J Biol Regul Homeost Agents* **2017**, *31*, 207–213.

118. Massidda, M.; Bachis, V.; Corrias, L.; Piras, F.; Scorcu, M.; Calò, C.M. Influence of the COL5A1 Rs12722 on Musculoskeletal Injuries in Professional Soccer Players. *J Sports Med Phys Fitness* **2015**, *55*, 1348–1353.

119. Sun, Z.; Cięszczyk, P.; Lulińska, E.; Dzitkowska-Zabielska, M.; Johne, M.; Humińska-Lisowska, K.; Michałowska-Sawczyn, M.; Ficek, K.; Leońska-Duniec, A.; Mastalerz, A.; et al. Are COL22A1 Gene Polymorphisms Rs11784270 and Rs6577958 Associated with Susceptibility to a Non-Contact Anterior Cruciate Ligament Injury in Polish Athletes? *Int J Environ Res Public Health* **2022**, *20*, 515, doi:10.3390/ijerph20010515.

120. Massidda, M.; Eynon, N.; Bachis, V.; Corrias, L.; Culigioni, C.; Cugia, P.; Scorcu, M.; Calò, C.M. Association Between MCT1 A1470T Polymorphism and Fat-Free Mass in Well-Trained Young Soccer Players. *J Strength Cond Res* **2016**, *30*, 1171–1176, doi:10.1519/JSC.0000000000001176.

121. Hunter, L.E.; Freudenberg-Hua, Y.; Davies, P.; Kim, M.; Fleysher, R.; Stewart, W.F.; Lipton, R.B.; Lipton, M.L. BDNF Val66Met Positive Players Demonstrate Diffusion Tensor Imaging Consistent With Impaired Myelination Associated With High Levels of Soccer Heading: Indication of a Potential Gene-Environment Interaction Mechanism. *Front Neurol* **2019**, *10*, 1297, doi:10.3389/fneur.2019.01297.

122. Jacob, Y.; Cripps, A.; Evans, T.; Chivers, P.T.; Joyce, C.; Anderton, R.S. Identification of Genetic Markers for Skill and Athleticism in Sub-Elite Australian Football Players: A Pilot Study. *J Sports Med Phys Fitness* **2018**, *58*, 241–248, doi:10.23736/S0022-4707.16.06647-0.

123. Coelho, D.B.; Pimenta, E.; Rosse, I.C.; Veneroso, C.; Pussieldi, G.; Becker, L.K.; Carvalho, M.-R.; Silami-Garcia, E. Angiotensin-Converting Enzyme (ACE-I/D) Polymorphism Frequency in Brazilian Soccer Players. *Appl Physiol Nutr Metab* **2016**, *41*, 692–694, doi:10.1139/apnm-2015-0514.

124. Ra, S.-G.; Maeda, S.; Higashino, R.; Imai, T.; Miyakawa, S. Metabolomics of Salivary Fatigue Markers in Soccer Players after Consecutive Games. *Appl Physiol Nutr Metab* **2014**, *39*, 1120–1126, doi:10.1139/apnm-2013-0546.

125. Dinç, N.; Yücel, S.B.; Taneli, F.; Sayın, M.V. The Effect of the MTHFR C677T Mutation on Athletic Performance and the Homocysteine Level of Soccer Players and Sedentary Individuals. *J Hum Kinet* **2016**, *51*, 61–69, doi:10.1515/hukin-2015-0171.

126. Egorova, E.S.; Borisova, A.V.; Mustafina, L.J.; Arkhipova, A.A.; Gabbasov, R.T.; Druzhevskaya, A.M.; Astratenkova, I.V.; Ahmetov, I.I. The Polygenic Profile of Russian Football Players. *J Sports Sci* **2014**, *32*, 1286–1293, doi:10.1080/02640414.2014.898853.

127. Pruna, R.; Ribas, J.; Montoro, J.B.; Artells, R. The Impact of Single Nucleotide Polymorphisms on Patterns of Non-Contact Musculoskeletal Soft Tissue Injuries in a Football Player Population According to Ethnicity. *Med Clin (Barc)* **2015**, *144*, 105–110, doi:10.1016/j.medcli.2013.09.026.

128. Pimenta, E.M.; Coelho, D.B.; Veneroso, C.E.; Barros Coelho, E.J.; Cruz, I.R.; Morandi, R.F.; De A Pussieldi, G.; Carvalho, M.R.S.; Garcia, E.S.; De Paz Fernández, J.A. Effect of ACTN3 Gene on Strength and Endurance in Soccer Players. *J Strength Cond Res* **2013**, *27*, 3286–3292, doi:10.1519/JSC.0b013e3182915e66.

129. Gineviciene, V.; Jakaitiene, A.; Tubelis, L.; Kucinskas, V. Variation in the ACE, PPARGC1A and PPARA Genes in Lithuanian Football Players. *Eur J Sport Sci* **2014**, *14 Suppl 1*, S289-295, doi:10.1080/17461391.2012.691117.

130. La Montagna, R.; Canonico, R.; Alfano, L.; Bucci, E.; Boffo, S.; Staiano, L.; Fulco, B.; D'Andrea, E.; De Nicola, A.; Maiorano, P.; et al. Genomic Analysis Reveals Association of Specific SNPs with Athletic Performance and Susceptibility to Injuries in Professional Soccer Players. *J Cell Physiol* **2020**, *235*, 2139–2148, doi:10.1002/jcp.29118.

131. Ficek, K.; Cieszczyk, P.; Kaczmarczyk, M.; Maciejewska-Karłowska, A.; Sawczuk, M.; Cholewinski, J.; Leonska-Duniec, A.; Stepien-Slodkowska, M.; Zarebska, A.; Stepto, N.K.; et al. Gene Variants within the COL1A1 Gene Are Associated with Reduced Anterior Cruciate Ligament Injury in Professional Soccer Players. *J Sci Med Sport* **2013**, *16*, 396–400, doi:10.1016/j.jsams.2012.10.004.

132. Pruna, R.; Artells, R.; Ribas, J.; Montoro, B.; Cos, F.; Muñoz, C.; Rodas, G.; Maffulli, N. Single Nucleotide Polymorphisms Associated with Non-Contact Soft Tissue Injuries in Elite Professional Soccer Players: Influence on Degree of Injury and Recovery Time. *BMC Musculoskeletal Disorders* **2013**, *14*, 221, doi:10.1186/1471-2474-14-221.

133. Eynon, N.; Ruiz, J.R.; Yvert, T.; Santiago, C.; Gómez-Gallego, F.; Lucia, A.; Birk, R. The C Allele in NOS3 -786 T/C Polymorphism Is Associated with Elite Soccer Player's Status. *Int J Sports Med* **2012**, *33*, 521–524, doi:10.1055/s-0032-1306337.

134. Massidda, M.; Corrias, L.; Ibba, G.; Scorcu, M.; Vona, G.; Calò, C.M. Genetic Markers and Explosive Leg-Muscle Strength in Elite Italian Soccer Players. *J Sports Med Phys Fitness* **2012**, *52*, 328–334.

135. Micheli, M.L.; Gulisano, M.; Morucci, G.; Punzi, T.; Ruggiero, M.; Ceroti, M.; Marella, M.; Castellini, E.; Pacini, S. Angiotensin-Converting Enzyme/Vitamin D Receptor Gene Polymorphisms and Bioelectrical Impedance Analysis in Predicting Athletic Performances of Italian Young Soccer Players. *J Strength Cond Res* **2011**, *25*, 2084–2091, doi:10.1519/JSC.0b013e31820238aa.

136. Juffer, P.; Furrer, R.; González-Freire, M.; Santiago, C.; Verde, Z.; Serratosa, L.; Morate, F.J.; Rubio, J.C.; Martin, M.A.; Ruiz, J.R.; et al. Genotype Distributions in Top-Level Soccer Players: A Role for ACE? *Int J Sports Med* **2009**, *30*, 387–392, doi:10.1055/s-0028-1105931.

137. Martín-Sánchez, F.J.; Villalón, J.M.; Zamorano-León, J.J.; Rosas, L.F.; Proietti, R.; Mateos-Caceres, P.J.; González-Armengol, J.J.; Villarroel, P.; Macaya, C.; López-Farré, A.J. Functional Status and Inflammation after Preseason Training Program in Professional and Recreational Soccer Players: A Proteomic Approach. *J Sports Sci Med* **2011**, *10*, 45–51.

138. Atli, M. Serum Paraoxonase Activity and Lipid Hydroperoxide Levels in Adult Football Players after Three Days Football Tournament. *Afr Health Sci* **2013**, *13*, 565–570, doi:10.4314/ahs.v13i3.6.

139. Rodas, G.; Cáceres, A.; Ferrer, E.; Balagué-Dobón, L.; Osaba, L.; Lucia, A.; González, J.R. Sex Differences in the Association between Risk of Anterior Cruciate Ligament Rupture and COL5A1 Polymorphisms in Elite Footballers. *Genes (Basel)* **2022**, *14*, 33, doi:10.3390/genes14010033.

140. Ang, G.; Raja Azidin, R.; Aiman, S.; Adi, S.; Yu, C.Y. Combined Influence of ACE and ACTN3 Polymorphisms on Vertical Jump Performance Following Resistance Training in Professional Footballers. *Sport Sciences for Health* **2023**, *20*, doi:10.1007/s11332-023-01146-9.
