# Supplementary material for: Socceromics: A Systematic Review of Omics Technologies to Optimize Performance and Health in Soccer
Source: Int J Mol Sci. 2026 Jan 12;27(2):749. doi: 10.3390/ijms27020749 (PMC12841393; doi:10.3390/ijms27020749)
Supplement: Supplementary file 1 [file ijms-27-00749-s001.zip › Table S3.pdf]

Table S3. Modified version of QUADOMICS

Items	Description
1	Were selection criteria clearly described?
3	Was the type of sample fully described?
4	Were the procedures and timing of biological sample collection with respect to clinical factors described with enough detail?
	4.1. Clinical and physiological factors
	4.2. Diagnostic and treatment procedures.
5	Were handling and pre-analytical procedures reported in sufficient detail and similar for the whole sample? And, if differences in procedures were reported, was their effect on the results assessed?
10	Was the execution of the index test described in sufficient detail to permit replication of the test?
11	Was the execution of the reference standard described in sufficient detail to permit its replication?
15	Were uninterpretable/intermediate test results reported?
16	Is it likely that the presence of overfitting was avoided?
